# High light-induced changes in whole-cell proteomic profile and its correlation with the organization of thylakoid super-complex in cyclic electron transport mutants of *Chlamydomonas reinhardtii*

**DOI:** 10.3389/fpls.2023.1198474

**Published:** 2023-07-07

**Authors:** Ranay Mohan Yadav, Sureshbabu Marriboina, Mohammad Yusuf Zamal, Jayendra Pandey, Rajagopal Subramanyam

**Affiliations:** Department of Plant Sciences, School of Life Sciences, University of Hyderabad, Hyderabad, India

**Keywords:** *Chlamydomonas reinhardtii*, cyclic electron transport, differentially abundant proteins, high light, LHCSR, PSBS, photosynthesis, thylakoid super-complex

## Abstract

Light and nutrients are essential components of photosynthesis. Activating the signaling cascades is critical in starting adaptive processes in response to high light. In this study, we have used wild-type (WT), cyclic electron transport (CET) mutants like Proton Gradient Regulation (PGR) (*PGRL1*), and *PGR5* to elucidate the actual role in regulation and assembly of photosynthetic pigment–protein complexes under high light. Here, we have correlated the biophysical, biochemical, and proteomic approaches to understand the targeted proteins and the organization of thylakoid pigment–protein complexes in the photoacclimation. The proteomic analysis showed that 320 proteins were significantly affected under high light compared to the control and are mainly involved in the photosynthetic electron transport chain, protein synthesis, metabolic process, glycolysis, and proteins involved in cytoskeleton assembly. Additionally, we observed that the cytochrome (*Cyt*) *b_6_* expression is increased in the *pgr5* mutant to regulate proton motive force and ATPase across the thylakoid membrane. The increased Cyt *b_6_* function in *pgr5* could be due to the compromised function of chloroplast (cp) ATP synthase subunits for energy generation and photoprotection under high light. Moreover, our proteome data show that the photosystem subunit II (PSBS) protein isoforms (PSBS1 and PSBS2) expressed more than the Light-Harvesting Complex Stress-Related (LHCSR) protein in *pgr5* compared to WT and *pgrl1* under high light. The immunoblot data shows the photosystem II proteins D1 and D2 accumulated more in *pgrl1* and *pgr5* than WT under high light. In high light, CP43 and CP47 showed a reduced amount in *pgr5* under high light due to changes in chlorophyll and carotenoid content around the PSII protein, which coordinates as a cofactor for efficient energy transfer from the light-harvesting antenna to the photosystem core. BN-PAGE and circular dichroism studies indicate changes in macromolecular assembly and thylakoid super-complexes destacking in *pgrl1* and *pgr5* due to changes in the pigment–protein complexes under high light. Based on this study, we emphasize that this is an excellent aid in understanding the role of CET mutants in thylakoid protein abundances and super-complex organization under high light.

## Introduction

Light is a predominant need for photosynthesis, and its quantity can influence photosynthetic efficiency. Optimum photosynthesis requires a subtle balance between photoreception and utilization of ideal light intensity and a constant supply of nutrients. Photosynthetic organisms that grow under natural conditions do not always receive the optimum light and nutrients, leading to photosynthetic imbalance. Photosynthetic organisms have been adapted to various photoprotective mechanisms and changes in core and antenna protein to acclimate under abiotic stress conditions. High light is particularly problematic because excess light affects the charge separation and damages the pigment–protein complexes involved in photosynthetic electron transport, which causes an imbalance in the reducing powers of NADPH and ATP for carbon fixation and photoacclimation. Absorption of light energy and its conversion into chemical form are carried out by the coordinated function of specialized thylakoid membrane protein complexes like photosystems (PS) I and PSII along with their light-harvesting (LHC) antenna LHC I and II, the cytochrome (Cyt) *b_6_f*, and the ATP–synthase complex. These protein complexes are well-organized as super-complexes in the appressed sections of the thylakoid membrane (Shen et al., 2019; Wietrzynski et al., 2020). In *Chlamydomonas (C.) reinhardtii*, these super-complexes consist of PSII–LHCII, PSI–LHCI, LHC trimers, monomers, and other small subunits like CP26 and CP29 (Croce and van Amerongen, 2020).

The antenna proteins associate with reaction centers (RCs), allowing overexcitation of energy from the membrane until it drives stable photochemistry (Bennett et al., 2018). PSII has been modified to minimize ROS generation due to the RC complex strategy, redox tuning (Rutherford et al., 2012; Brinkert et al., 2016), and supporting mechanism dissipating excess energy and inhibiting the damage of photosystems (Allakhverdiev et al., 2008; Peers et al., 2009; Roach et al., 2020). In photosynthetic organisms, photoinactivation of PSII is rapidly repaired and reflects the balance between the light-induced damage to PSII and the repair of PSII (Allakhverdiev and Murata, 2004). Photosynthetic organisms have evolved with different agencies to dissipate extra energy absorbed by photosynthetic machinery. These mechanisms are collectively called non-photochemical quenching (NPQ). Energy quenching, qE, is mediated by the light harvesting complex stress related 3 (LHCSR3) antenna after the protonation of its lumen residues (Peers et al., 2009; Tian et al., 2019). In green algae, *C. reinhardtii*, the induction of qE requires gene products of LHCSR, i.e., LHCSR1, LHCSR3.1/3.2, and PSBS (Peers et al., 2009; Correa-Galvis et al., 2016). The PSBS protein does not form a complex with pigments (Bonente et al., 2008). More likely, its function depends on the protonation of the luminal-exposed glutamine residues, which can further activate the zeaxanthin and lutein-dependent quenching process in LHCs through chlorophyll–carotenoid charge transfer (Niyogi et al., 2001; Ahn et al., 2008; Bonente et al., 2008). PSI is particularly resistant to photoinhibition under oxidative stress conditions due to the high efficiency of protective mechanisms, which regulate the flow of electrons to the PSI donor side, including NPQ, lumen pH-dependent regulation of Cyt *b_6_f* activity, and even PSII photoinhibition (Tikkanen and Aro, 2014). Electron consumption at the PSI acceptor side through the Calvin–Benson cycle, photorespiration, and cyclic and pseudo-cyclic electron flow are also protective factors that avoid PSI over-reduction (Yamori, 2016). In algae, the cyclic electron transport is operated by *pgrl1* and *pgr5* proteins, but the role of these proteins in high light photoprotection and the metabolic process is still unknown.

Proteomics must be an essential match to biochemical and biophysical data to improve the biological perspective on plant biology. This approach is applied to discover the dynamic changes in protein expression to a specific abiotic stress in various organisms. Several studies have investigated protein expression using a perspective method called whole proteome analysis. This technique can determine the relative levels of peptides in abiotic stress and normal physiological condition. The difference or similarity in expression levels under different light conditions can be revealed by differential expression in the peptide levels of specific sets of proteins. The impact or influence of high light on a particular biological process can be reported by changes in the proteome levels of a specific protein that has a functional role in the performance of photosynthesis. A detailed knowledge of biological pathways and processes is required to determine the reasons for microalgae acclimation and biomass production under high light. Hence, a new straightforward proteome analysis platform is necessary to elucidate these advantages and identify potential targets for higher plants to improve crop production.

Proteomics is a promising approach that could deliver insights into and knowledge of the protein, which helps improve and utilize conventional breeding and genetic engineering to make new traits or recombinant plants with better-quality agronomical characteristics (Gao et al., 2019). Notably, several efforts have been accomplished to achieve gene expression in alfalfa in salt conditions (Jin et al., 2010; Postnikova et al., 2013). High-throughput techniques have rapidly been discovered and helped us better understand plants’ transcriptome and proteome changes during light and dark (Vogel et al., 2014; Liang et al., 2016; Crisp et al., 2017; Huang et al., 2019). The present study characterized a bio-physiological, biochemical study integrated with nLCMS/MS-based proteomic analysis to quantify the global database resource by the eukaryotic microalgae *C. reinhardtii*. It also determined how *C. reinhardtii* globally allocates resources in response to high light stress.

The applied strategies can quantitatively and temporally account for all apparent behaviors, including changes in physiological characteristics, biochemical parameters, and whole-cell proteome content change in WT, *pgrl1*, and *pgr5* mutants. The cyclic electron transport *pgrl1* and *pgr5* proteins are extensively involved in energy generation, which is utilized for metabolic processes. These mutants showed reduced photosynthetic activity and non-photochemical quenching (Yadav et al., 2020; Chouhan et al., 2023). Recently, our findings showed that more biomass and lipid production are fundamental mechanisms of photoprotection in response to light stress (Chouhan et al., 2022). We have grown *C. reinhardtii* cells in Tris-acetate phosphate (TAP) to address these questions in high light conditions. No reports have been documented in the proteomic responses of microalgae *C. reinhardtii* when the cells were grown under high light intensities, mimicking natural conditions. The present study examined the effect of high light at different light intensities on green microalga *C. reinhardtii* through proteomic analysis combined with a biochemical study.

## Materials and methods

### Growth condition

*C. reinhardtii* wild-type strain 1*37c* and mutants *pgrl1* and *pgr5* are a gift from Prof. Gilles Peltier, CEA-CNRS-Aix Marseille Université, France, and Prof. Michael Hippler, University of Munster. The cells were grown in a TAP medium in an Algaetron growth chamber (AG 230-ECO, Czech Republic) with white fluorescent LED light shaken at 120 rpm (Yadav et al., 2020). The WT, *pgrl1*, and *pgr5* cells were grown in 50 µmol photons m^−2^s^−1^ (optimal growth), moderate (250 µmol photons m^−2^s^−1^), and high light (500 µmol photons m^−2^s^−1^). The chlorophyll content was analyzed using 80% acetone. We have used three or four biological replicates for all the experiments.

### Measurement of photosynthetic O_2_ evolution and respiratory O_2_ consumption

The light-saturated oxygen evolution and consumption rate (under ~600 µmol photons m^−2^ s^−1^) were measured from 2-ml samples of intact cells with a Clark-type oxygen electrode (Hansatech Instruments Ltd, Norfolk, United Kingdom) at 25°C in a TAP medium. 20 µl of the artificial electron acceptor 2,5-dichloro1,4-benzoquinone (DCBQ, 0.5 mM) and 20 µl of 100 mM NH_4_Cl were injected into the electrode reaction. The cell mixture was kept in the dark for 2 min, and then the light was turned on (or O_2_ evolution rate measurement) for 6-min continuous light illumination (600 µmol photons m^−2^ s^−1^) until the straight bend line no longer appeared. Respiratory oxygen consumption was measured by adding 100 mM sodium azide, 50 mM methyl viologen, 10 mM DCPIC, 50 mM DCMU, and 500 mM ascorbate and the cell is kept in the dark until the chamber oxygen is not entirely consumed. The oxygen electrode was calibrated with air-saturated water (100% oxygen) by adding sodium dithionite to achieve 0% oxygen. The final chlorophyll concentration used for each replicate was 20 μg ml^−1^. For better activity, cells were mixed with buffer 5 mM Tris-HCl (pH 7.5), 10 M MgCl_2_, 5 mM CaCl_2_, 30 mM KCl, and 0.25 M sorbitol (Nakalai, Japan). Experiments were the average of three biological replicates.

### Room temperature fluorescence

Room temperature spectra of chlorophyll fluorescence were recorded by a fluorescence spectrophotometer (Perkin Elmer, LS-55). 10 µg chlorophyll concentration was taken for each measurement. Fluorescence spectra were recorded from 800 to 600 nm, exciting at 435 nm with a scan speed of 100 nm/min. Each spectrum is an accumulation of two scans.

### Measurement of circular dichroism under high light

The circular dichroism (CD) spectra of thylakoid membranes isolated from control and high light-treated cells were measured by a J-810 spectropolarimeter (Jasco Inc., Easton, MD, USA). The spectra were recorded within a visible wavelength range (400–800 nm) by a quartz cell with an optical path length of 1 cm and three bandwidths. The sample thylakoid was dissolved into a thylakoid suspension buffer of 20 mM Tris (pH 7.5), 0.3 M sorbitol, 10 mM MgCl_2_, 10 mM NaF, and 5 mM CaCl_2_ with a concentration of 20 μg/ml. The three scans were repeated with a 100-nm/min continuous scan speed for each replicate. Three independent experiments were taken for the measurement.

### Blue native PAGE analysis of high light-treated thylakoid membranes

The thylakoid membranes were isolated from cells grown in high light dissolved in thylakoid resuspension buffer containing 0.2 M sorbitol, 5 mM Tris–HCl (pH 7.5), and 5 mM CaCl_2_. Blue native PAGE was performed with a 4% stacking and 4%–12.5% gradient resolving gel polymerized from a 32/1 bis-acrylamide/acrylamide mixture (Järvi et al., 2011). Thylakoid was isolated from the control and the light-treated sample containing 1 mM PMSF, 1 mM ACA, and 1 mM benzamidine hydrochloride as a protease inhibitor. A total of 10 μg of Chl was loaded per lane, with a final Chl concentration of 0.5 mg ml^−1^ and a final detergent concentration of 1% β-DM. Native protein markers were from GE Healthcare (UK).

### Gel electrophoresis and immunoblotting

The proteins from cells were separated under denaturing conditions on SDS-PAGE. The cells were grown in optimal growth light and high light conditions, and cells were centrifuged, and the pellet was stored in 20% glycerol. Cells were solubilized into a 2× sample buffer containing 1 M Tris-HCl (pH 6.8), 10% 1 mM DTT, 100% glycerol, and 10% SDS slowly mixed with pipetting. The sample was heated for 1 min at 100°C and then centrifuged at 10,000 rpm for 10 min to collect the supernatant and loaded the sample on an equal Chl basis. SDS-PAGE was performed using an SDS-PAGE system with a 4% stacking and 12% or 15% resolving gel. Three different quantities [0.25 (25%) or 0.5 (50%) and 1.0 (100%) μg] of Chl were loaded for each lane to compare the quantification of the protein as reported earlier (Devadasu and Subramanyam, 2021). The nitrocellulose membrane was incubated with primary antibodies (LHCII, I, PSII, and PSI) raised in rabbit and the antibody dilutions were as follows: For the PSII–LHCII complex: PsbA and PsbB (1:5,000); CP47 (1:2,000); CP43 (AS04038; 1:10,000); Lhcb1 (AS01004; 1:10,000); LhcB2 (1:5,000); Lhcb4 (1:10,000); Lhcb5 (1:10,000); PsbO (1:5,000); For the PSI–LHCI complex: PsaA, PsaD, PsaH, PsaG, and PsaF (1:10,000) (all antibodies were purchased from Agrisera, Sweden). The LHCI antibodies followed the following dilutions: Lhca1 (1:5,000); Lhca2 (1:5,000); Lhca3 (1:5,000); Lhca4 (1:3,000); Lhca5 (1:3,000); Lhca6 (1:3,000); Lhca7 (1:2,500); and Lhca9 (1:3,500) (these antibodies were raised in our laboratory). Subsequently, secondary antibody (HRP-conjugated anti-rabbit, 1:20,000 dilution; Agrisera) embellishment was performed for 1 h at RT at 10 rpm. Chemiluminescence signal detection was performed using ECL Western Blotting solution (Bio-Rad) and the ChemiDoc Imaging System (Bio-Rad).

### Whole proteome analysis of *C. reinhardtii* cells under high light stress: protein extraction and quantification

*C. reinhardtii* control and light-treated cells were grown and harvested at 3,000 rpm for 5 min. Cell pellets were finely mixed in 10% glycerol, dipped in liquid nitrogen, and stored at −80°C for further analysis. The cell pellet was taken into a 15-ml Falcon tube (Genaxy, India) and suspended in 4 ml of extraction buffer containing 0.5 M Tris-HCl (pH 7.5), 0.7 M sucrose, 0.1 M KCl, 50 mM EDTA, 2% β-mercaptoethanol, and 1 mM PMSF. After thoroughly mixing, Tris-saturated phenol (pH 7.5) was added to the extracted suspension. The whole suspension was mixed for 30 min at 4°C in a rotor spin cyclomixer. Tris-saturated phenol was prepared by mixing an equal volume of Tris-HCl (pH 7.5) and phenol with continuous stirring for 3–4 h. The lower phenolic layer was separated, and an equal volume of Tris-HCl (pH 7.5) was added with constant stirring for 2–3 h. The lower phenolic layer was collected and stored in an amber glass bottle at 4°C. The sample mixture was centrifuged at 5,000×*g* for 30 min at 4°C. The upper phenolic phase was collected carefully, and an equal volume of extraction buffer was added. This step was performed repeatedly, and the phenolic stage was re-extracted. Four volumes of ice-cold 0.1 M ammonium bicarbonate in methanol were added to the final collected phenolic phase and incubated overnight at −20°C for protein precipitation. The next day, the samples were centrifuged at 10,000×*g* for 30 min at 4°C. The pellet was washed thrice with ice-cold methanol, twice with acetone, and air-dried for a few minutes. The final pellet was dissolved in 200 μl of the rehydration solution containing 8 M urea, 2 M thiourea, 30 mM DTT, 4% CHAPS, and 0.8% IPG buffer of pH range 4–7 (GE Healthcare). The protein concentration was determined using Bradford reagent (Bio-Rad) with BSA as standard (standard curve of 0–100 mg ml^−1^ concentration).

### nLC-MS/MS analysis

100 µg of the final pellet was treated with 100 mM DTT for 1 h at 95°C, followed by 250 mM iminodiacetic acid (IDA) for 45 min at room temperature in the dark. The sample suspension was incubated with trypsin at 37°C for overnight digestion. The trypsin-digested peptides were extracted in 0.1% formic acid solution at 37°C for 45 min. The solution was centrifuged at 10,000×*g*, and the supernatant was collected in the fresh tube for vacuum drying. The final sample was solubilized in 20 µl of 0.1% formic acid. For the separation of peptides, 10 µl of injection volume was loaded on the C18 UPLC column, and peptides were separated by Waters Synapt G2 Q-TOF for MS and MS/MS analysis. For LC-MS analysis, 10 µl of the sample was injected into the ACQUITY UPLC system (Waters, UK) equipped with an ACQUITY UPLC BEH C18 column (Waters, UK) (150 mm × 2.1 mm × 1.7 µm), a SYNAPT G2 QTOF (Waters, UK), and an electrospray ionization (ESI) source. The sample analysis was run on the positive mode by applying 3,500 V capillary voltage and 30 L of cone gas flow per hour. The source and desolvation glass flow were maintained at 1.8 and 800 L/h, and the temperatures of source and desolvation were 150°C and 350°C, respectively. The protein range was from 50 Da to 150 Da. The trap and the transfer collision energy were continuously maintained at 6 V. The ramp collision energy was set at 20 V and increased up to 45 V. The total acquisition time was 60 min, and the solution flow rate was 300 nl/min. The mobile phase consisted of 0.1% formic acid in water (solvent A) and 0.1% formic acid in acetonitrile (solvent B). A linear 60-min gradient consisted of solvent A 98% and solvent B 2% for 1 min, solvent A and B 50% for 29 min, solvent A 20% and solvent B 80% for 15 min, followed by 15 min solvent A 98% and solvent B 2%. A wash solution was used at the end of each program to reduce carryover between samples.

### Protein identification

The raw data acquired from the above analysis were processed using PLGS software 3.0.2 (Waters, India; identification and expression algorithm), within which data processing and database search was performed. The samples—*C. reinhardtii* proteins—for two sample sequences in FASTA format were downloaded from Swiss-Prot and used to search peptides present in samples. On each run, the sample was processed using the following search parameters in the software: peptide tolerance, 50 ppm; fragment tolerance, 100 ppm; minimum number of fragment matches for peptide, 2; minimum number of fragment matches for proteins, 5; and carbamidomethylating of cysteine and oxidation of methionine were selected as fixed and variable modifications, respectively. UniProt (*C. reinhardtii*, reviewed protein) was used as the database against which the search was done.

### Gene ontology and bioinformatics analysis

This study’s identified protein species were annotated based on their molecular function, biological process, and cellular component with Gene Ontology (GO) annotation using UniProt. Hierarchical cluster analysis was performed using the R statistical package based on correlation values. Network analysis was performed by using Cytoscape bioinformatics software version (3.7.2).

### Statistical analyses

The experiments conducted in the current study were carried out with at least three biological replications (*n* = 3). The R statistical package constructed hierarchical cluster matrices and protein–protein networks based on correlation values and *p*-values less than 0.05.

## Results

### High light changes oxygen evolution/consumption in *pgrl1* and *pgr5*

**Figure 1:**
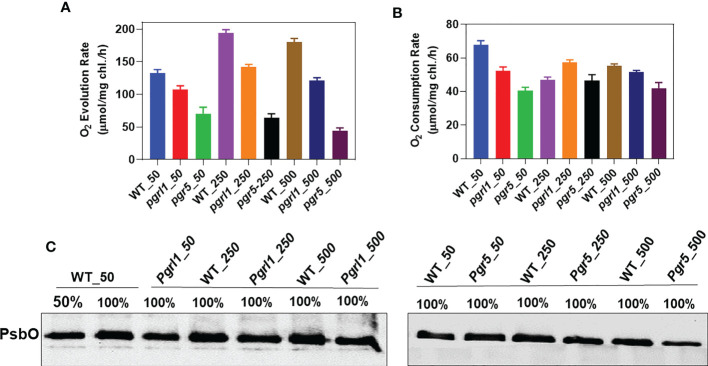
Measurement of oxygen evolution/consumption. Comparison of oxygen evolution and consumption rates of the wild type (WT), and knockdown strains, (*pgrl1* and *pgr5*). Cells were grown in different light conditions. The oxygen-evolving activity of cells was measured by taking an equal concentration of chlorophyll, 10 μg/ml, supplemented with the artificial electron acceptor 1 mM DCBQ and 1 mM NH_4_Cl for PSII activity **(A)**. Oxygen evolution recordings were measured at 25°C under saturating light (600 µmol photons m^−2^s^−1^) using a Clark-type oxygen microelectrode (Hansateck Instrument Ltd, Norfolk, England) stuck inside the oxygen electrode chamber. Oxygen consumption measurements were carried out with cells incubated in a TAP medium containing NaN_3_, ascorbate, and DCPIP at a final concentration of 1 mM **(B)**. In high light, the oxygen-evolving complex subunit of PsbO decreased in *pgrl1* and *pgr5* **(C)**. The electrode was calibrated in a medium flushed with air (O_2_ saturated) in the presence of sodium hydrosulfite (anoxia). Standard deviations were estimated from three biological replicates.

Factually, oxygen evolution, spectrometry, and biochemical measurements were used to quantify PSII damage under abiotic stress (Chow and Aro, 2005; Tyystjärvi, 2013). The oxygen evolution results demonstrate that the WT cells acclimate when grown under high light. Their oxygen-evolving capacity is increased by approximately 47% (194 μmol/mg Chl/h) in moderate light and 36% (180.33 μmol/mg Chl/h) in high light (**Figure 1A**). A significant decrease of 51.58% and 66.35% in oxygen evolution in *pgr5* has been observed in moderate and high light, respectively, than in WT growth light conditions. On the other hand, in *pgrl1*, a minor decrease is observed in high light conditions. The Chl fluorescence has mainly decreased in *pgrl1* and *pgr5* in moderate and high light than in WT growth light conditions. When cells were dark-adapted, the WT and *pgrl1* observed an almost similar O_2_ consumption under high light (**Figure 1B**). There is a significant decrease in dark-induced O_2_ consumption in moderate and high light, corresponding to nearly 15% and 24% in *pgrl1* and 32% and 38% in *pgr5* compared to WT growth light conditions. Furthermore, we correlate this result with biochemical changes; the oxygen-evolving complex subunit of PsbO slightly decreased in *pgrl1* and *pgr5* in moderate and high light than WT (**Figure 1C**), as shown in the O_2_ uptake experiment (**Supplementary Figures 1A–C**). These results propose that WT cells adjust to high light stress by adaptative mechanism, but *pgrl1* and *pgr5* drastically damaged PSII, which agrees with the fluorescence result (**Supplementary Figure 2A**).

### Structural changes in thylakoid membrane under high light

**Figure 2:**
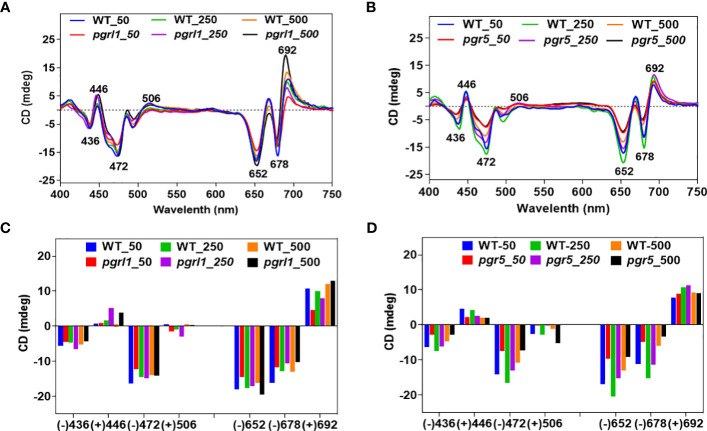
CD spectra of wild-type and mutants **(A, B)**. Spectra were recorded at room temperature, baseline-corrected, and normalized to equal chlorophyll (10 µg/ml). The total amplitude of the psi-type signal [i.e., the difference between the positive and negative amplitudes is shown as a bar **(C, D)** diagram] of WT, *pgrl1*, and *pgr5* cells grown under growth light and high light conditions, which received the same parameter and three accumulations with the scan speed of 100 from three independent experiments, on different batches. Values represent the mean and three replicates.

Thylakoid membrane structure and composition were analyzed using CD spectrometry and BN-PAGE. The thylakoid membrane is a dynamic structure; it can undergo numerous structural changes to acclimate under different environmental conditions (Nagy et al., 2014). CD determines the structural change of the macro-organization of super-complexes and pigment–pigment interactions. The WT and mutant CD spectra are presented in **Figures 2A, B**. The CD spectra of the thylakoid membrane are divided into the Qy region (600 to 700 nm or towards the red region) and the Soret region (400 to 550 nm or blue region). The CD spectra of the thylakoid membrane revealed the features called *psi*-type CD bands. The magnitude of the *psi*-type band is proportional to the size of the ordered array at a given set of conditions.

The visible region of the CD spectrum of *C. reinhardtii* cells consists of two intense *psi*-type bands at approximately (−) 676 and (+) 690 nm (Nagy et al., 2014). The positive band at approximately 690 nm (Chl *a*) and the negative band at approximately 674 nm (Chl *b*) originated from Chl molecules associated with macrodomains of photosynthetic proteins. These *psi*-type bands arose due to the presence of large well-ordered arrangements (size ranging from 200 to >400 nm) of PSII–LHCII complexes in the appressed region of the thylakoid membrane. In WT, there are marginal changes in the red psi-type at approximately (+) 692-nm signal amplitude, especially in moderate compared to high light. In contrast, we detected a significant increase in the *psi*-type signal from *pgrl1* and *pgr5* grown under high light compared to WT growth light (**Figure 2A**). On the other hand, another *psi-*type band at approximately (−) 678 is significantly decreased in *pgrl1* and *pgr5* in moderate and high light compared to WT control. This indicates that *pgrl1* and *pgr5* are crucial for maintaining the thylakoid dynamics and structural arrangements under high light.

The blue (Soret) region *psi*-type band at (+) 506 nm is attributed to carotenoids found in a long-range ordered structure, and the (−) 650-nm negative band originated from LHCII trimers (Garab and van Amerongen, 2009). Also, as reported earlier, the Soret region 506 nm originated from the β-carotene of the core complex (Tóth et al., 2016). Our results show that this peak is decreased drastically, especially in *pgr5*, which might be due to a decrease in the protein content of the core complex in cells subjected to high light (**Figure 2B**). The CD spectra of LHCII trimers are similar under normal and high light but slightly increased in moderate and high light in WT. At the same time, *pgrl1* LHCII trimers are increased, but in *pgr5*, peak intensity is drastically reduced compared to WT control, suggesting that the LHCII trimers were significantly affected in CET mutants under high light.

The spectra have a more complex structure in the Soret region (400 to 450 nm) because of an abundance of Chl *a* (+) 436 nm and Chl *b* (+) 446 nm, and the dominant peak at (−) 472 nm for LHCII. There is no such difference in the peak intensity of (−) 436 nm in WT and *pgrl1* in moderate and high light, and it decreased in *pgrl1* and *pgr5* in high light. On the other hand, the peak intensity of (+) 446 nm increased in *pgrl1* in moderate and high light but reduced significantly in *pgr5* in high light compared to WT control (**Figures 2A, B**).

The LHCII band shows a (−) 472-nm similar rise in peak WT and *pgrl1* in moderate and high light but decreased dramatically under *pgr5* high light conditions as shown in **Figures 2C, D**. It is clear that though there is acclimation, the LHCs to RC assembly changed, describing that the LHCs are detached from the RC. The data agree with blue native gels that disturbed super-complexes and mega-complexes. However, these disturbed mega- and super-complexes were restored as the monomer and trimer content significantly reduced in *pgr5*. 

### Protein pigment interaction and changes in macro-organization of super-complexes

**Figure 3:**
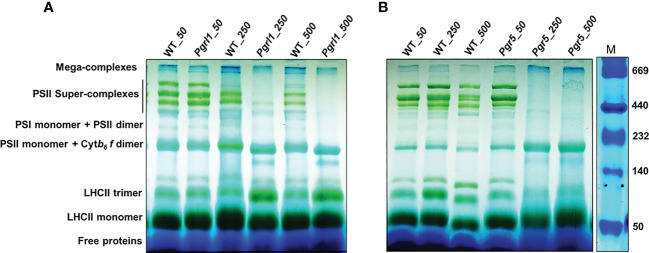
Pigment–protein complexes and super-complexes were seperated using BN-PAGE gel. Thylakoid membranes from WT, *pgrl1*, and *pgr5* samples were solubilized with 1% β-DM and separated using native PAGE. WT, *pgrl1*, and *pgr5* containing equal amounts of chlorophyll (10 μg) were loaded in each lane shown in **(A, B)** panel with a Native marker.

Thylakoid membranes comprise several multiprotein membrane complexes that run photosynthetic light reactions. Their composition, distribution, and stoichiometry may vary according to environmental conditions. Thylakoids include three structures: grana, stroma, and grana margins. The appressed regions of thylakoids, grana, are enriched with PSII–LHCII super-complexes, non-appressed parts, and stroma with PSI–LHCI and ATPase complexes (Garab and van Amerongen, 2009). Grana margins have been suggested to have both PS I and II form mega-complexes (Järvi et al., 2011). **Figures 3A, B** show typical patterns of thylakoid membrane protein complexes separated in BN-PAGE. Four bands of PSII–LHCII super-complexes between 669 and 440 kDa, PSII and PSI monomers, and Cyt *b_6_f* complex devoid of LHCs, near 440–230 kDa, and the lower three bands consisting of different hierarchal combinations of LHC trimer and monomer have been resolved from the thylakoids under high light. The localization of the Cyt *b_6_f* complex is under debate, but many studies reported its occurrence near PSII dimer (Anderson, 1982; Albertsson et al., 1991). The largest PSII super-complexes isolated from *C. reinhardtii* are classified as a strongly bound trimer (S-trimer), a moderately bound trimer (M-trimer), and a loosely bound trimer (L-trimer) depending on their binding strength with the PSII core complex as C_2_S_2_M_2_L_2_. From BN-PAGE, the four high-molecular-weight bands as a super-complex (~669 kDa) consist of different combinations of PSII core with S and M trimers, and the largest super-complex observed is C_2_S_2_M_2_.

The WT under moderate and high light stress shows a marginal decrease of PSII–LHCII super-complex (**Figure 3A**). However, in *pgrl*1, the PSII–LHCII super-complexes decrease even more severely in *pgr5* under moderate and high light (**Figures 3A, B**). The reduction of PSII–LHCII super-complexes reflects in the form of LHCII trimer and monomer. In WT and *pgr5*, the fraction of LHCs is stored as a monomer than *pgrl1*; trimer is restored more under moderate and high light conditions (**Figure 3A**). The loosely bound LHCII complexes in mega-complexes and super-complexes (top bands) were dissociated and further accumulated as LHCII trimers. These changes are less noticeable in WT and mutants under high light.

### Western blot analysis of proteins from *C. reinhardtii* WT and *pgrl1* and *pgr5* mutants

**Figure 4:**
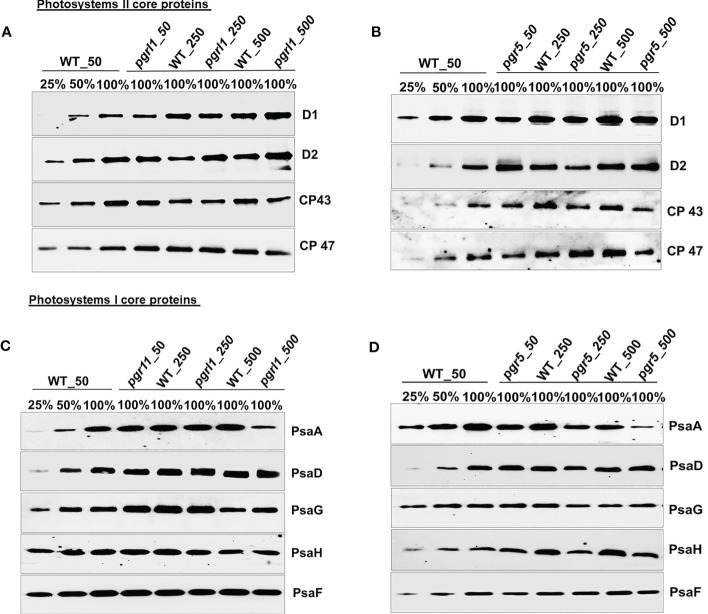
Immunoblot analysis of PSII **(A, B)** and PSI **(C, D)** core proteins selected from wild-type (WT) and *pgrl1* and *pgr5* mutants. The WT and *pgrl1* and *pgr5* mutants were grown under constant growth light (50 μmol photons m^−2^ s^−1^) and moderate (250 μmol photons m^−2^ s^−1^) and high light (500 μmol photons m^−2^ s^−1^) conditions. The Western blots shown here are representative of at least three biological replicates.

Immunoblot analysis was performed on WT, *pgrl1*, and *pgr5* grown in optimal (50 µmol photons m^−2^ s^−1^), moderate (250 µmol photons m^−2^ s^−1^), and high light (500 µmol photons m^−2^ s^−1^) regimes. We have identified the proteins of PSII (D1, D2, CP43, and CP47) and PSI (PsaA, PsaD, PsaG, PsaH, and PsaF) core proteins along with both photosystems (PSII and PSI) and LHCs (light-harvesting complex) by Western blot. The changes in the stromal and luminal subunits (PsaG, PsaH, and PsaD) can distract the assembly of PSI. The proteins representing PSII core PsbA (D1) and PsbD (D2) accumulated more in *pgrl1* and *pgr5* than in WT under high light stress. However, D1 and D2 did not differ significantly in *pgr5* grown under moderate light compared to the WT control condition (**Figures 4A, B**). The PSII major antenna protein CP43 is decreased noticeably in *pgr5* while slightly decreased in *pgrl1* than WT control under moderate and high light. However, another antenna protein, CP47, significantly reduced in high light in *pgr5* but increased dramatically in *pgrl1* and *pgr5* in moderate light than WT control. The PsbA (D1) protein content slightly increased in *pgrl1* and *pgr5* in high light than WT control (**Figure 4A**). In high light stress conditions, CP43 and CP47 showed a reduced amount in *pgr5* due to changes in chlorophyll and carotenoid molecules around the PSII protein, which coordinates the cofactors to transfer electrons to PSI (**Figure 4B**).

The PGRL1 and PGR5 proteins are part of the PSI complexes and are extensively involved in CET for energy generation. The absence of this protein affects the culture growth and other photosynthetic parameters related to both PSI and PSII in high light. Additionally, we checked the outcome of high light on *pgrl1* and *pgr5* strains, and how the absence of these proteins affects other PSI proteins under high light is studied. The PsaA subunit of the PSI showed a more than 50% decrease under moderate light while a 75% decrease in *pgr5* under high light (**Figure 4D**). However, PsaA protein content was unaffected in *pgrl1* under moderate light but significantly decreased in high light compared to WT control. At the same time, there was a marginal decrease of PsaD and PsaG protein content in *pgrl1* and WT under high light, but these proteins are unaffected under moderate light (**Figure 4C**). The stromal subunits PsaC and PsaD act as a binding site for ferredoxin, which leads to an efficient electron transport chain (Fischer et al., 1998). *pgrl1* the PsaD is marginally decreased in moderate light but increased slightly in *pgr5* under high light. At the same time, PsaG accumulates in WT and *pgr5* under moderate and high light.

On the other hand, another subunit PsaD is noticeably accumulated in WT and *pgr5* under moderate and high light than WT. Together, mutants confirmed that the protein content of PsaF did not change from WT under moderate and high light conditions, showing that the arbitrary presence in the PSI complexes was unaffected (**Figures 4C, D**). Parallel results have been described earlier in *A. thaliana pgr5* and *pgrl1* (DalCorso et al., 2008). The immunoblot analyses of the LHCII proteins were relatively stable in WT and *pgrl1* compared to *pgr5* under high light.

**Figure 5:**
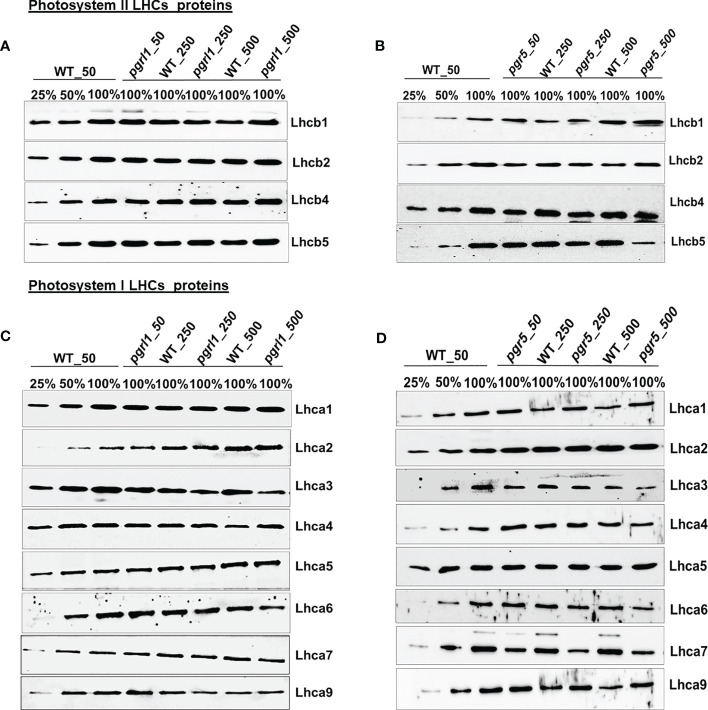
Western blotting analysis of all the LHCs of photosystem II and I subunit. Immunoblot analysis of the selected light-harvesting complex protein of PSII **(A, B)** and PSI **(C, D)** from wild-type (WT) and *pgrl1* and *pgr5* mutants that were grown under constant growth light (50 μmol photons m^−2^ s^−1^) and moderate (250 μmol photons m^−2^ s^−1^) and high light (500 μmol photons m^−2^ s^−1^) conditions. The Western blots shown here are representative of at least three biological replicates.

The Lhcb1 protein increased marginally in *pgrl1* and *pgr5* in high light, while the mutation did not affect the accumulation of Lhcb2 in *pgrl1* but slightly reduced in *pgr5* in high light compared to WT control (**Figures 5A, B**). The minor light-harvesting complexes Lhcb4 (CP29) and Lhcb5 (CP26) have been proposed to play a crucial role in the zeaxanthin-dependent (qZ) high light-induced regulation of NPQ in *C. reinhardtii*. The Lhcb4 and Lhcb5 proteins are almost unaffected in WT but increased in *pgrl1* under moderate and high light than in WT (**Figure 5A**). However, in *pgr5*, the Lhcb4 protein is decreased slightly but significantly in moderate and high light compared to WT, indicating that *pgr5* was affected more than *pgrl1* (**Figure 5B**).

Similarly, we can see that high light-induced photoprotective protein LHCSR3 accumulated more in WT and *pgrl1* but is absent in *pgr5* under moderate and high light. Furthermore, we examined the protein content of the light-harvesting complex of PSI that is arranged in two layers towards the stromal side of the PSI core and is essential for efficient energy transfer and PSI photoprotection. The four PSI light-harvesting proteins, Lhca1, Lhca8, Lhca7, and Lhca3, are situated in the internal layer, and another four, Lhca1, Lhca4, Lhca6, and Lhca5, are positioned in the outside layer towards the PSAF, and the two subunits, Lhca2 and Lhca9, associated with PSAB core (Ozawa et al., 2018). Interestingly, the light-harvesting complex of PSI—Lhca1, Lhca4, Lhca5, and Lhca7—is relatively stable in *pgrl1*, whereas Lhca2, Lhca3, Lhca6, and Lhca9 are extensively affected in *pgrl1* and *pgr5* under high light (**Figures 5C, D**).

Unusually, the association of Lhca1, Lhca7, Lhca8, and Lhca3 is more steady than Lhca4, Lhca5, and Lhca6 light-harvesting proteins, which are positioned in the innermost layer (Drop et al., 2011). The Lhca1 is almost unaffected in *pgrl1* and *pgr5* in moderate and high light compared to the WT control (**Figures 5C, D**). The Lhca2 and Lhca9 present near the PsaG subunit increased in *pgrl1* and *pgr5*, except that the Lhca9 in *pgrl1* decreased under moderate and high light WT. The location of lhca3 close to PSAK is consistent with previously reported results (Moseley et al., 2002). The Lhca3 is present in the outer side of the edge in the inner ring, and Lhca6 at the outer ring is reduced significantly in *pgrl1* and *pgr5* under moderate and high light compared to WT control. Two other LHCs, Lhca4 and Lhca5, are likely located in the outer ring of PSI polypeptide and unaffected under moderate and high light in *pgrl1* and *pgr5* compared to WT (**Figures 5C, D**). The accumulation of the inner subunit of Lhca7 is unchanged in *pgrl1*, whereas it decreased significantly in *pgr5* under moderate and high light than WT.

### High light changes protein coverage and up-/downregulated and differentially expressed proteins between treated and control samples

The current study combined the *n*LC-MS/MS technique and targeted proteome to unravel the essential proteins accountable for resistance to high light from *C. reinhardtii*. We would like to understand the importance of the CET mutants photosynthetic electron transport chain (PETC) proteins and downstream metabolism in *C. reinhardtii*. Total cell proteomes were taken from cultures grown under optimal growth and high light conditions to address the following question (**Supplementary Figure 3A**). A Venn diagram of each protein was established and observed almost 295 proteins in WT, 306 in *pgrl1*, and 299 in *pgr5* under growth light while 306 in WT, 306 in *pgrl1*, and 286 in *pgr5* in high light. We have placed almost 249 DEPs in WT_50 and 284 DEPs in high light stress from the entire proteome. Of the total, 284 proteins showed common expression in growth light WT_50 and high light W_500 (**Supplementary Figure 3B**). Seventeen DEPs in WT_50 while 52 DEPs in WT_500 showed unique expression under low and high light conditions.

**Figure 6:**
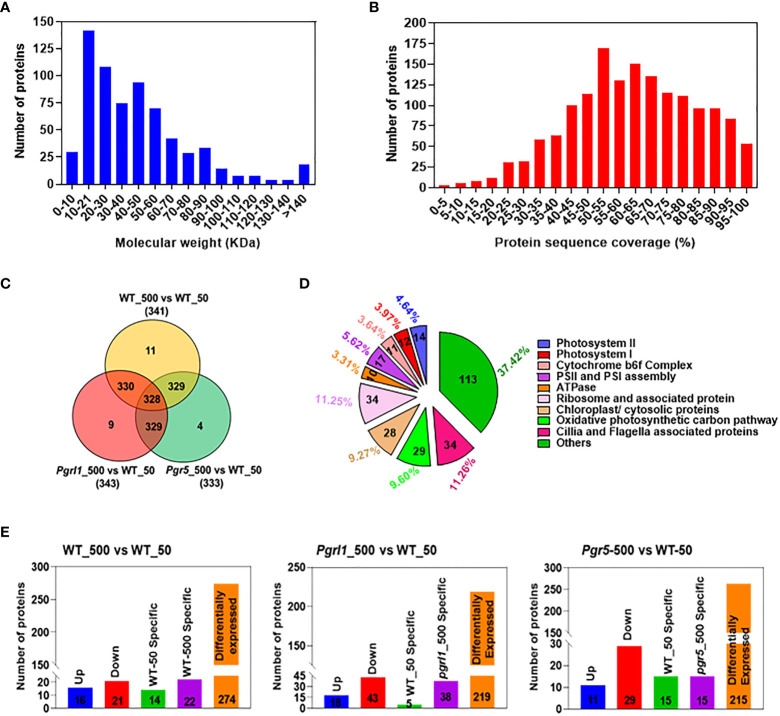
Whole-cell proteome analysis between treated and control cells. Total numbers of proteins against different molecular weights **(A)**. The number of proteins against additional protein sequence coverages **(B)**. Venn diagrams showing the significantly different protein levels in light treated vs. control cells **(C)**. The total number of proteins that share unique expression levels is represented as a percentage **(D)**. The comparative proteome of the total number of upregulated, downregulated, and differentially expressed proteins (DEPs) **(E)** under growth and high light conditions.

Furthermore, we have annotated and enriched the *C. reinhardtii* proteome with the sample-identified peptide after nLC-MS/MS analysis categorized them based on molecular weight and protein sequence coverage (**Figures 6A, B**). We obtained 6,654 peptides in WT under control light conditions (denoted as WT_50, here 50 µmol photons m^−2^ s^−1^ is light intensity in growth light) and 14,822 peptides under high light-treated (WT_500, here 500 µmol photons m^−2^ s^−1^ is light intensity at high light) conditions. The *pgrl1* showed 23,133 peptides under growth light (*pgrl1_*50) and 20,515 peptides under high light (*pgrl1_*500) conditions. On the other hand, *pgr5* showed 12,733 peptides under growth light (*pgr5*_50) but only 4,362 peptides under high light (*pgr5*_500) conditions. Furthermore, we enriched all peptides into proteins, which share common and exclusively expressed proteins in treated versus control samples, as shown in **Figure 6C**. Overall, 302 proteins from several functional groups were significantly affected together in treated and control samples, out of which 14 (4.64%) were associated with PSII, 12 (3.97%) were involved in PSI, 11 (3.64%) were associated with Cyt *b_6_f* complex, 10 (3.31%) were engaged in ATPase assembly, and 17 (5.62%) were involved in photosystem (PSI and PSII) assembly.

There were 64 (22%) proteins associated with photosynthesis, 29 (9.60%) proteins related to mitochondria, 28 (9.27%) proteins related to chloroplast/cytosol, 34 (11.25%) proteins related to translational machinery, 34 (11.26%) proteins associated with Cilia/Flageller assembly, and 113 (37.42%) proteins associated with primary and secondary metabolism (**Figure 6D**). These up- and downregulated proteins are primarily engaged in photosynthesis, metabolic pathway, glycolysis, protein synthesis, cilia, flagella-associated proteins, and proteins involved in cytoskeleton assembly.

We have also compared *Chlamydomonas* proteome-treated versus control samples to understand them much better. In WT_500 vs. WT_50 (**Supplementary Table 1**), we identified 274 as DEPs; 16 proteins are upregulated, 21 are downregulated, and 14 and 22 proteins are exclusively expressed in WT_50 and WT_500, respectively (**Figure 6E**). In *pgrl1*_500 vs. WT_50 (**Supplementary Table 2**), we identified 219 as DEPs; 18 proteins are upregulated, 43 are downregulated, and 5 and 38 proteins are exclusively expressed in WT_50 and *pgrl1*_500, respectively. In *pgr5*_500 vs. W_50 (**Supplementary Table 3**), we have identified 215 as DEPs; 40 proteins are upregulated, 12 are downregulated, and 21 and 14 proteins are exclusively expressed in WT_50 and *pgr5*_500, respectively (**Figure 6E**).

### Changes in photosynthetic and high light-inducible proteins of *C. reinhardtii* under high light

**Figure 7:**
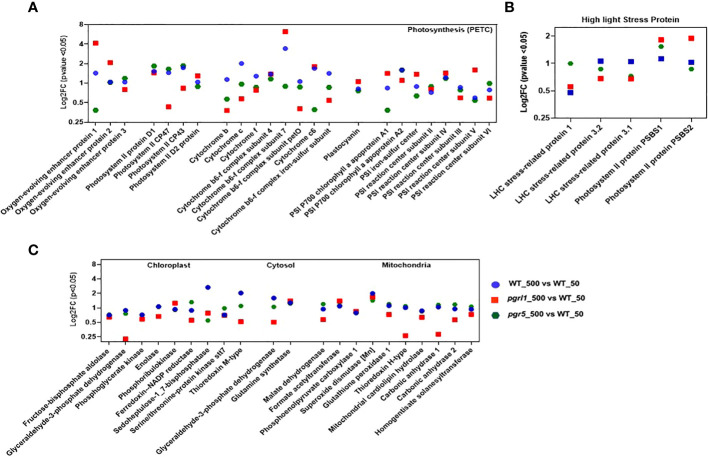
Proteins participating in photosynthetic machinery (PETC: photosynthetic electron transport chain) involved in OEC, PSII core, *Cyt* b_6_f complex, and PSI core **(A)** proteins are significantly affected under high light stress and are shown as log2FC (*n* = 3). Proteins expressed in high light stress belonging to NPQ generation **(B)** are mainly involved under high light. Proteins participating in the chloroplast, cytosol, and mitochondria **(C)** that are involved considerably under high light are shown. Proteins with fold change and *p*-value < 0.05 are shown (*n* = 3).

DEPs contributing to photosynthesis were defined under high light. The water-oxidizing complex, PSBO_P12853 (Oxygen-evolving enhancer protein 1_chloroplastic) and PSBP_P11471 (Oxygen-evolving enhancer protein 2_chloroplastic), are upregulating (≥2FC) in *pgrl1*_500 vs. WT_50 and *pgr5*_500, and PsbQ_P12852 (Oxygen-evolving enhancer protein 3_chloroplastic) was differentially regulated in all the variants (**Figures 7A**). Only water-oxidizing protein PSBO_P12853 is downregulated (≤0.5FC) in *pgr5* (*pgr5*_500 vs. WT_50) under high light.

The PSII RC proteins PSBA_P07753 (D1 protein), PSBD_P06007 (D2 protein), and PSBC_P10898 (PS II CP43 RC protein) are differentially regulated (≤2FC and ≥0.5FC) in WT, *pgrl1*, and *pgr5* under high light compared to growth light. On the other hand, the major antenna proteins PSBB_P37255 (PS II CP47 reaction center protein) are downregulated in *pgrl1* (*pgrl1*_500 vs. WT_50) under high light compared to growth light (**Figure 7A**).

Three PSI RC proteins, PSAB_P09144 (PSI P700 chlorophyll an apoprotein A2), PSAE_P12352 (PSI reaction center subunit III chloroplastic), and PSAF_P12356 (PSI reaction center subunit III_chloroplastic), have a more than twofold increase in expression in WT (WT_500 vs. WT_50) under high light. The two PSI core proteins with a downregulated < 0.5FC expression in *pgr5* are PSAA_P12154 (PSI P700 chlorophyll, an apoprotein A1) and PSAG_P14224 (PS I reaction center subunit V_chloroplastic), as they decrease in protein blot under high light conditions. Other PSI proteins like PSAC_Q00914 (PSI iron-sulfur center), PSAD_Q39615 (PSI reaction center subunit II_chloroplastic), and PSAH_P13352 (PSI reaction center subunit VI_chloroplastic) are differentially regulated (≤2FC and ≥0.5FC) under WT-, *pgrl1-*, and *pgr5*-treated vs. control light conditions (**Figure 7A**). The possible role of PSAH_P13352 could be provided with the docking site of the LHC I antenna protein to the core complex.

The CYC_P15451 was more downregulated in *pgr5*_500 vs. WT_50 (~one-third of log2FC). The log2FC expression of CYF_P23577 (*p* < 0.05) did not significantly changed in *pgrl1* and *pgr5*. Another essential Cyt complex protein is CYB6_Q00471 (Cyt b_6_), whose log2FC expression is increased 281 times in *pgr5* (*pgr5*_500 vs. *pgr5*_50) compared to WT high light (WT_500 vs. WT_50, **Figure 7A**). The PETD_P23230 (Cyt complex subunit 4), another type of Cyt complex protein, was similarly expressed in WT, *pgrl1*, and *pgr5* in all high light conditions, except that a slight downregulation of PETD_P23230 was observed in *pgrl1* (*pgrl1*_500 vs. *pgrl1*_50) under the HL condition (**Figure 7A**). PETM_Q42496 (Cyt *b_6_f* complex subunit 7_chloroplastic) and PETO_Q9LLC6 (Cyt *b_6_f* complex subunit 5_chloroplastic) have been differentially expressed (≤2FC and ≥ 0.5FC) in *pgrl1* and *pgr5*, except for the PETM_Q42496, Cyt *b_6_f* subunit encoded by the nucleus, which is apparently effective in the signaling pathway (Schneider et al., 2001) and whose expression is upregulated (≥2FC) in *pgrl1* (*pgrl1*_500 vs. WT_50) and WT (WT_500 vs. *pgr5*_50 and WT_500 vs. W_50) under high light. Similar results have been described earlier in *A. thaliana pgr5* and *pgrl1* (DalCorso et al., 2008).

The ATP synthase subunit ATPA_P26526 (ATP synthase subunit alpha_chloroplastic), ATPB_P06541 (ATP synthase subunit beta_chloroplastic), and ATBG_P12113 (ATP synthase gamma chain_chloroplastic) are essential for proton uptake from the lumen to balance the proton conductivity on the thylakoid membrane and are differentially regulated in *pgrl1* (WT_500 vs. *pgrl1*_50) under high light (**Supplementary Figure 4A**). The ATPD_Q42687 (ATP synthase delta chain_chloroplastic) and ATPE_P07891 (ATP synthase epsilon chain_chloroplastic) are upregulated in WT (WT_500 vs. WT_50), but ATPD_Q42687 is increased exceptionally 64 times in *pgr5* (*pgr5*_500 vs. WT_50), showing the importance of CET for energy generation. However, proteins ATPD_Q42687 and ATPE_P07891 are downregulated in *pgrl1* (*pgrl1*_500 vs. W_50) compared to WT, indicating the significance of CET under high light stress. *pgrl1* and *pgr5* substantially reduced CYF_P23577 (Cyt f) and CYC_P15451 (Cytc) of the Cyt *b_6_f* complex compared to WT under high light.

The stress-related proteins LHCSR3 and PSBS were differently expressed (≤2FC and ≥0.5FC) in *pgrl1* and *pgr5* under high light. The LHC stress-related protein 1 (LHSR1_P93664) is mainly involved in zeaxanthin-dependent quenching and increased more than >2FC expression in *pgr5* (*pgr5*_500 vs. WT_50) than WT (WT_500 vs. WT_50) under high light. The LHC stress-related protein 3.1 (LHSR3.1_P0DO19) and LHCSR3.2_P0DO19, induced by lumen acidification, were decreased (log2FC expression) in *pgrl1* and *pgr5* mutants compared to WT in high light. The LHCSR3.2_P0DO19 has increased log2FC expression under WT high light (WT_500 vs. WT_50) compared to under WT control conditions (**Figure 7B**). However, *pgrl1* has decreased log2FC expression of LHSR3.2_P0DO19, whereas its expression was lacking in *pgr5* under high light conditions. This finding shows that *pgr5* can generate the least NPQ, while *pgrl1* has a smaller NPQ than WT (Yadav et al., 2020).

Another high light-expressing protein, PSBS, is present in two isoforms, the first one being PSBS1_A8HPM2 (PSII protein PSBS1), whose log2FC expression is higher in *pgr5* (*pgr5*_500 vs. WT_50) and *pgrl1* (*pgrl1*_500 vs. WT_50) than in WT under high light. Conversely, in PSBS2_A8HPM5 (PSII protein PSBS2), the log2FC expression increased mainly into *pgrl1* (*pgrl1*_500 vs. WT_50) under high light. The data show that the LHCSR3.1_P0DO19 and LHCSR3.2_P0DO19 are mainly expressed proteins in WT. In contrast, PSBS2_A8HPM5 and PSBS1_A8HPM2 are significantly expressed in *pgr5* and *pgrl1* and is the major effector protein under high light photoprotection in *C. reinhardtii* (**Figure 7B**). The PSBR_A0A2K3DMP5 (PSII protein PSBR chloroplastic) protein is essential for the steady binding of LHCSR3 to PSII–LHCII and is crucial for the efficient quenching and for maintaining the stability of the PSII–LHCII–LHCSR3 super-complex under high light.

### High light changes the metabolism of chloroplast, cytosol, and mitochondria

*C. reinhardtii* is a mixotroph that can act as an autotroph or heterotroph, depending on medium conditions. The control light-grown cultures have a robust carbon fixation potential, but downstream metabolites were changed when grown in high light, where the growth was affected. The RubisCO subunit RUBA_Q42694 (RuBisCO large subunit-binding protein subunit alpha_chloroplastic) and RUBB_Q42693 (RuBisCO large subunit-binding protein subunit beta-1), a chaperone protein involved in RubisCO assembly, were deferentially regulated in WT (WT_500 vs. WT_50), but RUBA_Q42694 is downregulated in *pgrl1* (*pgrl1*_500 vs. WT_50) and RUBB_Q42693 in *pgr5* (*pgr5*-500 vs. W-50) under high light. Another subunit of RuBisCO assembly, RUBC_Q42695 (RuBisCO large subunit-binding protein subunit beta-2), binds RuBisCO’s small and large subunits, is occupied in the enzyme assembly, and is differentially regulated in WT and *pgrl1* but downregulated in *pgr5* (*pgr5*_500 vs. W_50) under high light conditions (**Supplementary Figure 4B**).

The glycolytic enzyme ALFC_Q42690 (Fructose-bisphosphate aldolase 1_chloroplastic) is an essential enzyme in the energy pathways of algae; it was differentially regulated (≤2FC and ≥0.5FC) in WT, *pgrl1*, and *pgr5* under high light (**Figure 7C**). The enzyme G3PA_P50362 (Glyceraldehyde-3-phosphate dehydrogenase A_chloroplastic) involved in the glucose metabolic process was downregulated in *pgrl1* (*pgrl1*_500 vs. WT_50) under high light. Similarly, it is differentially regulated in WT (WT_500 vs. WT_50) under high light. Simultaneously, the protein KPPR_P19824 (Phosphoribulokinase_chloroplastic) is associated with the Calvin cycle; part of carbohydrate biosynthesis was differentially regulated in *pgrl1* and *pgr5* (*pgrl1*_500 vs. WT_50) and *pgr5* (*pgr5*_500 vs. WT_50) under high light.

The enzyme FENR_P53991 (Ferredoxin-NADP reductase_chloroplastic) is vital for adjusting the cyclic and non-cyclic electron transport to meet the requirement of ATP, and reducing power was differentially regulated in WT (WT_500 vs. WT_50) and *pgrl1* (*pgrl1*_500 vs. WT_50). TRXM_P23400 (Thioredoxin M-type_chloroplastic) is a vital regulatory player receiving information about the redox state of the PETC and participates in the reversible oxidation of the active center dithiol to a disulfide, which was highly upregulated by more than threefold (>3 log2FC) in WT (WT_500 vs. WT_50) and was differentially regulated in *pgrl1* (*pgrl1*_500 vs. W_50) and *pgr5* (*pgr5*_500 vs. WT_50) under high light.

The M type of TRX is known to activate NADP-malate dehydrogenase to hold the CET indirectly. The enzyme ENO_P31683 (Enolase) was differentially regulated in WT (WT_500 vs. WT_50), *pgrl1* (*pgrl1*_500 vs. WT_50), and *pgr5* (*pgr5*_500 vs. WT_50) under high light. The key enzyme STT7_Q84V18 (Serine/threonine-protein kinase stt7_chloroplastic) is required for state transition by phosphorylating the LHC II external antenna differentially regulated (≤2FC and ≥0.5FC) in WT, *pgrl1*, and *pgr5* in high light (**Figure 7C**). The S17P_P46284 (Sedoheptulose-1,7-bisphosphatase) chloroplastic protein is highly upregulated (more than >2FC) in WT (WT_500 vs. WT_50) and was differentially regulated in *pgrl1* (*pgrl1*_500 vs. W_50) and *pgr5* (*pgr5*_500 vs. WT_50) in high light.

Comparing the whole cell proteome of *C. reinhardtii*, high light-grown cultures suggested that metabolic enzyme was strongly affected in *pgrl1* and *pgr5*. The tricarboxylic acid (TCA) cycle of MDHM_Q42686 (Malate dehydrogenase_mitochondrial) was differentially regulated in WT (WT_500 vs. WT_50), *pgrl1* (*pgrl1*_500 vs. WT_50), and *pgr5* (*pgr5*_500 vs. WT_50) under high light (**Figure 7C**). MDHM_Q42686, a mitochondria enzyme, synthesizes extra reducing power for chloroplast by converting oxaloacetate to malate by a malate shuttle shunt. This way, an additional NADPH pool is synthesized and regenerated (Scheibe, 2004).

The mitochondrial protein GPX1_P83564 (Glutathione peroxidase 1) constitutes a glutathione peroxidase-like protective system against oxidative stresses, synthesizing PUFA through arachidonic acid metabolic pathways, and was differentially regulated in *pgrl1* and *pgr5* in high light. The protein PAPP1_P81831 (phosphoenolpyruvate carboxylase 1) is involved in the carboxylation of phosphoenolpyruvate (PEP), forms oxaloacetate, and was differentially regulated (≤2FC and ≥0.5FC) in WT, *pgrl1*, and *pgr5* in high light. This enzyme is activated by glutamine and dihydroxyacetone phosphate and inhibited by glutamate and malate.

Superoxide dismutase (Mn), mitochondrial (SODA), destroys superoxide anion radicals within the cells. It is toxic to biological systems with more than twofold change (>2FC) upregulated in *pgrl1* (*pgrl1*_500 vs. W_50) and more than the threefold differentially (>3FC) upregulated in *pgr5* (*pgr5*_500 vs. WT_50) under high light, although its expression was flexible and statistically significant (*p* < 0.05), whereas TRXH_P80028 (thioredoxin H-type) protein contributes to several redox responses through the reversible oxidation of the dithiol into disulfide by taking the electron from ferredoxin, with the activity of enzyme FTR (ferredoxin: thioredoxin reductase) differentially regulated (≤2FC and ≥0.5FC) in WT, *pgrl1*, and *pgr5* and its H form is known to activate several cytosolic enzymes (**Figure 7C**).

CAH1_P20507 and CAH2_P24258 (Carbonic anhydrase 1 and 2) are present in the mitochondria and responsible for the reversible carbonate dehydratase activity because the formation of CO_2_ was downregulated (<0.2 log2FC) in *pgrl1* (*pgrl1*_500 vs. WT_50) under high light. The metabolic protein homogentisate solanesyltransferase (HSTC_A1JHN0) not only is involved in PQ synthesis but also participates in carotenoids, and abscisic acid (ABA) biosynthesis pathways were differentially regulated (≤2FC and ≥0.5FC) in WT, *pgrl1*, and *pgr5* under high light.

### Heatmap representing the comparative expression of the differentially abundant proteins

**Figure 8:**
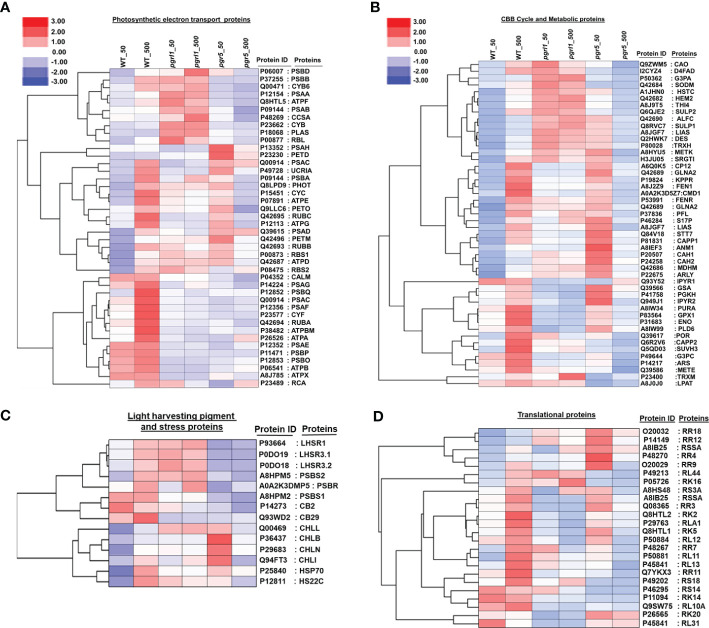
Global protein expression levels in *C reinhardtii* in response to high light stress. Hierarchical clustering heatmap representing the relative expression of the DAPs between light-treated versus control cells. Hierarchical clustering based on the abundant levels of protein identified in photosynthesis electron transport **(A)**, CBB and metabolic proteins **(B)**, light-harvesting pigment and stress proteins **(C)**, and translational proteins **(D)** was performed using Gene Cluster 3.0 software with the similarity metric and linkage method. The resulting clusters were visualized using Java Tree View software.

To elucidate the heatmap analysis, we employed the expression value of WT, *pgrl1*, and *pgr5*. Most of the proteins were upregulated in WT, and few were in the *pgrl1*, but the majority of proteins are downregulated in *pgr5* in high light conditions. The heatmap study categorized the identified DEPs into four distinctive categories: photosynthetic electron transport protein, Calvin cycle and metabolism protein, light-harvesting pigment, photoprotection proteins, and protein involved in translational machinery. The photosynthetic electron transport proteins were divided into 42 sub-categories with three major clusters: PS II and PS I protein subunits, Cyt *b_6_f* complex, ATP synthase, RuBisCO subunits, and electron carrier proteins (**Figure 8A**).

During the light reaction of the photosynthesis, we identified 10 DEPs from Cluster 1 [PSBB_P37255, PSBD_P06007, PSAA_P12154, PSAB_P09144, CYB6_Q00471, CYB_P23662 (*Cyt* b), ATPF_P12356, CCSA_P48269 (*Cyt* c biogenesis protein CcsA), RBL_P00877 (Ribulose bisphosphate carboxylase large chain), and PLAS_P18068 (Plastocyanin chloroplastic)], in which most of the proteins are in *pgrl1*, but few of them (PSBB_P37255, ATPF_P12356, and CYB6_Q00471) in WT are upregulated compared to *pgr5* in high light. Cluster 2 includes 17 proteins [PSBA_P07753, PSBC_P10898, PSAH_P13352, PETD_P23230, UCRIA, PHOT_Q8LPD9 (Phototropin), CYC_P15451, PETO_Q9LLC6, ATPE_P07891, RUBC_Q42695, PSAD_Q39615, ATPG_P12113, ATPD_Q42687, PETM_Q42496, RUBB_Q42693, RBS1_P00873 (Ribulose bisphosphate carboxylase small chain 1_chloroplastic), and RBS2_P08475 (Ribulose bisphosphate carboxylase small chain 2_chloroplastic)] that are upregulated in WT high light (**Figure 8A**). However, 13 proteins upregulated in *pgr5* were associated with PSII chlorophyll-binding protein D1, PSI protein, ATP synthase subunit, Rubisco subunit, *Cyt* c subunit, and phototropin protein in growth light conditions.

Cluster 3 includes 15 proteins [CALM_P04352 (Calmodulin), PSAG_P14224, PSBQ_P12852, PSAF_P12356, PSAC_Q00914, CYF_P23577, RUBA_Q42694, PSAE_P12352, PSBO_P12853, PSBP_P11471, ATPBM_P38482, ATPX_A8J785 (ATP synthase subunit b’_chloroplastic), ATPA_P26526, ATPB_P06541, and RCA_P23489 (Ribulose bisphosphate carboxylase/oxygenase activase_chloroplastic)] exclusively involved in oxygen-evolving proteins, Cyt f protein, and ATP synthase subunit upregulated in WT high light conditions.

A total of 250 DEPs were divided into 49 sub-categories of the Calvin cycle and metabolic proteins into three significant clusters. Cluster 1 includes CAO_Q9ZWM5 (Chlorophyllide a oxygenase_chloroplastic), D4FAD_I2CYZ4 (Acyl-lipid (7-3)-desaturase_chloroplastic), G3PA_P50362, SODM_Q42684 (Superoxide dismutase (Mn)_mitochondrial), HSTC_A1JHN0 (Homogentisate solanesyltransferase_chloroplastic), HEM2_Q42682 (Delta-aminolaevulinic acid dehydratase_chloroplastic), THI4_A8J9T5 (Thiamine thiazole synthase_chloroplastic), SULP1_Q8RVC7, SULP2_Q6QJE2 (Sulfate permease 1 and 2_chloroplastic), ALFC_Q42690, LISC_A8I2V9 (Lipoyl synthase_chloroplastic), DES_Q2HWK7 (Acyl-lipid omega-13 desaturase), TRXH_P80028 (), SRGT1_H3JU05 (Peptidyl serine alpha-galactosyltransferase), METK_A8HYU5 (S-adenosylmethionine synthase), and 15 DEPs with the maximum number observed in the dark reaction and are upregulated in *pgrl1* in growth and high light conditions (**Figure 8B**).

Cluster 2 includes CP12_A6Q0K5 (Calvin cycle protein CP12_chloroplastic), GLNA2_Q42689 (Glutamine synthetase_chloroplastic), KPPR_P19824 (Phosphoribulokinase_chloroplastic), FEN1_A8J2Z9 (Flap endonuclease 1), CMD1_A0A2K3D5Z7 (5-methylcytosine-modifying enzyme), FENR_P53991, GLNA1_Q42688 (Glutamine synthetase cytosolic isozyme), PFL_P37836 (Formate acetyltransferase), S17P_P46284, LIAS_A8JGF7 (Lipoyl synthase_mitochondrial), STT7_Q84V18 (Serine/threonine-protein kinase stt7_chloroplastic), CAPP1_P81831 (Phosphoenolpyruvate carboxylase 1), and ANM1_A8IEF3 (Protein arginine N-methyltransferase 1). It also contains CAH1_(P20507), CAH2_(P24258), MDHM_Q42686 (Malate dehydrogenase_mitochondrial), ARLY_P22675 (Argininosuccinate lyase), and 17 DEPs in which 7 are upregulated in WT high light and 15 are upregulated in *pgr5* growth light compared to *pgrl1*. These proteins are involved in carbon fixation, protein lipoylation, modifiable cyclic, and non-cyclic electron transport, supplying additional ATP and reducing power and CO_2_ uptake.

Cluster 3 includes TRXM_P23400 (Thioredoxin M-type_chloroplastic), G3PC_P49644 (Glyceraldehyde-3-phosphate dehydrogenase_cytosolic), GPX1_P83564 (Glutathione peroxidase 1_mitochondrial), GSA_Q39566 (Glutamate-1-semialdehyde 2_1-aminomutase_chloroplastic), IPYR1_Q93Y52 (Soluble inorganic pyrophosphatase 1_chloroplastic), PURA_A8IW34 (Adenyl succinate synthetase_chloroplastic), POR_Q39617 (Protochlorophyllide reductase_chloroplastic), PGKH_P41758 (Phosphoglycerate kinase_chloroplastic), CAPP2_Q6R2V6 (Phosphoenolpyruvate carboxylase 2), ARS_P14217 (Arylsulfatase), ENO_P31683 (Enolase), IPYR2_Q949J1 (Solubleinorganicpyrophosphatase2), METE_Q39586 (5-methyl tetra hydropteroyl triglutamate-homocysteine methyltransferase), SUVH3_Q5QD03 (histone-lysine N-methyltransferase_H3 lysine-9 specific SUVH3), PLD6_A8IW99 (Mitochondrial cardiolipin hydrolase), LPAT_A8J0J0 (1-acyl-sn-glycerol-3-phosphate acyltransferase), and 16 DEPs that commonly upregulate in WT high light. Five proteins are exclusively upregulated in *pgr5* growth light and are involved in the glycolytic process (G3PC_P49644, PGKH_P41758, GSA_Q39566, and ENO_P31683), purine nucleotide biosynthesis (PURA_A8IW34), light-independent chlorophyll biosynthesis (POR_Q39617), and lipid biosynthesis (LPAT_A8J0J0) (**Figure 8B**). The light-harvesting pigments and photoprotection proteins were subdivided into 14 sub-categories with two major clusters.

Cluster 1 includes LHR3.1_P0DO18, LHSR1_P93664, LHSR3.2_P0DO19, PSBS1_A8HPM2, PSBS2_A8HPM5, PSBR_A0A2K3DMP5, CB29_Q93WD2, and CB2_P14273; two photoprotective proteins (LHCSR and PSBS); and two Chl a/b binding proteins (CB29 and CB2) upregulated in WT and *pgrl1* but downregulated in *pgr5* high light (**Figure 8C**). We have found that Cluster 2 includes CHLB_P37824 (Light-independent protochlorophyllide reductase subunit B), CHLL_Q00469 (Light-independent protochlorophyllide reductase iron-sulfur ATP-binding protein), CHLN_P29683 (Light-independent protochlorophyllide reductase subunit N), CHLI_Q94FT3 (Magnesium-chelatase subunit ChlI_chloroplastic), HSP70_P25840 (Heat shock 70 kDa protein), HS22C_P12811 (Heat shock 22 kDa protein_chloroplastic), and H2A_P50567 (Histone protein), and seven DEPs associated with chlorophyll pigment-binding protein upregulated in WT high light. 

The protein involved in translational machinery was further subdivided into 24 sub-categories with two clusters. Among them, minor clusters containing five DEPs [RR18_O20032, RR4_P48270, RR12_P14149 (30S ribosomal protein S7_chloroplastic), RSSA_A8IB25 (40S ribosomal protein SA), and RL44_P49213 (60S ribosomal protein L44)] were upregulated in *pgr5* growth light. In contrast, significant clusters included 19 DEPs [RR2A_O47027, RR3_Q08365, RR7_P48267, RR9_O20029, RR11_P14149 (30S ribosomal proteins_chloroplastic), RS3A_A8HS48, RS14_P46295, RS18_P49202 (40S ribosomal protein), RK2_Q8HTL2, RK5_Q8HTL1, RK14_P11094, RK16_P05726, RK20_P26565 (50S ribosomal proteins_chloroplastic), RLA1_P29763, RL10A_Q9SW75, RL11_P50881, RL12_P50884, RL13_O48513, and RL31_P45841 (60S ribosomal protein L13)] in which 17 were upregulated in WT and the remaining 2 (RK20_P26565 and RL31_P45841) were upregulated in *pgr5* high light condition (**Figure 8D**).

### Gene Ontology and Kyoto Encyclopedia of Genes and Genomes analysis

**Figure 9:**
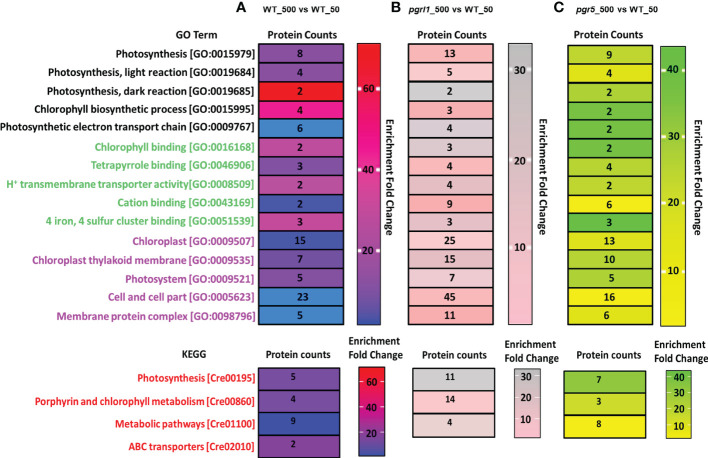
Gene Ontology (GO) and Kyoto Encyclopedia of Genes and Genomes (KEGG) analysis on light stress-induced up-/downregulation of expressed proteins is divided into three categories of Go Term, including GO function and ID, Protein count, and Enrichment fold change. Panels **(A–C)** represent the up-/downregulation of proteins induced by WT-500 vs. W-50 **(A)**, *pgrl1*-500 vs. W-50 **(B)**, and *pgr5*-500 vs. W-50 **(C)** treatment versus control. BP1 -5, biological process; MF1 -5, molecular function; CC1 -5, cellular components The top three biological pathways were presented as KEGG pathways as follows for upregulated, downregulated, and differentially regulated proteins: (1) BP1 (biological process), Photosynthesis; BP2, Photosynthesis, light reaction; BP3, Photosynthesis, dark reaction; BP4, chlorophyll biosynthetic process; BP5, photosynthetic electron transport chain; (2) MF1 (molecular function), chlorophyll-binding; MF2, tetrapyrrole binding; MF3, H^+^ ion transmembrane transporter activity; MF4, cation binding; MF5, four iron, four sulfur cluster binding; (3) CC1-5 (cellular components)—chloroplast, chloroplast thylakoid membrane, photosystems, cell and cell part, and membrane protein complex. However, the KEGG pathway represents the top three pathways in differentially abundant proteins involved in photosynthesis, porphyrin and chlorophyll metabolism, and metabolic pathways.

The Gene Ontology (GO) enrichment analysis includes a biological process (BP), cellular component (CC), and molecular function (MF) of upregulated, downregulated, and DEPs (p-value is <0.05) under high light stress. Gene ontology analysis in WT_500 vs. WT_50 high light proteomics reveals a special enrichment for PSII. We detected considerable changes in proteins growing in high light WT samples. Out of 350 DEPs, 18 were involved in 18 BP, 15 proteins in 14 CC, and 23 were engaged in 35 MF categories. We have also separated some core proteins from the groups WT_500 vs. WT_50, mainly involved in ETR, CET, and water photolysis. KEGG pathway showed that 16 DEPs are primarily interested in photosynthesis and metabolic pathway proteins (**Figure 9A**). GO enrichment analysis of the global proteomic dataset of lightly treated *pgrl1*_500 vs. WT_50 cultures revealed that *pgrl1* was enriched over WT in which 39 proteins were involved in 83 biological functions, 32 types of proteins in 88 MF, and 45 types of proteins in 58 CC, including protein, unfolding, PSII repairing, and oxidative responses related to ROS.

KEGG pathway showed that 15 DEPs are mainly involved in three major categories, i.e., photosynthesis, oxidative phosphorylation, and metabolic pathway (**Figure 9B**). Quantitative proteomics was performed on *pgr5_500* vs. WT_50 under high light conditions, where 294 proteins were quantified in all functional categories, in which 15 proteins were involved in 15 different biological functions, 11 proteins in 12 MF, and 16 proteins in 31 CC categories. In contrast, *pgr5* lacked GO terms related to carbohydrate metabolism, TCA cycle, and PSII electron transport (**Figure 9C**). KEGG pathway showed that nine DEPs are mainly engaged in three major categories, i.e., photosynthesis, oxidative phosphorylation, and metabolic pathway. These proteins show differential protein expression in high light and play an essential role in photoprotection. In contrast, *pgr5* proteins remarkably decrease in high light, including assembly and stabilization of PSII, OEC complex assembly, and oxygen evolution, which is crucial for the regulation of photosynthetic-related protein that has not been reported yet in algae so far. Notably, few photosynthesis-related proteins were not detected in WT high light.

### Volcano plot and protein network analysis of DEP-regulated protein under high light stress

**Figure 10:**
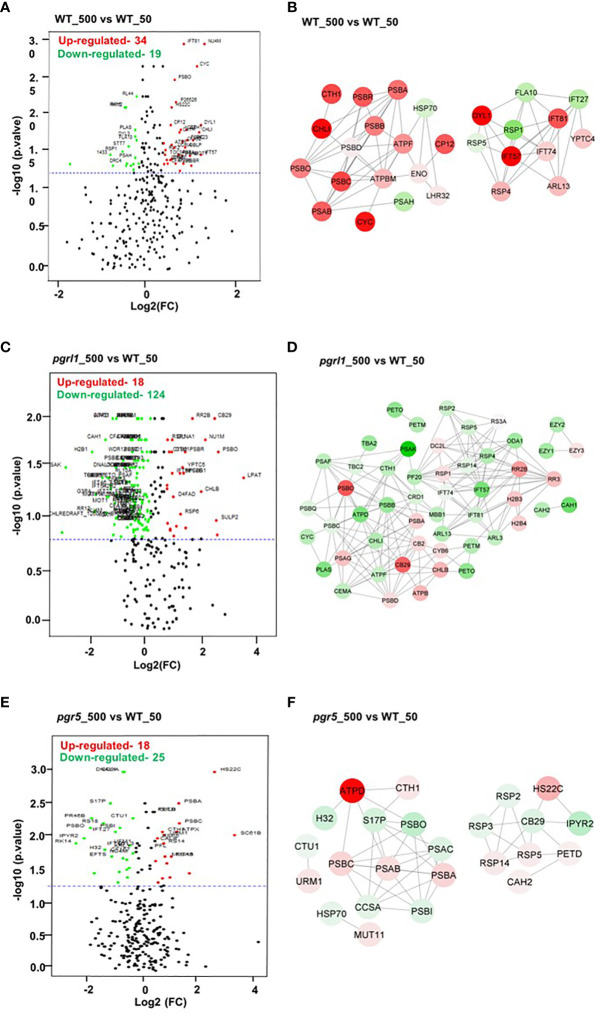
Volcano plot and correlation network analysis of DEPs in WT, *pgrl1*, and *pgr5* mutants. Volcano plots **(A, C, E)** and protein–protein interaction networks **(B, D, F)** represent the proteins with significant differences induced by WT-500 vs. WT-50, *pgrl1*-500 vs. WT-50, and *pgr5*-500 vs. WT-50. Proteins with significant differences and large fold changes are shown in red scatters as upregulated and green as downregulated. *X*- and *Y*-axis indicate a considerable difference in terms of both the two-sided Student’s *t*-test (*p*-value < 0.05) and the fold cutoff (>1.2 and <0.86).

Volcano plots represent the proteins with substantial alterations made by high light in WT, *pgrl1*, and *pgr5*. Following light treatment, 274 DEPs significantly changed in WT_500 vs. WT_50, with 34 upregulated and 19 downregulated proteins (**Figure 10A**), while 219 DEPs significantly changed in the *pgrl1* treated vs. control samples (*pgrl1*_500 vs. WT_50), with 18 upregulated and 124 downregulated (**Figure 10C**). Comparing the *pgr5*_500 vs. WT_50, a total of 215 DEPs are significantly identified, of which 18 are upregulated and 25 are downregulated after light treatment (**Figure 10E**). Based on the volcano plot, we have taken the DEPs and elucidated the protein–protein interaction network of selected fold values of ≤0.86 (downregulated—low abundant protein) and ≥1.2 (upregulated—high abundant protein) in treated vs. control samples in WT_50 vs. WT_50, *pgrl1*_500 vs. WT_50 and *pgr5*_500 vs. WT_50. The nodes were shown in circles with two colors (red—upregulated protein, green—downregulated) to simplify further the correlation network based on the degree of interactions.

For the WT_500 vs. WT_50 DEPs correlation network, every data point was recognized as a node, and a total of 28 nodes (each node represents a single protein) formed 76 edges (neighboring interactions); on average, each node shared two to three edges with adjacent nodes (**Figure 10B**). The red nodes belonging to proteins associated with PETC PSII (PSBA_P07753, PSBB_P37255, PSBC_P10898, and PSBD_P06007), OEC (PSBO_P12853), PSI (PsaA_P12154), ATP synthase (ATPF_P12356 and ATPBM_P38482), high light-induced protein (LHSR3.2_P0DO19), and proteins involved in interflagellar assembly [IFT57_Q2XQY7, IFT81_Q68RJ5, and IFT74_Q6RCE1 (intraflagellar transport protein)] are upregulated.

The green nodes belonging to heat shock protein HSP70_P25840, PSI PSAH_P13352, L-serine amino-acid biosynthesis PSP1_A8JEM3 (O-phosphoserine phosphohydrolase**),** and protein involved in interflagellar assembly (IFT27_A8HN58 (Intraflagellar transport protein 27) are downregulated (**Figure 10B**). To simplify the *pgrl1*_500 vs. WT_50 correlation network, the 52 nodes were categorized in circles by two colors (red and green) based on the degree of interactions. Green nodes comprise a dense network containing 172 interaction edges with neighboring nodes. Interestingly, the green nodes belonging to proteins related to PETC (PSBB_P37255, PSBC_P10898, PSBQ_P12852, PSAF_P12356, and PSAK_P14225), plastocyanin (PLAS_P18068), ATP synthase subunits (ATPD_Q42687 and ATPF_P12356), Cyt *b_6_f* subunit for CET (PETO_Q9LLC6 and PETM_Q42496) interflagellar assembly (IFT81_Q68RJ5, IFT57_Q2XQY7, and IFT74_Q6RCE1), inorganic carbon uptake (CEMA_Q37050), light-independent chlorophyll biosynthesis (CHLI_Q94FT3), CCM proteins (CAH1_P20507 and CAH2_P24258), flagellar radial spoke protein or flagellar bending (RSP2_Q6UBQ3, RSP4_Q01656, and RSP5_Q27YU7), and PsbB mRNA maturation factor (MBB1_Q9FNS4_chloroplastic) are downregulated.

For the DEPs correlation network, every data point was reported as a node (each node represents a single protein), and each forms edges (neighboring interactions) with adjacent nodes (**Figures 10B, D, E**). Furthermore, red nodes that formed a dense network containing an average of ~15 interaction edges with neighboring nodes, which correspond to protein species related to PSII (PSBA_P07753 and PSBD_P06007), PSI (PSAG_P14224), chlorophyll-binding/light-harvesting (CB29_Q93WD2 and CB2_P14273), light-independent chlorophyll biosynthesis (CHLB_P37824), translational protein (RR2B_Q8HUH1 (putative 30S ribosomal S2-like protein), RR3_Q08365), and histone protein for DNA binding or nucleosome remodeling [H2B3_P54346 (Histone H2B.3) and H2B4_P54347 (Histone H2B.4)], are upregulated (**Figure 10D**). Similarly, in *pgr5*_500 vs. WT_50, 24 nodes formed a solid interaction network with 47 edges. To simplify the correlation matrix, each node was represented with a specific color (red—upregulated and green—downregulated) based on the degree of interaction. Furthermore, green nodes that formed a network containing an average of two interaction edges with neighboring nodes, which correspond to proteins related to PSII (PSBI_Q8DJZ6 and PSBO_P12853), PSI (PSAC_Q00914), heat shock proteins [HSP70_P25840 and H32_Q6LCW8 (Histone H3 type 2)], chlorophyll-binding/light-harvesting (CB29_Q93WD2), flagellar bending/calmodulin-binding [RSP2_Q6UBQ3 and RSP3_P12759 (Flagellar radial spoke protein 2 and 3)], and metabolic reaction (IPYR2_Q949J1 and S17P_P46284), are downregulated. Furthermore, red nodes that formed a weak network with neighboring nodes, which correspond to proteins related to PSII (PSBA_P07753 and PSBC_P10898), PSI (PSAB_P09144), ATP Synthesis (ATPD_Q42687), flagellar bending/calmodulin-binding RSP4_Q01656 (Flagellar radial spoke protein 4), RSP14_A8HNV0 (Radial spoke protein 14), heat shock protein [HS22C_P12811 (Heat shock 22 kDa protein_chloroplastic)], and Cyt *b_6_f* subunit for CET (PETD_P23230), are upregulated (**Figure 10F**).

## Discussion

Light stress has developed an accurate risk to *Chlamydomonas* growth and biomass development. *Chlamydomonas*’ regulating adaptation mechanisms have not been identified adequately under high light. Our recent report shows that cyclic electron transport is crucial in photoprotection, where we showed that *pgrl1* and *pgr5* play an essential role under high light stress (Yadav et al., 2020; Chouhan et al., 2023). In this study, we showed that NPQ is significantly increased in WT, whereas it is reduced in *pgrl1* and *pgr5*. However, the role of total proteins and the organization of thylakoid super-complexes are not yet understood under high light stress. This study comprehensively analyzed crucial PETC and metabolic proteins directly involved under high light. We have addressed the entire proteome profile and its relation to the cell’s function of photosynthesis efficiency under high light from WT, *pgrl1*, and *pgr5* mutants of *C. reinhardtii*.

### CET mutation reduced the capacity of oxygen uptake under high light stress

A high oxygen evolution has been observed for cells grown under high light in WT compared to growth light, indicating that the impact of high light was not drastic on PSII damage. The light-induced O_2_ uptake in *pgrl1* and *pgr5* was significantly reduced than in WT, demonstrating that the mutants’ lack of CEF affected acclimation to high light (**Figure 1A**; **Supplementary Figure 2B**). This result correlates with Chl fluorescence, which is similarly reduced in *pgrl1* and *pgr5* (**Supplementary Figure 2A**). Therefore, O_2_ evolved by chlororespiration, Mehler reaction, or the malate shunt, stimulating the recurrence of PSII photochemistry (Schreiber and Vidaver, 1974). These results suggest that cells adjust under high light stress by different adaptive mechanisms and show agreements with our biochemical data; i.e., oxygen-evolving complex subunit proteins PsbO and PsbP decreased in *pgrl1* and *pgr5* (**Figure 1C**; **Supplementary Figure 1D**). Moreover, proteome data showed that all the subunits of the oxygen-evolving complex are downregulated in *pgrl1* and *pgr5* than WT under high light (**Figure 7A**). These results indicated that WT acclimates better under high light, whereas *pgrl1* and *pgr5* mutants are more prone to photosystem damage.

### The pigment–protein composition and membrane stoichiometry were affected under high light in *pgrl1* and *pgr5*

In WT cells, the LHCSR3 and PSBS are defined under high light; therefore, the thylakoid membrane architecture and composition of PSII–LHCII super-complexes are not affected much (**Figure 3A**). The weaker *psi*-type CD signal in *pgrl1* and *pgr5* cells grown under high light indicates significant rearrangement in the macromolecular arrays of the PSII–LHCII complex (**Figures 2A, B**). The positive band at 506 nm in the Soret region originates from the β-carotene of the core complex (Tóth et al., 2016). It exhibits no or weak signal due to a decreased protein-containing core complex in cells subjected to high light stress (**Figures 2C, D**). The same result was observed from the composition of thylakoid membrane protein complexes in BN-PAGE analysis (**Figure 3**). In *C. reinhardtii*, PSII–LHCII comprises two reaction center cores and six LHCII trimers (Järvi et al., 2011). In *pgrl1* and *pgr5*, most of the LHCs of super-complex are associated with higher-order LHCs (LHC II trimers) or LHCII monomers under high light (**Figures 3A, B**). From BN-PAGE, owing to solubilization with β-DM, loosely bound trimers dissociated from PSII–LHCII super-complexes, leaving different core combinations with trimers and monomers of *pgrl1* and *pgr5* in high light (**Figure 3**). The abundance of super-complexes was not altered significantly in the *pgrl1*, but a dramatic change was observed in *pgr5* since it lacks LHCSR3 expression. The BN-PAGE result complements the CD results, where higher-order structures in the thylakoid membrane are affected under high light conditions. These observations conclude that the expression of LHCSR3 in high light could photo-protect the photosystems to hinder the over-excitation energy transfer from LHCII to RC.

### The protein profile analysis from immunoblotting provides insight into the *pgrl1* and *pgr5* under high light

The D1 and D2 proteins are the reaction center-binding proteins of PSII that carry out essential redox components for charge separation. The protein level changes in the cells grown in high light showed a marginal increase of D1 and D2 in WT (**Figures 4A, B**). In high light-grown cells, the D1 and D2 proteins accumulated more in *pgrl1* and *pgr5*; the continuous repair mechanisms change their abundance. PSII core protein synthesis and its photodamage repair depend on many factors that could affect the process of their acclimation resulting in minimal change in its content. The photodamage of PSII repair was inhibited by ROS production (Murata et al., 2007), and the translational initiation of PsbA transcripts was activated through a redox signal associated with electron transport in PSI and reduction of the plastoquinone pool (Trebitsh and Danon, 2001).

Our recent reports show that in *pgr5*, significant ROS production was observed under high light, which could be one of the reasons for the change in photosynthetic efficiency and proteins (Chouhan et al., 2022). Closely associated with these core proteins are core antennae chlorophyll-binding proteins such as CP43 and CP47. Its association with the PSII super-complex transfer excitation energy from light-harvesting complexes to the chlorophyll-binding protein CP43 is selectively inhibited compared to CP47, preventing excess excitation energy from the overloading reaction center to change the core antenna proteins’ content (Kim et al., 2017). The PsaA is significantly decreased in *pgr5* than in *pgrl1* and shows reduced protein content due to changes in chlorophyll and carotenoid molecules around the protein, which coordinates the cofactors to transfer electrons to ferredoxin (**Figures 4C, D**). The reduction of stromal subunits PsaD can distract the assembly of PSI. The associated stromal subunits PsaD and PsaE act as a ferredoxin binding site, leading to an efficient electron transport chain (Fischer et al., 1998).

The LHCII chlorophyll a/b-binding proteins were relatively consistent between WT and the *pgrl1* except for *pgr5* under high light. In the LHCII trimer, Lhcb1 and Lhcb3, peripherally arranged, depicted a drastic change in high light, while Lhcb2 near the PSII core showed no difference in protein content. This result indicates that high light mainly affects LHCs arranged peripherally in the super-complexes. The LHC monomeric protein, Lhcb5, reveals the decreased content, while there was no change in Lhcbm5, indicating that light stress mainly affects outermost arranged light-harvesting antennas. Light-harvesting complex proteins Lhcb3 and Lhcb5 were more tightly associated with the PSII core than Lhcb1 and Lhcb2 (Iwai et al., 2008). This might be the reason for the inhibition of outer antennae proteins.

An isoform of Lhcb4 connects trimeric LHCII with the PSII core; it stabilizes the PSII–LHCII dimer and binds to the monomeric core with the help of LHCII, suggesting its primary role in regulating the excitation energy flow and non-photochemical quenching process (Caffarri et al., 2009). Interestingly, the Lhcb4 has no significant change; thus, Lhcb4 might increase the rate of non-photochemical quenching, which is usually higher in high light conditions (**Figures 5A, C, D**). Furthermore, we examined the content of light-harvesting complex proteins of PSI that are arranged in two layers towards the stromal side of the PSI core. The outer half-ring of the super-complex includes Lhca4, Lhca5, and Lhca6, whereas the inner half-ring of the super-complex is composed of Lhca1, Lhca3, Lhca7, and Lhca8 with Lhca3 located near PsaK, as previously suggested (Drop et al., 2011; Yadavalli et al., 2011a; Ozawa et al., 2018). In assumption, eight LHCI subunits bind to PSAF in two layers from PsaG to the PsaK subunit. The innermost layer contains Lhca 1, 4, 6, and 5, and the outside layer comprises Lhca 3, 4, 6, and 7, which decreased severely in *pgr5* and might be the distracting assembly of PSI-LHCI under high light conditions.

Earlier, our group reported that under extreme high light conditions (1,000 µmol photons m^−2^ s^−1^), most of the inner LHCI proteins were affected by the ROS production in WT (Nama et al., 2019). Interestingly, under moderate or high light, we can say that the light-harvesting complexes of PSI are stable. However, their composition might be affected by a significant decrease in their binding site of PsaG and PsaH (**Figures 5B–D**). We recently reported that some of the core proteins were aggregated in moderately high light conditions, especially in *pgr5*, due to the induction of ROS (Chouhan et al., 2022). This could be the reason the efficiency of photosynthesis is reduced in *pgr5*. However, the stoichiometry of the PSI and PSII LHCs is maintained, indicating that *C. reinhardtii* acclimates rapidly by changing the antenna proteins under high light conditions.

### The proteome data supports the role of CET in maintenance of photosynthesis and photoprotection

Here, we have discussed the comparative analysis of Western blot and proteome data of the PETC protein. The phosphorylation and repair cycle of PSII prevents D1 degradation (Koivuniemi et al., 1995). Our data show significant downregulation of PSBA_P07753 (D1) and PSBD_P06007 (D2) in *pgrl1* and *pgr5* compared to WT high light, indicating that D1 incurs more damage under high light conditions, and it is in agreement with the Western blot data (**Figures 4A, B**). The primary antenna proteins PSBB_P37255 (CP43) and PSBC_P10898 (CP47) are downregulated, especially in *pgr5* than in *pgrl1*, as we can see in our Western blot data where the protein content was decreased in *pgr5* more than the *pgrl1* (**Figure 7B**). These outcomes designate that damaging the catalytic subunits D1 and D2 leads to the downregulation of the reaction centers associated with antenna protein PSBC_P10898 (CP43) and PSBB_P37255 (CP47) in the mutants under high light conditions (**Figure 7A**). This might be due to light stress, primarily phosphorylated D1, D2, and CP43 in threonine residues at N-terminal (Elich et al., 1992; Booij-James et al., 2002) by the serine and threonine kinase (Vainonen et al., 2005; Rochaix et al., 2012). 

The PSI reaction center proteins, PSAA_P12154, PSAB_P09144, PSAC_Q00914, PSAD_Q39615, PSAE_P12352, PSAF_P12356, PSAG_P14224, and PSAH_P13352, are downregulated in *pgrl1* and *pgr5* compared to WT, reflecting the importance of these proteins in the maintenance of the PSI assembly and CET under high light (**Figure 7A**). The LHCSR3 protein regulated NPQ (Peers et al., 2009) and showed a substantial change in *pgrl1* and *pgr5* compared to WT, signifying that CET and high light changed NPQ at the post-translational level. The NPQ is activated in the PSII antenna and disperses extra energy as heat (Ruban, 2016). Similarly, *Chlamydomonas* exhibited a substantial downregulation on LHCSR3.1_P0DO19 and LHCSR3.2_P0DO19 in *pgrl1* and *pgr5* due to the absence of CET under high light conditions. Another report showed that the LHCSR3 expression was supplementary for NPQ initiation in *Chlamydomonas* (Bonente et al., 2011). LHCSR1_P93664 was upregulated in *pgrl1* and *pgr5* and might be the zeaxanthin cycle compensated without CET under high light, which is also in line with our recent report (**Figure 7B**) (Chouhan et al., 2022). This finding aligns with NPQ data, showing that *pgr5* cannot induce NPQ, but *pgrl1* has a lower NPQ than WT under high light, to agree with our previous report (Yadav et al., 2020).

The PSBS1_A8HPM2 and PSBS2_A8HPM5 were upregulated, especially in *pgr5*; limited proton motive force (*pmf*) and LHCSR3 expression might increase the abundance of another photoprotective protein in *Chlamydomonas* under high light stress (**Figure 7B**). The PSBR_A0A2K3DMP5 is highly upregulated in *pgr5*, which offers that PSBR is essential for the steady binding of LHCSR3 to PSII–LHCII super-complexes and required for effective quenching under high light (Xue et al., 2015). The *pgr5* induced PSBS protein under high light stress (**Figure 7B**). The ATP synthase and Cyt *b_6_f* are two primary components of the PETC regulating the thylakoid lumen acidification or *pmf* under high light conditions. The ATP synthase subunits ATPA_P26526, ATPB_P06541, ATPE_P07891, and ATPG_P12113 are upregulated in WT than *pgrl1* and *pgr5* under high light. *pgr5* displays higher upregulation ATPD levels than WT, suggesting that most of the H^+^ is utilized of energy generation in the absence of CET in high light (**Supplementary Figure 4B**). The Cyt *b_6_f* complex highly regulates the thylakoid lumen acidification and CET pathway. The Cyt *b_6_f* complex subunit CYF_P23577, CYC6_P08197 (Cyt C6_chloroplastic), PETD_P23230, PETO_Q9LLC6, and PETM_Q42496 are upregulated in WT than *pgrl1* and *pgr5* under high light (**Figure 7A**). Another subunit of the Cyt *b_6_f* complex CYB6_Q00471 is extensively involved in the regulation of lumen acidification and is highly upregulated in *pgr5* mutant showing the importance of CET under high light stress.

### Whole-cell revealed significant changes in metabolomic protein under high light

It is known that photosynthesis is sensitive to high light. The change in photosynthesis would affect the metabolism of the cell. Also, the absence of the CET pathway alters the whole metabolic flux, leading to reroutes of the metabolic pathway and compositions under high light conditions to acclimate to the high light. Here, the Fructose bis-phosphate aldolase (ALFC_Q42690) was differentially expressed in WT, *pgrl1*, and *pgr5* in high light in gluconeogenesis and starch biosynthesis (**Figure 7C**). In addition, G3PA_P50362, engaged in glycerol synthesis, was the most upregulated in *pgr5* under high light. The G3PA_P50362 is also related to the conversion of DHAP to sn-glycerol-3-phosphate, and finally, glycerol kinase (GK) or G3P phosphatase (GPP) produces glycerol (Driever et al., 2017). The G3P is a predecessor for TAG biosynthesis and increases lipid synthesis under salinity stress (Herrera-Valencia et al., 2012). This could be the reason under high light, where a significant accumulation of lipids and TAG was observed in our recent report in order to photoprotect (Chouhan et al., 2022).

The Ferredoxin-NADP reductase (FENR_P53991), a chloroplastic protein involved in ATP and NADPH synthesis, was downregulated in *pgr5* under high light. These results suggested that, as in higher plants, reduction by the Fd-TRX system regulates G6PDH and PRK activity to optimize carbon reduction and oxidation in the chloroplast following the supply of electrons and response to the metabolic demands of ATP. The protein ENO_P31683 synthesized pyruvate from the D-glyceraldehyde 3-phosphate pathway for glycolysis, and carbohydrate degradation was differentially regulated in WT, *pgrl1*, and *pgr5* in high light. Thioredoxin M-type (TRXM_P23400, chloroplastic) are vital regulatory players receiving information about the redox state, and participating in reversible oxidation was highly upregulated in WT. The M type of TRX is known to activate NADP-malate dehydrogenase to hold the CET indirectly.

The TCA cycle protein malate dehydrogenase (MDHM_Q42686) was differentially regulated in WT, *pgrl1*, and *pgr5* under high light (**Figure 7C**). The MDHM converts oxaloacetate to malate, generating extra reducing power (Scheibe, 2004). Another mitochondrial enzyme, Glutathione peroxidase 1 (GPX1_P83564), was upregulated in WT and may constitute a glutathione peroxidase-like protective system against oxidative stresses synthesizing PUFA through arachidonic acid metabolic pathways and was differentially regulated in *pgrl1* and *pgr5* in high light. Phosphoenolpyruvate carboxylase 1 (Ppc1), the carboxylation of phosphoenolpyruvate (PEP) that forms oxaloacetate, was differentially regulated in WT, *pgrl1*, and *pgr5* in high light. It is activated by glutamine and dihydroxyacetone phosphate and inhibited by glutamate and malate. The enzymes of the CBB cycle are highly upregulated in WT, including the protein complexities involved in RuBisCO assembly (RUBA_Q42694 and RuBB_Q42693 proteins). The phosphoribokinase converts ribulose-5-phosphate to RuBP (ribulose-1,5-bisphosphate), the leading CO_2_-accepting enzyme differentially regulated in *pgrl1* and *pgr5* under high light. Rubisco uses RuBP as a substrate, creating two molecules of 3-phosphoglycerate. Increased expression of fructose bisphosphate enhances fixing CO_2_ fixation and RuBP renewal (Lefebvre et al., 2005) by refining photosynthesis under stress (Driever et al., 2017).

The reduced amount of P3GA_P50362 (Glyceraldehyde-3-phosphate dehydrogenase A_chloroplastic), synthesized by RuBisCO, activates high carbon sinks to fixed CO_2_ in high light-grown cultures. Lastly, the overexpression of these necessary CBB enzymes maintained strong protein translation abilities, i.e., 24 ribosomal proteins in which 17 were upregulated in WT and 7 were upregulated in *pgr5* under high light. The heatmap data show that 250 DEPs were divided into 49 Calvin cycle and metabolism protein sub-categories. The Carbonic anhydrase 1 (CAH1_P20507) and 2 (CAH2_P24258) proteins that play a crucial role in CCM responsible for the reversible carbonate dehydratase activity for the formation of CO_2_ were upregulated in WT, and remarkably downregulated in *pgrl1* and *pgr5* under high light stress (**Figure 7C**). The enhanced CBB protein in WT supports healthy photosynthetic growth. When photosynthesis is compromised, the starch converts into sugars and other metabolites to deliver carbon and energy under abiotic stress. Our recent laboratory reports show that *pgrl1* and *pgr5* accumulated significant lipids to acclimate to high light (Chouhan et al., 2022). The over-reduction of the PETC generates additional NADPH and NADH by mitochondria, which balances the equilibrium by cellular redox homeostasis under salt stress (Wingler, 2002).

### Heatmap, volcano plot, and Gene Ontology analysis revealed the CET-involved enrichment of photosynthesis proteins but significantly lacks a light-harvesting complex

The Gene Ontology analysis (**Figure 9**) and volcano plot data (**Figure 10**) revealed an unexpected enrichment of photosynthetic targets not described in previous studies in *Chlamydomonas* under high light. Similar to our heatmap data, GO proteins mainly engage in chlorophyll biosynthesis, photosynthetic electron transport chain, chlorophyll-binding, heat shock protein, light-harvesting, translational protein, and protein involved in interflagellar assembly (**Figure 9**). We have identified only two light-harvesting pigment chlorophyll a/b binding proteins CB29_Q93WD2 (Chlorophyll a-b binding protein CP29) and CB2_P14273 (Chlorophyll a-b binding protein of LHCII type I chloroplastic) upregulated in WT and *pgrl1* but downregulated in *pgr5* in high light that shows agreement with our protein data (**Figure 8C**). The remaining PSII–LHCII and PSI–LHCI proteins are not identified during the analysis and might be lost during protein extraction or are underneath the nLC/MS/MS detection limit.

## Conclusion

Targeted proteome analysis was conducted to compare the photosystem-related and central metabolic protein abundance in WT, *pgrl1*, and *pgr5*. The physiological and biochemical data show that the *pgr5* is prone to high light and observed compromised photosynthesis. Also, the thylakoid super-complexes and long-order array structure of LHCII were diminished in *pgr5* due to the induction of ROS. The proteomic data distinguish the possible molecular mechanism of CET on photosynthesis and metabolomic profile in response to high light. Our results specify a robust relationship between CET and Cyt *b_6_f* that impacts lumen acidification and NPQ. We have shown that mutants share mutual targets of CET that control photosynthetic machinery and compensation mechanisms, including regulation of *pmf* by Cyt *b_6_f* and ATP synthase activity that indirectly affect the photoprotective protein in high light. We have also identified that increased expression of PSBS protein in *pgr5* compared to WT acts independently, controlling NPQ in high light. In the GO and KEGG pathway studies, we recognized that high light affected photosynthesis, oxidative phosphorylation, and metabolic pathway proteins to prevent abolishing carbon assimilation; few of them play a significant role in photoprotection. The limitation of metabolite appears to be accessible via the TCA cycle affecting the restriction of CET. Generating additional NADPH by LET directly supplies the energy to carbon metabolism to eliminate ROS and neutralize the deleterious inhibitory effects. Our study reveals that CET is crucial in acclimation to the high light stress. Since the proteomic results have given a lot of clues, future efforts would address the role of these CET proteins *pgrl1* and *pgr5* in affecting the photosynthetic electron transport and inducing the downregulation of cellular metabolism in chloroplast independently or in coordination with mitochondria over different targets to maintain cellular homeostasis.

## Data availability statement

The datasets presented in this study can be found in online repositories. The names of the repository/repositories and accession number(s) can be found in the article/**Supplementary Material**.

## Author contributions

RY and RS planned the experiments and evaluated the results. RY, SM, MZ, and JP performed the experiments. RY and RS wrote the manuscript. All authors contributed to the article and approved the submitted version.
