# Supplementary material for: High light-induced changes in whole-cell proteomic profile and its correlation with the organization of thylakoid super-complex in cyclic electron transport mutants of Chlamydomonas reinhardtii
Source: Front Plant Sci. 2023 Jul 7;14:1198474. doi: 10.3389/fpls.2023.1198474 (PMC10374432; doi:10.3389/fpls.2023.1198474)
Supplement: Supplementary file 1 [file DataSheet_1.docx]

**Supplementary Information**

**High light-induced changes in whole-cell proteomic profile and its correlation with the organization of thylakoid supercomplex in cyclic electron transport mutants of *Chlamydomonas reinhardtii***

**Ranay Mohan Yadav^1^, Sureshbabu Marriboina^1^**^#^**, Yusaf Zamal Mohammad^1^, Jayendra Pandey^1^ and Rajagopal Subramanyam^1^***

Department of Plant Sciences, School of Life Sciences, University of Hyderabad, Gachibowli, Hyderabad- 500046, India

#Current Address: Ben-Gurion University of the Negev, Israel

***Corresponding author:**

Rajagopal Subramanyam ([srgsl@uohyd.ernet.in](mailto:srgsl@uohyd.ernet.in)) Phone: +91-40-23134572

**Supplemental Information**

**Figure** **S1**

**
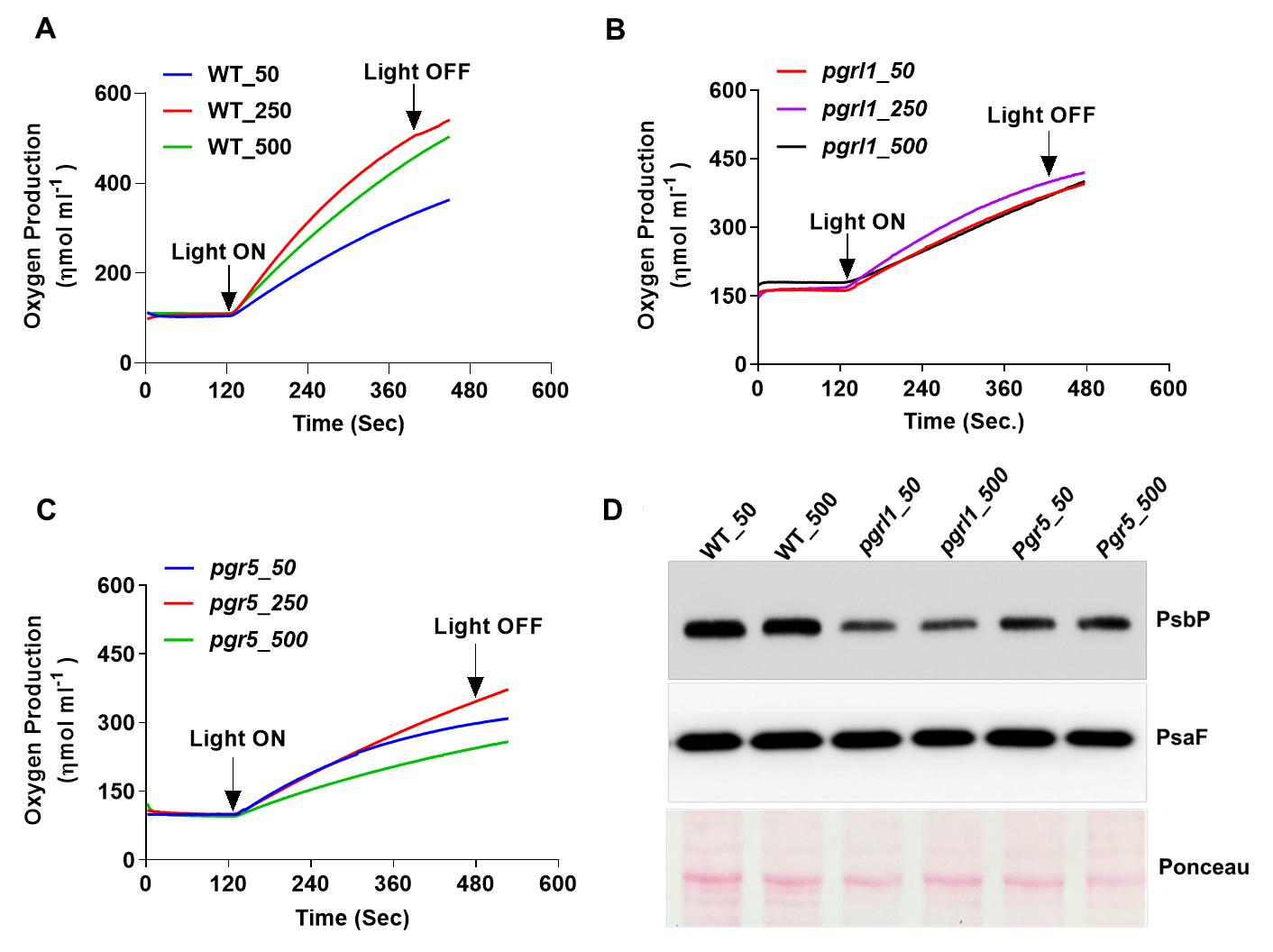
**

**Figure** **S1.** (A),(B) and (C) show that the net oxygen production (expressed as ƞmol ml^-1^) was measured at 25 ^0^C. Cells were collected at the mid-log growth phase and illuminated at 600 µmol m^-2^s^-1^ light intensity. The oxygen evolution was measured by selecting three average points after and before switching on and off the light during 8 min of exposure. In high light, the oxygen-evolving complex subunit of PsbP decreased in *pgrl1* and *pgr5* (D)*.* PsaF as a loading control and ponceau representative of equal loading of PsbP protein.

**Figure S2**

**
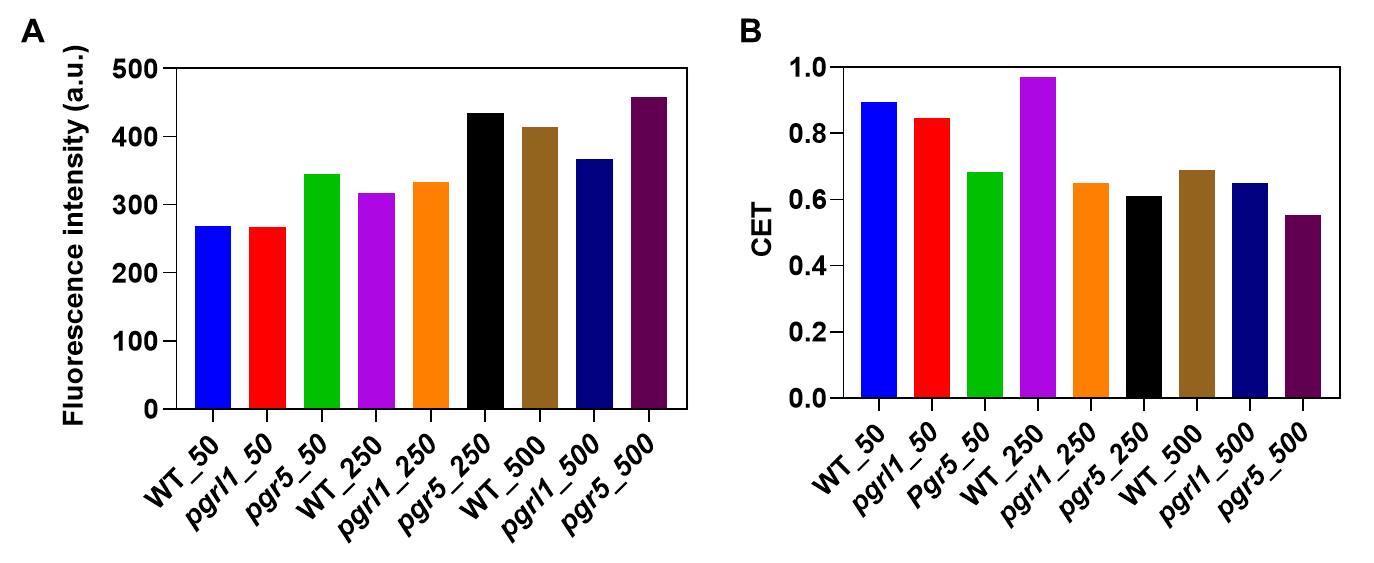
**

**Figure** **S2** Room temperature fluorescence (A) and CET rate (B) of WT, *pgrl1* and *pgr5* under growth (50 μmol photons m^-2^s^-1^) and moderate (250 μmol photons m^-2^s^-1^) and high light (500 μmol photons m^-2^s^-1^ ) conditions. The data shown here are representative of at least three biological replicates.

**Figure S3**

**
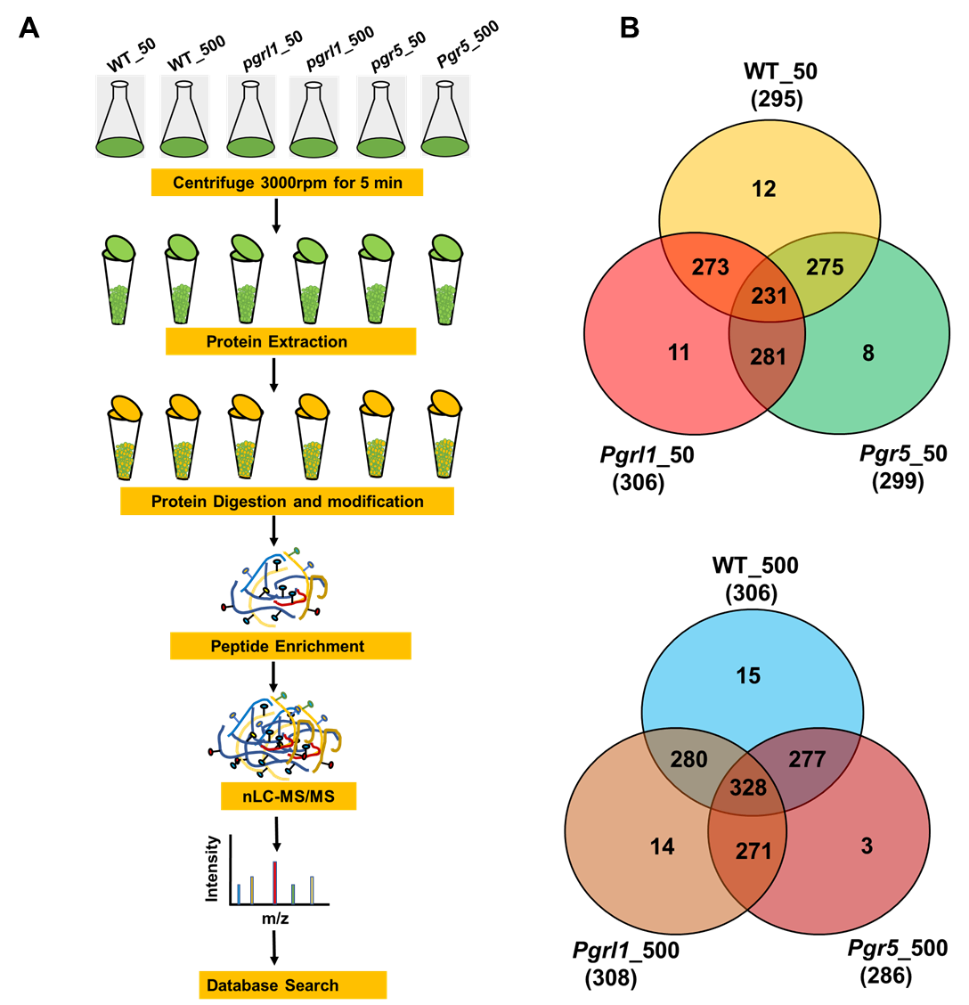
**

**Figure S3. Experimental setup for protein extraction, enrichment and database search**. Overview of the experimental design for analyzing the proteins in WT and mutants (*pgrl1*, *pgr5*) in growth and high light condition. The proteins were extracted with methanol: chloroform: water extraction solution, derivatized and measured with GC-MS. Proteins were isolated, digested, and desalted using a combined method. Subsequently, peptides were enriched and analysed by LC-MS/MS. (A) A Venn diagram of each protein was shown, which share common and exclusively expressed proteins WT, *pgrl1* and *pgr5* in growth and high light condition (B).

**Figure S4**

**
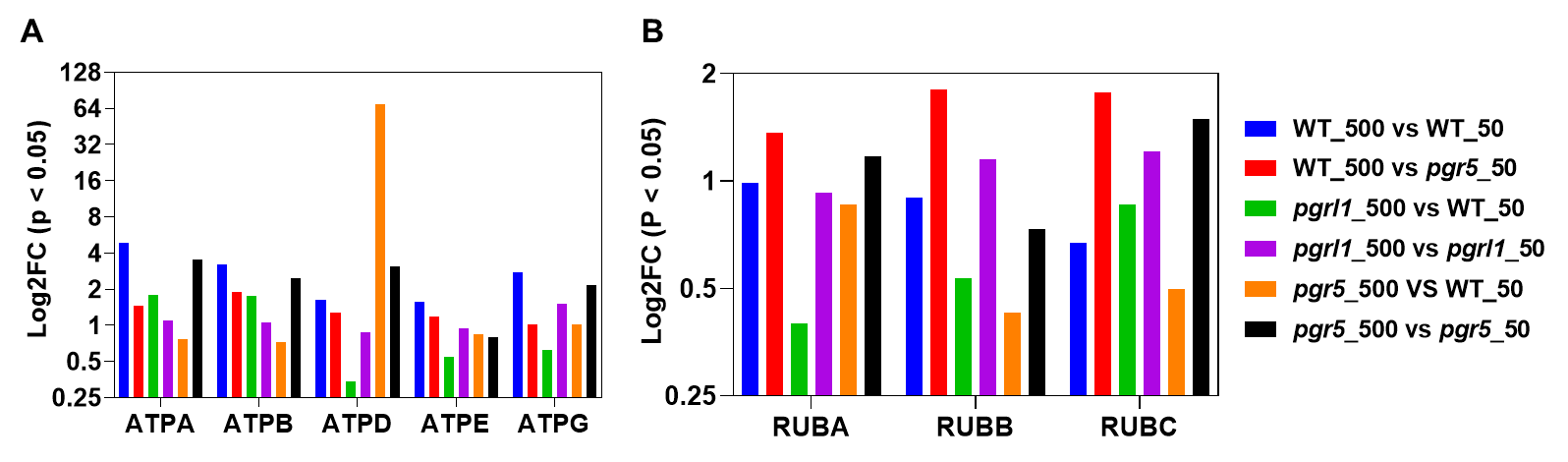
**

**Figure S4.** Proteins subunit participating in RubisCO assembly (A) and ATP synthesis (B) are involved in treated versus control, and p valve (p < 0.05) is shown (n = 3).

**Figure S5**

**
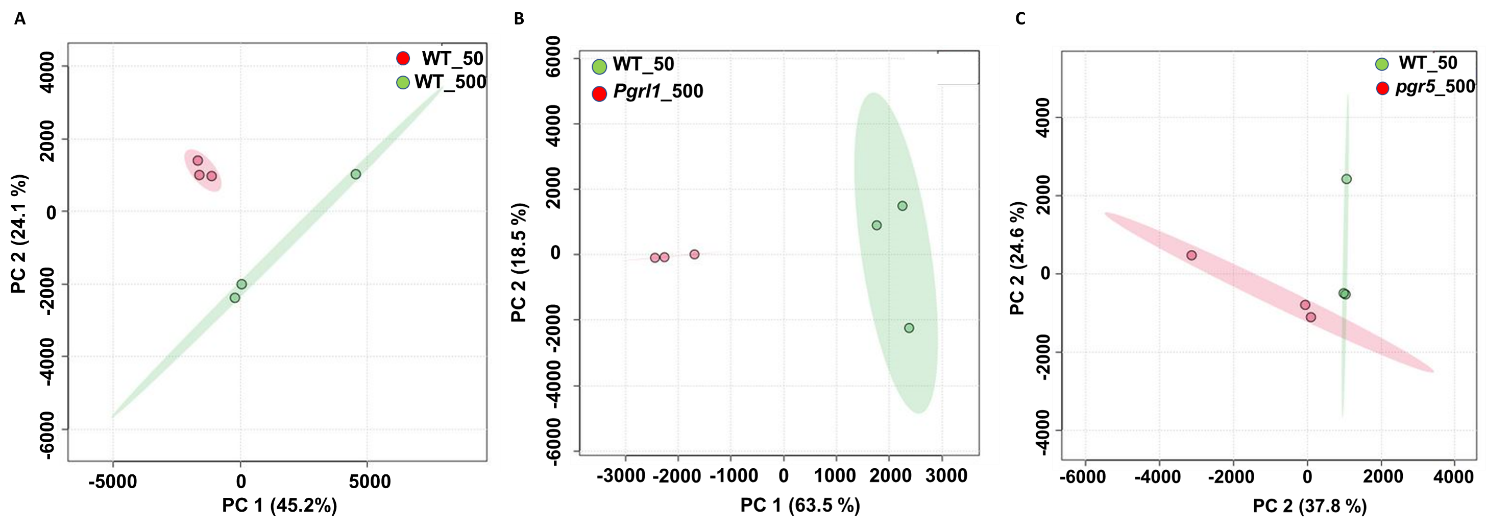
**

**Figure S6.** Principal component analysis (PCA) of three biological samples, i.e., WT and *pgrl1* and *pgr5* mutants grown at control 50 µmol photons m^-2^ s^-1^ and light treated with 500 µmol photons m^-2^ s^-1^. Principal component analysis (PCA) revealed variation in the proteome profile between the control and highlight treatments based on the first principal components (PC1) irrespective of 3 different samples (n=3 for each sample of control and lightly treated); they were separated based on the second principal components (PC2). High Light treatment and control samples are depicted in Red and green colours. The first principal component (PC1) explained 45.2% of the variation, with the 24.1% second PC2 in the WT sample being associated with the light treatments (A). The first principal component (PC1) explained that 63.5% of the variation in the data was associated with 18.5% second PC2 in the *pgrl1* sample being related to high light treatments (B). The third PC1 37.8% and the PC2 24.6% principal components separated the *pgr5* light-treated samples (C). Among these three samples (9 variants, each sample in triplicate) showed noteworthy changes between treatment and control (p < 0.05). Three different technical replicates were used for each biological sample.

**Figure S6**

**
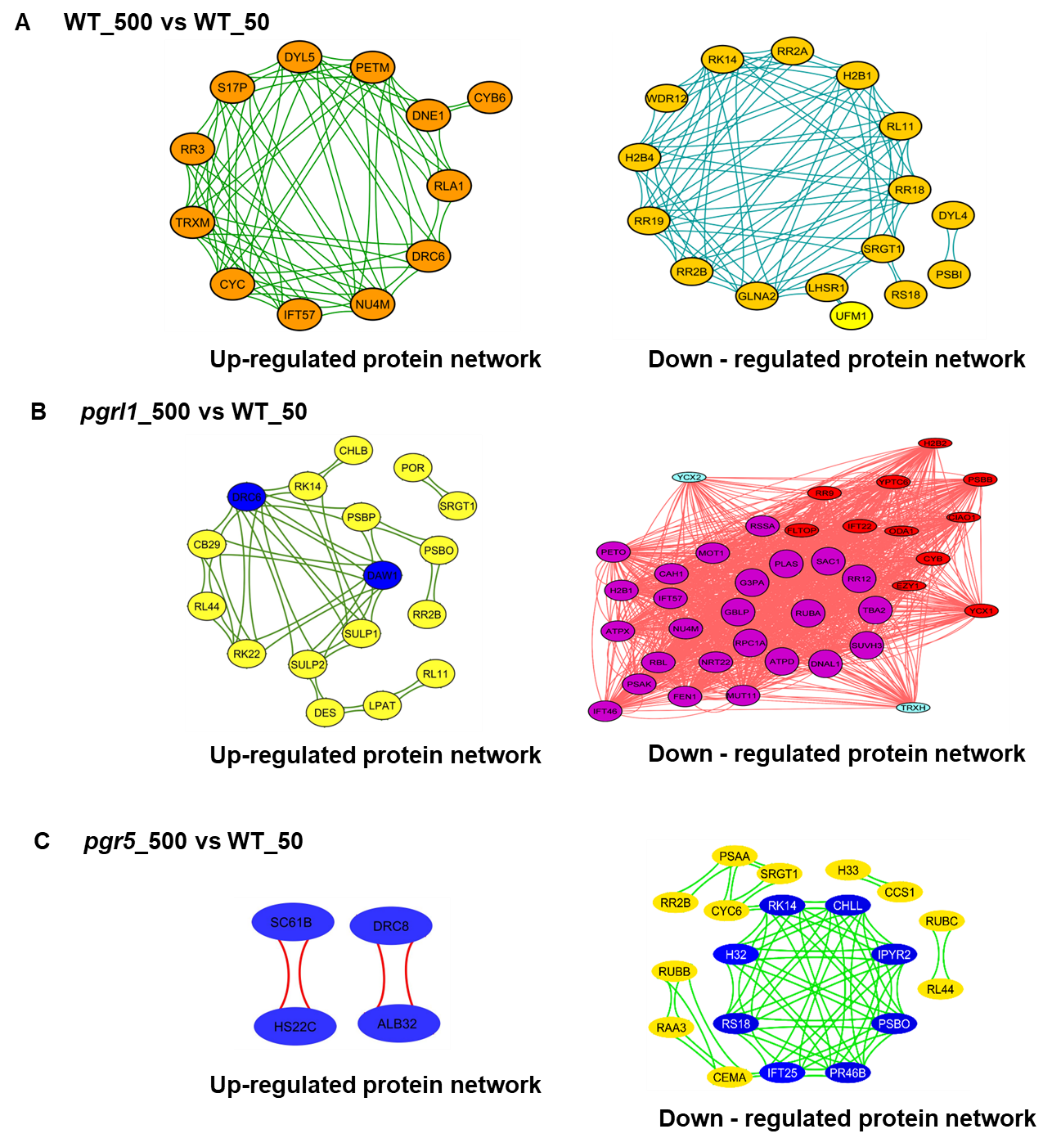
**

**Figure S6. Correlation network of upregulated and down-regulated proteins.** The Correlation network of upregulated (≥ 2.0 FC) and downregulated (≤ 0.5 FC) proteins is based on the Pearson correlation coefficient with probability threshold *P* < 0.05. Each node represented individual proteins with different colours based on the degree of interaction. The WT_50 vs WT_500 correlation network contains 12 upregulated proteins (maximum interaction 2-16) and 16 down-regulated proteins and most highly interconnected (maximum interaction 2-20) (A). A correlation network of *pgrl1*_500 vs WT_50 contains 17 up the protein in which blue colour showing maximunm interaction 13-14 with yellow colour proteins (total interaction 48) and 38 down-regulated protein and most of the highly interconnected (maximum interaction 78) and the interaction order was purple colour node (72-74 interaction) > Red colour node (60 - 68 interaction) > yellow colour node (42 - 48 interaction), respectively (B). A correlation network of *pgr5*_500 vs WT_50 contain 4 upregulated protein in which blue colour shows the minor interaction (total interaction 4) and 19 down-regulated protein, and the interaction order was blue colour node ( 10-15 interaction) > yellow colour node (2-10 interaction), respectively (C).

**Supplementary Table**

**Table S1** List of proteins expressed in WT_500 vs WT_50 (treated vs control) of *C. reinhardtii*

| **S.No.** | **Accession** | **Abbreviation** | **ProteinDescription** | **Fold change** | **p-value** | **GO Description and Pathway** |
| --- | --- | --- | --- | --- | --- | --- |
|  |  |  |  |  |  |  |
|  |  |  | **Down-regulated protein (FC ≤ 0.5)** |  |  |  |
|  |  |  |  |  |  |  |
| 1 | Q42689 | GLNA2 | Glutamine synthetase, chloroplastic | 0.04460096 | 0.800 | Glutamine biosynthetic process |
| 2 | P59776 | RR19 | 30S ribosomal protein S19, chloroplastic | 0.170332992 | 0.018 | Translation |
| 3 | O47027 | RR2A | 30S ribosomal protein S2, chloroplastic | 0.195929575 | 0.289 | Translation |
| 4 | H3JU05 | SRGT1 | Peptidyl serine alpha-galactosyltransferase | 0.278037308 | 0.435 | Transferring glycosyl groups |
| 5 | Q8HUH1 | RR2B | Putative 30S ribosomal S2-like protein | 0.292292572 | 0.040 | Translation |
| 6 | P46295 | RS14 | 40S ribosomal protein S14 | 0.336216482 | 0.521 | Translation |
| 7 | P54347 | H2B4 | Histone H2B.4 | 0.349937766 | 0.401 | Protein heterodimerization |
| 8 | P50565 | H2B1 | Histone H2B.1 | 0.367879441 | 0.081 | Protein heterodimerization |
| 9 | P49202 | RS18 | 40S ribosomal protein S18 | 0.371576687 | 0.101 | Translation |
| 10 | P11094 | RK14 | 50S ribosomal protein L14, chloroplastic | 0.398519034 | 0.127 | Translation |
| 11 | P50881 | RL11 | 60S ribosomal protein L11 | 0.410655759 | 0.612 | Translation |
| 12 | Q39591 | DYL4 | Dynein 14 kDa light chain | 0.410655759 | 0.241 | Outer dynein arm assembly |
| 13 | O20032 | RR18 | 30S ribosomal protein S18, chloroplastic | 0.414782914 | 0.313 | Translation |
| 14 | Q94EY2 | UFM1 | Ubiquitin-fold modifier 1 | 0.418951547 | 0.620 | Protein ufmylation |
| 15 | P12154 | PSAA | PSI P700 chlorophyll a apoprotein A1 | 0.440431658 | 0.386 | Photosynthesis |
| 16 | A8IR43 | WDR12 | Ribosome biogenesis protein | 0.467666431 | 0.244 | LSU-rRNA from tricistronic rRNA |
| 17 | P93664 | LHSR1 | LHC stress-related protein 1, chloroplastic | 0.477113911 | 0.989 | Non-photochemical quenching |
| 18 | P59763 | PSBI | Photosystem II reaction center protein I | 0.481908981 | 0.361 | Photosynthesis |
| 19 | Q8GTZ9 | CCS1 | Cytochrome c biogenesis, chloroplastic | 0.481908981 | 0.746 | Cytochrome complex assembly |
|  |  |  |  |  |  |  |
|  |  |  | **Differential regulated protein (≤ 2.0 FC ≥0.5)** |  |  |  |
|  |  |  |  |  |  |  |
| 20 | Q5DM57 | IF172 | Intraflagellar transport protein 172 | 0.506616989 | 0.505 | Multicellular organism development |
| 21 | P36437 | CHLB | Light-independent protochlorophyllide | 0.516851321 | 0.864 | Light-independent chl biosynthetic |
| 22 | A8JF71 | CTU1 | Cytoplasmic tRNA 2-thiolation protein 1 | 0.522045789 | 0.812 | Protein urmylation |
| 23 | Q37050 | CEMA | Chloroplast envelope membrane protein | 0.532591804 | 0.242 | Proton transmembrane transporter |
| 24 | A8J9T5 | THI4 | Thiamine thiazole synthase, chloroplastic | 0.537944435 | 0.183 | Thiamine biosynthetic process |
| 25 | P52908 | 1433 | 14-3-3-like protein | 0.537944435 | 0.030 | Flagellar arrangement and assembly |
| 26 | A8JA42 | IFT56 | Intraflagellar transport protein 56 | 0.543350861 | 0.121 | Intraciliary transport |
| 27 | Q00469 | CHLL | Light-independent protochlorophyllide | 0.543350861 | 0.244 | Photosynthesis, Dark reaction |
| 28 | A8JB22 | DRC2 | Dynein regulatory complex subunit 2 | 0.543350861 | 0.133 | Axonemal dynein complex assembly |
| 29 | A8IU92 | CFA20 | Cilia- and flagella-associated protein 20 | 0.559898376 | 0.042 | Axoneme, Cilium assembly |
| 30 | Q39617 | POR | Protochlorophyllide reductase, chloroplastic | 0.565525443 | 0.956 | Chlorophyll biosynthetic process |
| 31 | Q08355 | EZY2 | Ezy-1 protein | 0.571209062 | 0.915 | Uncharacterized protein |
| 32 | Q39604 | IDLC | 28 kDa inner dynein arm light chain | 0.571209062 | 0.655 | Cell motility |
| 33 | A8IHT2 | DRC11 | Dynein regulatory complex protein 11 | 0.571209062 | 0.833 | Ciliary/flagellar motility |
| 34 | P36495 | YCF78 | membrane protein ycf78 | 0.58274824 | 0.694 | Cell growth |
| 35 | Q9FEC4 | RAA3 | Trans-splicing factor Raa3, chloroplastic | 0.58274824 | 0.519 | mRNA processing, RNA splicing |
| 36 | A8J6J0 | SULT2 | Proton/sulfate cotransporter 2 | 0.588604987 | 0.271 | Sulfate transmembrane transporter |
| 37 | P14224 | PSAG | Photosystem I RC subunit V, chloroplastic | 0.594520559 | 0.081 | Photosynthesis |
| 38 | P48269 | CCSA | Cytochrome c biogenesis protein CcsA | 0.594520559 | 0.675 | cytochrome complex assembly |
| 39 | P37824 | CHLB | Light-independent protochlorophyllide | 0.600495585 | 0.417 | Light-independent chl biosynthetic |
| 40 | A8HYP5 | IFT43 | Intraflagellar transport protein 43 | 0.612626388 | 0.103 | Cell projection organization |
| 41 | P17746 | EFTU | Elongation factor Tu, chloroplastic | 0.625002269 | 0.388 | Translational elongation |
| 42 | Q39579 | DYL2 | Dynein 11 kDa light chain | 0.625002269 | 0.722 | Microtubule-based process |
| 43 | A8JC00 | RTCB | RNA-splicing ligase RtcB homolog | 0.625002269 | 0.179 | tRNA splicing |
| 44 | Q8HUH0 | RPC2B | DNA-directed RNA polymerase subunit beta | 0.63128364 | 0.882 | transcription, DNA-templated |
| 45 | Q27YU0 | RSP1 | Flagellar radial spoke protein 1 | 0.65050909 | 0.027 | Flagellar bending |
| 46 | A8I6P9 | SC61B | Protein transport protein Sec61 beta | 0.65050909 | 0.315 | Intracellular protein transport |
| 47 | Q39566 | GSA | Glutamate semialdehyde aminomutase | 0.657046828 | 0.419 | Chlorophyll biosynthetic process |
| 48 | P48270 | RR4 | 30S ribosomal protein S4, chloroplastic | 0.663650253 | 0.300 | Translation |
| 49 | Q39608 | NRT21 | Nitrate transporter 2.1 | 0.663650253 | 0.446 | Nitrate assimilation |
| 50 | A8JAN3 | POC16 | Centriole proteome protein 16 | 0.670320042 | 0.148 | Flagellum assembly |
| 51 | P05726 | RK16 | 50S ribosomal protein L16, chloroplastic | 0.670320042 | 0.007 | Mitochondrial translation |
| 52 | Q42695 | RUBC | RuBisCO large subunit- beta-2 | 0.670320042 | 0.103 | RuBisCO assembly |
| 53 | A8J6X7 | PCDP1 | Cilia- and flagella-associated protein 221 | 0.677056884 | 0.134 | Cilium assembly and movement |
| 54 | Q8RVC7 | SULP1 | Sulfate permease 1, chloroplastic | 0.677056884 | 0.654 | Uptake of sulfate |
| 55 | A8JAM0 | DRC7 | Dynein regulatory complex subunit 7 | 0.683861412 | 0.489 | Regulation of flagellar motility |
| 56 | A0A2K3DDJ2 | POB15 | Proteome of basal body protein 15 | 0.683861412 | 0.042 | Motility and sensing stimuli |
| 57 | P23489 | RCA | RuBP carboxylase/oxygenase activase, | 0.683861412 | 0.430 | Activation of RuBisCO |
| 58 | P14149 | RR12 | 30S ribosomal protein S12, chloroplastic | 0.683861412 | 0.007 | Translation |
| 59 | F5A894 | DAAF3 | Dynein assembly factor 3, axonema | 0.690734327 | 0.217 | Dynein complex assembly |
| 60 | Q39578 | DYI2 | Dynein, 78 kDa intermediate chain | 0.690734327 | 0.166 | Motor activity, Arm assembly |
| 61 | Q7XJ96 | DRC4 | Dynein regulatory complex subunit 4 | 0.690734327 | 0.039 | Dynein complex assembly |
| 62 | Q42690 | ALFC | Fructose-bisphosphate aldolase 1 | 0.704688094 | 0.262 | Glycolytic process |
| 63 | Q84V18 | STT7 | Serine/threonine-protein kinase | 0.71177032 | 0.023 | State transition |
| 64 | A8IRK7 | CF251 | Cilia- and flagella-associated protein 251 | 0.71177032 | 0.078 | Cilium movement |
| 65 | P41758 | PGKH | Phosphoglycerate kinase, chloroplastic | 0.718923724 | 0.946 | Glycolytic process |
| 66 | Q6QJE2 | SULP2 | Sulfate permease 2, chloroplastic | 0.718923724 | 0.289 | Sulfate transport |
| 67 | Q39615 | PSAD | PSI reaction center subunit II, chloroplastic | 0.718923724 | 0.612 | Ferredoxin-docking protein |
| 68 | P14273 | CB2 | Chl a-b binding protein LHCII type I | 0.718923724 | 0.720 | Light-harvesting as a light receptor |
| 69 | A8ITB0 | MOC1 | Holliday junction resolvase MO | 0.726149042 | 0.388 | DNA recombination |
| 70 | Q7YKX3 | RR11 | 30S ribosomal protein S11, chloroplastic | 0.726149042 | 0.111 | Translation |
| 71 | A8IB25 | RSSA | 40S ribosomal protein SA | 0.733446954 | 0.042 | Translation |
| 72 | Q2HWK7 | DES | Acyl-lipid omega-13 desaturase | 0.740818212 | 0.635 | Linoleic acid metabolic process |
| 73 | Q08354 | EZY1 | Ezy-1 protein | 0.748263574 | 0.857 | Uncharacterized protein |
| 74 | A8J2Z9 | FEN1 | Flap endonuclease 1 | 0.763379486 | 0.183 | Base-excision repair |
| 75 | A8HN58 | IFT27 | Intraflagellar transport protein 27 | 0.763379486 | 0.040 | GTPase activity |
| 76 | Q9XHH2 | DNAL1 | Dynein light chain 1, axonemal | 0.763379486 | 0.047 | Motor flagellar motility |
| 77 | A0A2K3D5Z7 | CMD1 | 5-methylcytosine-modifying enzyme 1 | 0.771051593 | 0.052 | 5-methylcytosine catabolic |
| 78 | P18068 | PLAS | Plastocyanin, chloroplastic | 0.786627865 | 0.014 | Electron transfer P700 and cyt b6f |
| 79 | F5A894 | PSAH | PSI reaction center subunit VI, chloroplastic | 0.786627865 | 0.033 | Docking of LHC I to PSI core |
| 80 | P81831 | CAPP1 | Phosphoenolpyruvate carboxylase 1 | 0.786627865 | 0.923 | TCA cycle |
| 81 | Q39584 | DYL3 | Dynein 18 kDa light chain | 0.786627865 | 0.017 | Motor activity |
| 82 | P93107 | PF20 | Flagellar WD repeat-containing protein Pf20 | 0.786627865 | 0.116 | Microtubule bridges in flagella |
| 83 | A8ID74 | BOP1 | Ribosome biogenesis protein BOP1 | 0.794533599 | 0.245 | Maturation of 5.8S rRNA |
| 84 | P12759 | RSP3 | Flagellar radial spoke protein 3 | 0.794533599 | 0.064 | Regulation of cilium-cell motility |
| 85 | Q8HTL6 | RPOB1 | DNA-directed RNA polymerase subunit beta | 0.794533599 | 0.110 | Transcription, DNA-templated |
| 86 | O20029 | RR9 | 30S ribosomal protein S9, chloroplastic | 0.802518799 | 0.419 | Translation |
| 87 | P46869 | FLA10 | Kinesin-like protein FLA10 | 0.802518799 | 0.019 | Intraciliary anterograde transport |
| 88 | P49213 | RL44 | 60S ribosomal protein L44 | 0.802518799 | 0.005 | Translation |
| 89 | A8HMZ4 | DRC5 | Dynein regulatory complex subunit 5 | 0.810584251 | 0.743 | Ciliary/flagellar motility |
| 90 | Q8W1K8 | MUT11 | Protein Mut11 | 0.810584251 | 0.093 | 'Lys-4' histone H3 methylation |
| 91 | Q8LPD9 | PHOT | Phototropin | 0.818730751 | 0.487 | Blue light signalling, NPQ induction |
| 92 | P27080 | ADT | ADP,ATP carrier protein | 0.826959136 | 0.039 | Catalyzes exchange of ADP and ATP |
| 93 | P54346 | H2B3 | Histone H2B.3 | 0.835270205 | 0.044 | DNA repair, Chromosomal stability |
| 94 | Q6R2V6 | CAPP2 | Phosphoenolpyruvate carboxylase 2 | 0.835270205 | 0.209 | Carbon fixation, TCA cycle |
| 95 | Q6UBQ3 | RSP2 | Flagellar radial spoke protein 2 | 0.843664815 | 0.064 | Flagellar bending |
| 96 | Q949J1 | IPYR2 | Soluble inorganic pyrophosphatase 2 | 0.852143792 | 0.680 | Phosphate metabolic process |
| 97 | P12356 | PSAF | PSI reaction center subunit III, chloroplastic | 0.860707971 | 0.131 | Electron transfer from PC to P700 |
| 98 | A8IUG5 | CFA99 | Cilia- and flagella-associated protein 99 | 0.869358235 | 0.038 | Ciliary/flagellar motility |
| 99 | A8IW99 | PLD6 | Mitochondrial cardiolipin hydrolase | 0.869358235 | 0.037 | Lipid catabolic process |
| 100 | B5BUZ8 | KTU | Protein kintoun | 0.869358235 | 0.010 | Dynein complex assembly |
| 101 | A8J0N6 | DRC10 | Dynein regulatory complex protein 10 | 0.869358235 | 0.087 | Maintains microtubule sliding |
| 102 | M1V4Y8 | CFA73 | Cilia- and flagella-associated protein 73 | 0.869358235 | 0.024 | Regulation of microtubule activity |
| 103 | A8JAF2 | CFA43_ | Cilia- and flagella-associated protein 43 | 0.878095435 | 0.005 | Flagellar organization and function |
| 104 | P54345 | H2B2 | Histone H2B.2 | 0.878095435 | 0.003 | Nucleosome remodelling |
| 105 | P46870 | KLP1 | Kinesin-like protein KLP1 | 0.878095435 | 0.095 | Microtubule-based movement |
| 106 | P25840 | HSP70 | Heat shock 70 kDa protein | 0.878095435 | 0.002 | Protein maturation, degradation |
| 107 | Q9SW75 | RL10A | 60S ribosomal protein L10a | 0.878095435 | 0.062 | Translation |
| 108 | A8JGF7 | LIAS | Lipoyl synthase, mitochondrial | 0.878095435 | 0.021 | Lipoylation peptidyl-N6-lipoyl-L-lysine. |
| 109 | O20031 | YCF3 | Photosystem I assembly protein Ycf3 | 0.878095435 | 0.119 | Chaperone- PSI subunits assembly |
| 110 | P53991 | FENR | Ferredoxin--NADP reductase, chloroplastic | 0.886920439 | 0.290 | Regulating the cyclic and non-CEF |
| 111 | Q00914 | PSAC | Photosystem I iron-sulfur center | 0.886920439 | 0.378 | Binding of PsaD and PsaE to PSI |
| 112 | P50362 | G3PA | G3P dehydrogenase A, chloroplastic | 0.886920439 | 0.042 | Glucose metabolic process |
| 113 | A0A2K3DZC4 | BSD2 | BUNDLE SHEATH DEFECTIVE 2, chloroplastic | 0.886920439 | 0.236 | Chaperone-mediated protein folding |
| 114 | A4GRC6 | HAP2 | Hapless 2 | 0.886920439 | 0.123 | Protein insertion into the membrane |
| 115 | Q42693 | RUBB | RuBisCO large subunit-binding beta-1 | 0.895834136 | 0.024 | Assembly of the enzyme oligomer |
| 116 | P0DL09 | DRC1 | Dynein regulatory complex protein 1 | 0.895834136 | 0.066 | Dynein complex assembly |
| 117 | A8I2V9 | LISC | Lipoyl synthase, chloroplastic | 0.895834136 | 0.169 | Protein lipoylation |
| 118 | P48267 | RR7 | 30S ribosomal protein S7, chloroplastic | 0.895834136 | 0.878 | Translation |
| 119 | O48513 | RL13 | 60S ribosomal protein L13 | 0.895834136 | 0.728 | Translation |
| 120 | A8JGB0 | ASNA1 | ATPase ARSA1 | 0.895834136 | 0.025 | ATPase activity |
| 121 | Q8S339 | ALB31 | ALBINO3-like protein 1, chloroplastic | 0.904837417 | 0.316 | Assembly and activity of LHC I and II |
| 122 | D4P3R7 | CFA74 | Cilia- and flagella-associated protein 74 | 0.904837417 | 0.028 | Cilium movement, Cell motility |
| 123 | P14217 | ARS | Arylsulfatase | 0.913931182 | 0.157 | Phenol metabolic process |
| 124 | A8IGK2 | EFTS | Elongation factor Ts, mitochondrial | 0.913931182 | 0.011 | Translation elongation factor activity |
| 125 | A8IRJ7 | CFA53 | Cilia- and flagella-associated protein 53 | 0.913931182 | 0.273 | Cilium assembly and movement |
| 126 | Q27YU7 | RSP5 | Flagellar radial spoke protein 5 | 0.923116348 | 0.021 | Regulation of flagellar bending |
| 127 | A8IH47 | CFA91 | Cilia- and flagella-associated protein 91 | 0.923116348 | 0.005 | Cilium movement |
| 128 | P00877 | RBL | RuBP carboxylase large chain | 0.923116348 | 0.121 | Photorespiration |
| 129 | P19824 | KPPR | Phosphoribulokinase, chloroplastic | 0.93239382 | 0.185 | Reductive pentose-phosphate cycle |
| 130 | Q39586 | HSTC | Homocysteine methyltransferase | 0.93239382 | 0.087 | Methionine biosynthetic process |
| 131 | A1JHN0 | HSTC | Homogentisate solanesyltransferase | 0.93239382 | 0.008 | Carotenoid and PQ biosynthetic |
| 132 | P09205 | TBA2 | Tubulin alpha-2 chain | 0.941764535 | 0.044 | Alters the microtubule cytoskeleton |
| 133 | Q42686 | MDHM | Malate dehydrogenase, mitochondrial | 0.941764535 | 0.070 | Carbohydrate metabolic process |
| 134 | A8IQE0 | CCD39 | Coiled-coil domain-containing protein 39 | 0.941764535 | 0.009 | Cilium movement |
| 135 | A8HYU5 | METK | S-adenosylmethionine synthase | 0.941764535 | 0.415 | S-adenosylmethionine biosynthetic |
| 136 | P29683 | CHLN | Light-independent protochlorophyllide | 0.941764535 | 0.059 | Light-independent chl biosynthetic |
| 137 | P09204 | TBA1 | Tubulin alpha-1 chain | 0.951229424 | 0.034 | Microtubule-based process |
| 138 | A8JID5 | CF157 | Cilia- and flagella-associated protein 157 | 0.951229424 | 0.050 | Cilium movement, Cell motility |
| 139 | P26565 | RK20 | 50S ribosomal protein L20, chloroplastic | 0.951229424 | 0.247 | Translation |
| 140 | Q39609 | NRT22 | Nitrate transporter 2.2 | 0.951229424 | 0.005 | Nitrate assimilation, Nitrate transport |
| 141 | Q5QD03 | SUVH3 | Histone-lysine N-methyltransferase | 0.951229424 | 0.015 | Methyltransferase repressed chromatin |
| 142 | P24258 | CAH2 | Carbonic anhydrase 2 | 0.951229424 | 0.136 | CCM, Carbonate dehydratase activity |
| 143 | P42380 | CLPP | ATP-dependent protease proteolytic | 0.96078944 | 0.575 | chymotrypsin-control protein misfolding |
| 144 | Q9GGE2 | RR14 | 30S ribosomal protein S14, chloroplastic | 0.970445534 | 0.102 | Translation |
| 145 | A2T2X4 | IFT46 | Intraflagellar transport protein 46 | 0.970445534 | 0.024 | Cilium-dependent cell motility |
| 146 | Q8HTL2 | RK2 | 50S ribosomal protein L2, chloroplastic | 0.970445534 | 0.093 | Mitochondrial translation |
| 147 | O63075 | ATPI | ATP synthase subunit a, chloroplastic | 0.980198674 | 0.986 | ATP synthesis coupled proton transport |
| 148 | Q8GSP8 | ZYS3 | Zygote-specific protein 3 | 0.980198674 | 0.508 | Cell development |
| 149 | Q09JZ4 | DAAF1 | Leucine-rich repeat-containing ODA7 | 0.990049834 | 0.394 | Cell motility and localization |
| 150 | Q42694 | RUBA | RuBisCO large subunit- alpha, chloroplastic | 0.990049834 | 0.165 | Assembly of the enzyme oligomer |
| 151 | Q9LEM8 | NAC2 | PsbD mRNA maturation factor, chloroplastic | 0.990049834 | 0.098 | mRNA processing- 5' UTR of psbD |
| 152 | Q9FNS4 | MBB1 | PsbB mRNA maturation factor, chloroplastic | 0.990049834 | 0.040 | mRNA processing via 5'-UTR of psbB |
| 153 | P53498 | ACT | Actin | 0.990049834 | 0.007 | Cytoplasmic streaming |
| 154 | Q8VXP3 | TBC2 | Tbc2 translation factor, chloroplastic | 1 | 0.126 | PsbC mRNA for translation initiation |
| 155 | P05724 | YCX3 | 14.4 kDa protein in 16S rRNA region | 1 | 0.149 | Determine phylogeny |
| 156 | Q9ZWM5 | CAO | Chlorophyllide a oxygenase, chloroplastic | 1 | 0.048 | Chlorophyll b biosynthetic process |
| 157 | A8HME3 | IFT22 | Intraflagellar transport protein 22 | 1.010050167 | 0.430 | Cellular availability of IFT particles |
| 158 | A8HNV0 | RSP14 | Radial spoke protein 14 | 1.010050167 | 0.092 | Cell motility, Endocytosis |
| 159 | P80028 | TRXH | Thioredoxin H-type | 1.010050167 | 0.056 | Cell redox homeostasis |
| 160 | Q9SMH4 | RAA2 | Trans-splicing factor Raa2, chloroplastic | 1.010050167 | 0.102 | RNA splicing, mRNA processing |
| 161 | P12113 | ATPG | ATP synthase gamma chain, chloroplastic | 1.02020134 | 0.008 | ATP synthesis coupled proton transport |
| 162 | A8HUA1 | CFA58 | Cilia- and flagella-associated protein 58 | 1.02020134 | 0.120 | Cilium-dependent cell motility |
| 163 | Q39573 | YPTC5 | GTP-binding protein YPTC5 | 1.02020134 | 0.121 | GTPase activity, protein transport |
| 164 | A8HPM5 | PSBS2 | Photosystem II protein PSBS2 | 1.030454533 | 0.225 | Nonphotochemical quenching |
| 165 | P06007 | PSBD | Photosystem II D2 protein | 1.040810773 | 0.013 | Photosynthetic Assembly of PSII |
| 166 | Q3Y8L7 | DAW1 | Dynein assembly WDR repeat domains 1 | 1.040810773 | 0.062 | Intraciliary transport and assembly |
| 167 | P12852 | PSBQ | Oxygen-evolving enhancer 3, chloroplastic | 1.040810773 | 0.051 | Photosynthesis |
| 168 | A8JFU2 | CFA65 | Cilia- and flagella-associated protein 65 | 1.040810773 | 0.002 | Cell projection organization |
| 169 | Q7XA07 | DC2L | Cytoplasmic dynein 2 intermediate chain 1 | 1.040810773 | 0.373 | Regulates flagellar dynein activity |
| 170 | P20507 | CAH1 | Carbonic anhydrase 1 | 1.040810773 | 0.111 | Carbonate dehydratase activity |
| 171 | P11471 | PSBP | Oxygen-evolving enhancer 2, chloroplastic | 1.040810773 | 0.161 | Photosynthesis oxygen evolution |
| 172 | D2K6F1 | SLT2 | Sodium/sulfate cotransporter 2 | 1.051271097 | 0.254 | Potassium ion transport |
| 173 | A8IW34 | PURA | Adenylosuccinate synthetase, chloroplastic | 1.051271097 | 0.323 | "de novo" AMP biosynthetic process |
| 174 | P0DO19 | LHR32 | LHC stress-related protein 3.1, chloroplastic | 1.051271097 | 0.012 | Nonphotochemical quenching |
| 175 | A8IQT2 | CCD40 | Coiled-coil domain-containing protein 40 | 1.051271097 | 0.541 | Assembly of the dynein complex |
| 176 | P31683 | ENO | Enolase | 1.061836545 | 0.040 | Glycolytic process |
| 177 | P0DO18 | LHR32 | LHC stress-related protein 3.2, chloroplastic | 1.061836545 | 0.025 | Nonphotochemical quenching |
| 178 | A8HQ54 | DRC9 | Dynein regulatory complex protein 9 | 1.061836545 | 0.122 | Regulator of ciliary/flagellar motility |
| 179 | Q9LLC6 | PETO | Cytochrome b6-f complex subuni | 1.061836545 | 0.080 | LET between PSII and I, CET around PSI |
| 180 | A8IVX2 | DRC3 | Dynein regulatory complex subunit 3 | 1.083287066 | 0.591 | Cilium-dependent cell motility |
| 181 | P37836 | PFL | Formate acetyltransferase | 1.083287066 | 0.056 | Convert pyruvate and coA |
| 182 | A8JBB2 | PESC | Pescadillo homolog | 1.094174288 | 0.399 | Maturation of 5.8S rRNA |
| 183 | A8IVJ1 | FLTOP | Protein Flattop homolog | 1.094174288 | 0.018 | Regulator-cilium basal body docking |
| 184 | P05722 | YCX2 | Uncharacterized protein in 16S rRNA region | 1.10517092 | 0.041 | Phylogenetic relationship |
| 185 | P83564 | GPX1 | Glutathione peroxidase 1, mitochondrial | 1.10517092 | 0.170 | Arachidonic acid metabolic process |
| 186 | P36443 | YCX6 | 12.3 kDa petA-petD intergenic region | 1.11627807 | 0.062 | Chloroplast based function |
| 187 | A8J1V4 | CFA44 | Cilia- and flagella-associated protein 44 | 1.11627807 | 0.058 | Cilium-dependent cell motility |
| 188 | Q6RCE1 | IFT74 | Intraflagellar transport protein 74 | 1.127496849 | 0.029 | Cilium assembly |
| 189 | Q39582 | TBG | Tubulin gamma chain | 1.127496849 | 0.066 | Cytoplasmic microtubule organization |
| 190 | A8HPM2 | PSBS1 | Photosystem II protein PSBS1 | 1.127496849 | 0.259 | Nonphotochemical quenching |
| 191 | Q8HTL7 | RPOB2 | DNA-directed RNA polymerase beta C | 1.127496849 | 0.069 | Transcription, DNA-templated |
| 192 | Q9LD46 | CRD1 | Mg-protoporphyrin IX monomethyl ester | 1.138828378 | 0.702 | Photosynthesis, Chl biosynthetic |
| 193 | A8IF44 | CFA61 | Cilia- and flagella-associated protein 61 | 1.138828378 | 0.012 | Cilium movement and organization |
| 194 | P23662 | CYB | Cytochrome b | 1.138828378 | 0.220 | Respiratory electron transport chain |
| 195 | P04352 | CALM | Calmodulin | 1.138828378 | 0.018 | Calcium-mediated signalling |
| 196 | Q9STD3 | CALR | Calreticulin | 1.138828378 | 0.331 | Protein folding |
| 197 | Q8HUG9 | RPC1A | DNA-directed RNA polymerase subunit beta | 1.1502738 | 0.069 | Transcription by RNA polymerase II |
| 198 | P45841 | RL31 | 60S ribosomal protein L31 | 1.16183425 | 0.057 | Translation |
| 199 | Q39570 | YPTC4 | GTP-binding protein YPTC4 | 1.16183425 | 0.048 | Protein transport and vesicular traffic |
| 200 | Q8HUH2 | RPOA | DNA-directed RNA polymerase alpha | 1.16183425 | 0.182 | Transcription, DNA-templated |
| 201 | P22675 | ARLY | Argininosuccinate lyase | 1.173510867 | 0.071 | Arginine biosynthetic via ornithine |
| 202 | Q42682 | HEM2 | Aminolevulinic acid dehydratase | 1.185304853 | 0.089 | Chlorophyll biosynthetic process |
| 203 | Q93Y52 | IPYR1 | Soluble inorganic pyrophosphatase 1 | 1.185304853 | 0.498 | Phosphate metabolic process |
| 204 | P07891 | ATPE | ATP synthase epsilon chain, chloroplastic | 1.185304853 | 0.367 | ATP synthesis coupled proton transport |
| 205 | A8HS48 | RS3A | 40S ribosomal protein S3a | 1.197217372 | 0.058 | Translation |
| 206 | A8INQ0 | ARL13 | ADP-ribosylation factor-like protein 13B | 1.197217372 | 0.021 | Control ciliary axoneme structure |
| 207 | P08739 | NU5M | NADH-ubiquinone oxidoreductase chain 5 | 1.197217372 | 0.002 | NADH dehydrogenase activity |
| 208 | A8J8F6 | TEKT | Tektin | 1.197217372 | 0.028 | Assembly of flagella and cilia |
| 209 | A8IHV3 | SLT3 | Probable sodium/sulfate cotransporter 3 | 1.197217372 | 0.175 | Potassium ion and Sulfate transport |
| 210 | P38482 | ATPBM | ATP synthase subunit beta, mitochondrial | 1.209249595 | 0.021 | ATP synthesis coupled proton transport |
| 211 | P12352 | PSAE | PSI reaction center subunit IV, chloroplastic | 1.221402762 | 0.679 | Photosynthesis |
| 212 | Q01656 | RSP4 | Flagellar radial spoke protein 4 | 1.221402762 | 0.040 | Cell motility, cilium assembly |
| 213 | A8ITV9 | CFA70 | Cilia- and flagella-associated protein 70 | 1.221402762 | 0.271 | Axoneme- regulates ciliary motility |
| 214 | Q39618 | SFAS | SF-assemblin | 1.233678052 | 0.079 | Striated microtubule-fibres assembly |
| 215 | A8HYJ1 | TOC34l | Translocase of chloroplast 34, chloroplastic | 1.233678052 | 0.030 | Transport cytoplasm to chloroplast |
| 216 | P50884 | RL12 | 60S ribosomal protein L12 | 1.246076729 | 0.026 | Translation |
| 217 | Q8HTL1 | RK5 | 50S ribosomal protein L5, chloroplastic | 1.246076729 | 0.106 | Translation |
| 218 | P50566 | H4 | Histone H4 | 1.258600015 | 0.147 | Nucleosome remodelling |
| 219 | A8IXB8 | ASNA2 | ATPase ARSA2 | 1.258600015 | 0.074 | Protein insertion into ER membrane |
| 220 | Q94EY1 | PR46B | Coiled-coil domain-containing protein 103 | 1.271249144 | 0.312 | Cilia motility |
| 221 | Q42688 | GLNA1 | Glutamine synthetase cytosolic isozyme | 1.271249144 | 0.035 | Glutamine biosynthetic process |
| 222 | A8IYS6 | CF300 | Cilia- and flagella-associated protein 300 | 1.271249144 | 0.205 | Structure organization and motility |
| 223 | A8IEF3 | ANM1 | Protein arginine N-methyltransferase 1 | 1.284025417 | 0.124 | Asymmetric dimethylation of flagellum |
| 224 | Q42687 | ATPD | ATP synthase delta chain, chloroplastic | 1.284025417 | 0.112 | ATP synthesis coupled proton transport |
| 225 | A8IB22 | CFA77 | Cilia- and flagella-associated protein | 1.284025417 | 0.039 | Cell motility |
| 226 | P23577 | CYF | Cytochrome f | 1.284025417 | 0.993 | CEF around PSI |
| 227 | A8J0J0 | LPAT | Acyl-sn-glycerol-phosphate acyltransferase | 1.323129814 | 0.973 | CDP-diacylglycerol biosynthetic process |
| 228 | P27766 | DYI3 | Dynein, 70 kDa intermediate chain | 1.336427477 | 0.035 | cell motility, dynein arm assembly |
| 229 | A6Q0K5 | CP12 | Calvin cycle protein CP12, chloroplastic | 1.349858824 | 0.012 | Assembly of complex of PRK/GAPDH |
| 230 | A8I9E8 | CFA45 | Cilia- and flagella-associated protein 45 | 1.363425117 | 0.210 | Cell motility |
| 231 | P12811 | HS22C | Heat shock 22 kDa protein, chloroplastic | 1.363425117 | 0.007 | Protein maturation and degradation |
| 232 | Q8HTL5 | ATPF | ATP synthase subunit b, chloroplastic | 1.363425117 | 0.022 | ATP synthesis coupled proton transport |
| 233 | P05723 | YCX1 | 12.2 kDa protein in 16S rRNA region | 1.377127754 | 0.034 | Conserved phylogeny |
| 234 | Q84U21 | RK22 | 50S ribosomal protein L22, chloroplastic | 1.377127754 | 0.066 | Translation |
| 235 | Q39593 | SAC1 | Putative sulfur deprivation regulator | 1.390968147 | 0.066 | Potassium ion transport |
| 236 | A8JF70 | ODA1 | Outer dynein arm protein 1 | 1.390968147 | 0.150 | Cell motility |
| 237 | I2CYZ4 | D4FAD | Acyl-lipid (7-3)-desaturase, chloroplastic | 1.404947596 | 0.357 | Fatty acid biosynthetic |
| 238 | P49728 | UCRIA | Cyt b6-f complex Fe-S subunit, chloroplastic | 1.41906754 | 0.024 | CET around PSI |
| 239 | P12853 | PSBO | Oxygen-evolving enhancer 1, chloroplastic | 1.433329435 | 0.003 | Primary site of water splitting |
| 240 | P31178 | GLE | Autolysin | 1.433329435 | 0.378 | Cell wall organization |
| 241 | P37255 | PSBB | Photosystem II CP47 RC protein | 1.447734622 | 0.039 | Photosynthesis, PET in PSII |
| 242 | P26526 | P26526 | ATP synthase subunit alpha, chloroplastic | 1.462284582 | 0.006 | ATP synthesis coupled proton transport |
| 243 | Q8LKI3 | ALB3.2 | ALBINO3-like protein 2, chloroplastic | 1.476980773 | 0.538 | Assembly of LHC I and II |
| 244 | Q6LCW8 | H32 | Histone H3 type 2 | 1.476980773 | 0.251 | Chromosomal stability. |
| 245 | P50564 | H32 | Histone H3 type 3 | 1.491824707 | 0.196 | Nucleosome remodelling |
| 246 | P07753 | PSBA | Photosystem II protein D1 | 1.521961536 | 0.030 | Photosynthesis, LET in PSII |
| 247 | A8I4E9 | CP100 | Cilia- and flagella-associated protein 100 | 1.521961536 | 0.162 | Cell motality, Cillium movement |
| 248 | Q42681 | H31 | Histone H3 type 1 | 1.537257535 | 0.088 | DNA binding, Chromosome stability |
| 249 | Q39572 | YPTC6 | Ras-related protein YPTC6 | 1.537257535 | 0.209 | GTPase activity |
| 250 | P05434 | CATR | Caltractin | 1.537257535 | 0.015 | Cell cycle, Cell division |
| 251 | A8IJF8 | SLT1 | Sodium/sulfate cotransporter 1 | 1.537257535 | 0.330 | Potassium ion transport |
| 252 | P0CH11 | RL401 | Ubiquitin-60S ribosomal protein L40 | 1.552707215 | 0.049 | Translation |
| 253 | A8IZG4 | CIAO1 | Cytosolic Fe-S protein assembly CIAO1 | 1.552707215 | 0.152 | Required iron-sulfur cluster assembly |
| 254 | P08475 | RBS2 | RuBisCO small chain 2, chloroplastic | 1.568312167 | 0.122 | Photosynthesis, Photorespiration |
| 255 | Q39571 | YPTC1 | GTP-binding protein YPTC1 | 1.568312167 | 0.118 | Protein transport, vesicular traffic |
| 256 | P51821 | ARF1 | ADP-ribosylation factor 1 | 1.568312167 | 0.133 | Vesicle-mediated transport |
| 257 | P37825 | YCX5 | Uncharacterized trnR-chlB intergenic region | 1.568312167 | 0.014 | Tag-protein stability |
| 258 | P09144 | PSAB | PSI P700 chlorophyll a apoprotein A2 | 1.599994191 | 0.031 | Primery electron donor of PSI |
| 259 | P49644 | G3PC | G3P dehydrogenase, cytosolic | 1.599994191 | 0.319 | Glycolysis, carbohydrate metabolism |
| 260 | A8ILK1 | CFA52 | Cilia- and flagella-associated protein 52 | 1.616074385 | 0.020 | Cell motility |
| 261 | A0A2K3DMP5 | PSBR | Photosystem II protein PSBR, chloroplastic | 1.632316236 | 0.039 | Energy-dependent quenching |
| 262 | Q68RJ5 | IFT81 | Intraflagellar transport protein 81 | 1.648721271 | 0.001 | Transport of tubulin within the cilium |
| 263 | P25387 | GBLP | Guanine nucleotide-binding protein- beta | 1.682027618 | 0.024 | Transcription initiation and termination |
| 264 | Q9AR22 | CTH1 | Mg-protoporphyrin monomethyl ester | 1.716006899 | 0.014 | Chlorophyll biosynthesis |
| 265 | P10898 | PSBC | PS II CP43 reaction center protein | 1.733253039 | 0.019 | PET in photosystem II |
| 266 | P00873 | RBS1 | RuBisCO small chain 1, chloroplastic | 1.750672504 | 0.091 | Photosynthesis, Photorespiration |
| 267 | P11660 | RTL | Reverse transcriptase-like protein | 1.786038401 | 0.816 | cDNA synthethesis |
| 268 | A6YCJ2 | MOT1 | Molybdate transporter 1 | 1.803988368 | 0.031 | Molybdate ion transporter activity |
| 269 | Q93WD2 | CB29 | Chlorophyll a-b binding protein CP29 | 1.840431425 | 0.042 | LHC Facilitates the State 1 to State 2 |
| 270 | A8J3A0 | DRC8 | Dynein regulatory complex protein 8 | 1.858928051 | 0.269 | Regulates microtubule sliding |
| 271 | Q9XF62 | DIP13 | 13 kDa deflagellation-inducible protein | 1.858928051 | 0.134 | Protein stability |
| 272 | P06541 | ATPB | ATP synthase subunit beta, chloroplastic | 1.87761057 | 0.066 | ATP synthesis coupled proton transport |
| 273 | Q8HTL3 | RK23 | 50S ribosomal protein L23, chloroplastic | 1.896480852 | 0.019 | Translation |
| 274 | Q42684 | SODM | Superoxide dismutase [Mn], mitochondrial | 1.993715528 | 0.440 | Destroys superoxide anion radicals |
|  |  |  |  |  |  |  |
|  |  |  | **Up-regulated proteins ( FC ≥ 2.0)** |  |  |  |
|  |  |  |  |  |  |  |
| 275 | P15451 | CYC | Cytochrome c | 2.013752683 | 0.002 | Carrier protein of mitochondrial ETC |
| 276 | P23400 | TRXM | Thioredoxin M-type, chloroplastic | 2.033991215 | 0.690 | Cell redox homeostasis |
| 277 | Q94FT3 | CHLI | Mg-chelatase subunit ChlI, chloroplastic | 2.075080647 | 0.015 | Insertion of Mg into protoporphyrin |
| 278 | P29763 | RLA1 | 60S acidic ribosomal protein P1 | 2.117000017 | 0.098 | Translational elongation |
| 279 | Q2XQY7 | IFT57 | Intraflagellar transport protein 57 | 2.1382762 | 0.030 | Maintenance and formation of cilia |
| 280 | Q39580 | DYL1 | Dynein 8 kDa light chain, flagellar outer arm | 2.1382762 | 0.012 | Microtubule-based process |
| 281 | P20113 | NU4M | NADH-ubiquinone oxidoreductase chain 4 | 2.247907992 | 0.001 | Electrons NADH to the respiratory chain |
| 282 | P05725 | DNE1 | DNA endonuclease I-CreI | 2.293318702 | 0.281 | Endonuclease activity, Intron homing |
| 283 | A8JHD7 | DRC6 | Dynein regulatory complex subunit 6 | 2.386910865 | 0.381 | Regulator of ciliary/flagellar motility |
| 284 | Q08365 | RR3 | 30S ribosomal protein S3, chloroplastic | 2.637944535 | 0.091 | Translational |
| 285 | P46284 | S17P | Sedoheptulose-bisphosphatase | 2.664456293 | 0.110 | Starch and Sucrose biosynthetic process |
| 286 | Q39592 | DYL5 | Dynein 16 kD light chain, flagellar outer arm | 3.12676832 | 0.122 | Cell redox homeostasis |
| 287 | Q42496 | PETM | Cyt b6f complex subunit 7, chloroplastic | 3.421229602 | 0.310 | CEF around PSI and state transitions. |
| 288 | Q00471 | CYB6 | Cytochrome b6 | 7.099327336 | 0.441 | CEF around PSI and state transitions. |
|  |  |  |  |  |  |  |
|  |  |  | **Control specific protein** |  |  |  |
|  |  |  |  |  |  |  |
| 289 | P07839 | FER | Ferredoxin, chloroplastic | Control_W50 |  | Electron transfer for metabolic reactions |
| 290 | P48268 | PSBE | Cytochrome b559 subunit alpha | Control_W50 |  | PETC, Charge separation |
| 291 | A8IC48 | URM1 | Ubiquitin-related modifier 1 homolog | Control_W50 |  | Endonuclease activity, Intron homing |
| 292 | B8LIX8 | IFT25 | Intraflagellar transport protein 25 | Control_W50 |  | Intraciliary transport |
| 293 | P08197 | CYC6 | Cytochrome c6, chloroplastic | Control_W50 |  | Electron carrier cyt b6f and PSI |
| 294 | P23230 | PETD | Cytochrome b6-f complex subunit 4 | Control_W50 |  | Transferring electrons within CET |
| 295 | Q5PU89 | UFM1 | Ubiquitin-fold modifier 1 | Treated_W50 |  | Protein ufmylation |
|  |  |  |  |  |  |  |
|  |  |  | **Treated specific protein** |  |  |  |
|  |  |  |  |  |  |  |
| 296 | P14225 | PSAK | PSI RC subunit psaK, chloroplastic | Treated_W500 |  | Photosynthesis |
| 297 | P59777 | PSAJ | PSI RC subunit IX | Treated_W500 |  | Organization of the PsaE and PsaF |
| 298 | P59775 | RR8 | 30S ribosomal protein S8, chloroplastic | Treated_W500 |  | Translation |
| 299 | P59774 | RK36 | 50S ribosomal protein L36, chloroplastic | Treated_W500 |  | Translation |
| 300 | A8JJB2 | MOC2A | Molybdopterin synthase sulfur ubunit | Treated_W500 |  | Mo-molybdopterin cofactor biosynthetic |
| 301 | O19930 | PSBJ | Photosystem II reaction center protein J | Treated_W500 |  | Photosynthesis, Charge separation |
| 302 | P50567 | H2A | Histone H2A | Treated_W500 |  | Chromosomal stability |
| 303 | A8ISN6 | ARL3 | ADP-ribosylation factor-like protein 3 | Treated_W500 |  | Cytokinesis and cilia signalling |
| 304 | P37256 | PSBT | Photosystem II reaction center protein T | Treated_W500 |  | Photosynthesis, PSII Dimerization |
| 305 | Q9FPQ6 | GP1 | Vegetative cell wall protein gp1 | Treated_W500 |  | Major component of outer cell wall |
| 306 | P08740 | NU2M | NADH-ubiquinone oxidoreductase chain 2 | Treated_W500 |  | NADH dehydrogenase activity |
| 307 | P11658 | NU1M | NADH-ubiquinone oxidoreductase chain 1 | Treated_W500 |  | NADH dehydrogenase activity |
| 308 | P32974 | PSBL | PSII reaction center protein L | Treated_W500 |  | PSII assembly and dimerization. |
|  | Q9SPI9 | PSBW | PSII RC W protein, chloroplastic | Treated_W500 |  | Stabilizes dimeric photosystem II |
| 309 | P04690 | TBB | Tubulin beta-1/beta-2 chain | Treated_W500 |  | Microtubule-based process |
| 310 | O20030 | YCF4 | Photosystem I assembly protein Ycf4 | Treated_W500 |  | Assembly of the PSI complex |

**Table S2** List of proteins expressed in *pgrl1*_500 vs WT_50 (treated vs control) of *C. reinhardtii*

| **S.No.** | **Accession** | **Abbreviation** | **Description** | **Fold change** | **P-value** | **GO description** |
| --- | --- | --- | --- | --- | --- | --- |
|  |  |  |  |  |  |  |
|  |  |  | **Down-regulated protein (FC ≤ 0.5)** |  |  |  |
|  |  |  |  |  |  |  |
| 1 | A8IVJ1 | FLTOP | Protein Flattop homolog | 0.101266466 | 0.042 | Regulator of cilium basal body docking |
| 2 | P14225 | PSAK | PSI RC subunit psaK, chloroplastic | 0.109700644 | 0.005 | Photosynthesis |
| 3 | P50565 | H2B1 | Histone H2B.1 | 0.205975089 | 0.003 | DNA binding |
| 4 | P14149 | RR12 | 30S ribosomal protein S12, chloroplastic | 0.218711891 | 0.021 | Translation |
| 5 | P50362 | G3PA | G3P-dehydrogenase A, chloroplastic | 0.225372653 | 0.012 | Glucose metabolic process |
| 6 | P80028 | TRXH | Thioredoxin H-type | 0.26447725 | 0.007 | Cell redox homeostasis |
| 7 | P20507 | CAH1 | Carbonic anhydrase 1 | 0.283654029 | 0.002 | Carbonate dehydratase activity |
| 8 | P20113 | NU4M | NADH-ubiquinone oxidoreductase chain 4 | 0.292292572 | 0.022 | ATP synthesis coupled transport |
| 9 | A8J785 | ATPX | ATP synthase subunit b', chloroplastic | 0.295230158 | 0.013 | ATP synthesis coupled proton transport |
| 10 | P25387 | GBLP | Guanine nucleotide-binding-subunit beta | 0.298197268 | 0.007 | Transcription initiation and termination |
| 11 | Q8W1K8 | MUT11 | Protein Mut11 | 0.304221247 | 0.013 | Involved 'Lys-4' histone H3 methylation |
| 12 | P49202 | IFT57 | 40S ribosomal protein S18 | 0.310366955 | 0.007 | Translation |
| 13 | Q2XQY7 | IFT57 | Intraflagellar transport protein 57 | 0.323033258 | 0.010 | Maintenance and formation of cilia |
| 14 | A2T2X4 | IFT46 | Intraflagellar transport protein 46 | 0.323033258 | 0.009 | Cilium-dependent cell motility |
| 15 | Q39593 | SAC1 | Sulfur deprivation response regulator | 0.326279793 | 0.054 | Potassium ion transport |
| 16 | Q42687 | ATPD | ATP synthase delta chain, chloroplastic | 0.339595511 | 0.001 | ATP synthesis coupled proton transport |
| 17 | O20029 | RR9 | 30S ribosomal protein S9, chloroplastic | 0.343008499 | 0.045 | Translation |
| 18 | P18068 | PLAS | Plastocyanin, chloroplastic | 0.349937766 | 0.032 | Electron transfer from P700 and cyt b_6_f |
| 19 | A6YCJ2 | MOT1 | Molybdate transporter 1 | 0.353454695 | 0.016 | Mo- transmembrane transporter activity |
| 20 | P05723 | YCX1 | 12.2 kDa protein in 16S rRNA region | 0.364218983 | 0.026 | Conserved phylogeny |
| 21 | Q9XHH2 | DNAL1 | Dynein light chain 1, axonemal | 0.364218983 | 0.005 | Motor activity for flagellar motility |
| 22 | P00877 | RBL | RuBP carboxylase large chain | 0.364218983 | 0.107 | Carboxylation of RuBP in PPP |
| 23 | A8HME3 | IFT22 | Intraflagellar transport protein 22 | 0.367879441 | 0.012 | Cellular availability of IFT particles |
| 24 | A8IZG4 | CIAO1 | Cytosolic Fe-S protein assembly protein | 0.367879441 | 0.001 | Required for Fe-S cluster assembly |
| 25 | P23662 | CYB | Cytochrome b | 0.379083027 | 0.025 | Respiratory electron transport chain |
| 26 | Q42694 | RUBA | RuBisCO large subunit-alpha, chloroplastic | 0.398519034 | 0.013 | Assembly of the RuBisCO enzyme |
| 27 | Q9LLC6 | PETO | Cyt b6f complex subunit petO, chloroplastic | 0.402524213 | 0.007 | LET between PSII and I, CETaround PSI |
| 28 | P54345 | H2B2 | Histone H2B.2 | 0.406569669 | 0.010 | Nucleosome remodelling |
| 29 | P08211 | RSSA | RuBP carboxylase large chain | 0.410655759 | 0.048 | Protein refolding |
| 30 | A8IB25 | RR4 | 40S ribosomal protein SA | 0.427414922 | 0.025 | Translation |
| 31 | P37255 | PSBB | Photosystem II CP47 RC protein | 0.431710535 | 0.004 | PET in photosystem II |
| 32 | A8J2Z9 | FEN1 | Flap endonuclease 1 | 0.444858065 | 0.388 | DNA replication, Base-excision repair |
| 33 | P09205 | TBA2 | Tubulin alpha-2 chain | 0.453844786 | 0.144 | Alters the microtubule cytoskeleton |
| 34 | Q39572 | YPTC6 | Ras-related protein YPTC6 | 0.453844786 | 0.726 | GTPase activity |
| 35 | Q39609 | NRT22 | Nitrate transporter 2.2 | 0.453844786 | 0.103 | nitrate assimilation, nitrate transport |
| 36 | A8JF70 | ODA1 | Outer dynein arm protein 1 | 0.453844786 | 0.005 | Cilium movement involved cell motility |
| 37 | P05725 | DNE1 | DNA endonuclease I-CreI | 0.472366553 | 0.008 | Endonuclease activity, Intron homing |
| 38 | P05722 | YCX2 | Uncharacterized 16S rRNA protein | 0.472366553 | 0.463 | Phylogenetic relationship |
| 39 | P48267 | RR7 | 30S ribosomal protein S7, chloroplastic | 0.472366553 | 0.015 | Translation |
| 40 | Q08354 | EZY1 | Ezy-1 protein | 0.477113911 | 0.035 | Uncharacterized protein |
| 41 | Q5QD03 | SUVH3 | Histone-lysine N-methyltransferase | 0.477113911 | 0.084 | Repressed chromatin function |
| 42 | Q8HUG9 | RPC1A | DNA-directed RNA polymerase subunit beta | 0.481908981 | 0.329 | Transcription by RNA polymerase II |
| 43 | P23489 | RCA | RuBisCO/oxygenase activase, chloroplastic | 0.50157607 | 0.008 | Activation of RuBisCO |
|  |  |  |  |  |  |  |
|  |  |  | **Differential regulated protein (≤ 2.0 FC ≥0.5)** |  |  |  |
|  |  |  |  |  |  |  |
| 44 | P49644 | G3PC | G3P-dehydrogenase, cytosolic | 0.506616989 | 0.013 | Carbohydrate metabolism |
| 45 | P04352 | CALM | Calmodulin | 0.506616989 | 0.005 | Calcium-mediated signalling |
| 46 | P0CH10 | RL403 | Ubiquitin-60S ribosomal protein L40 | 0.516851321 | 0.032 | Translation |
| 47 | P23400 | TRXM | Thioredoxin M-type, chloroplastic | 0.516851321 | 0.217 | Cell redox homeostasis |
| 48 | A8J9T5 | THI4 | Thiamine thiazole synthase, chloroplastic | 0.516851321 | 0.014 | Thiamine biosynthetic process |
| 49 | A8IB22 | CFA77 | Cilia- and flagella-associated protein | 0.522045789 | 0.002 | Cell motility |
| 50 | P04690 | TBB | Tubulin beta-1/beta-2 chain | 0.522045789 | 0.018 | Microtubule-based process |
| 51 | A8IEF3 | ANM1 | Protein arginine N-methyltransferase 1 | 0.527292432 | 0.839 | Asymmetric dimethylation of a flagellum |
| 52 | Q42693 | RUBB | RuBisCO large protein subunit beta-1 | 0.532591804 | 0.022 | Assembly of the enzyme oligomer |
| 53 | A8IQE0 | CCD39 | Coiled-coil domain-containing protein 39 | 0.532591804 | 0.053 | Inner dynein arm assembly |
| 54 | Q08355 | EZY2 | Ezy-1 protein | 0.532591804 | 0.009 | Uncharacterized protein |
| 55 | Q9LEM8 | NAC2 | PsbD mRNA maturation factor, chloroplastic | 0.532591804 | 0.021 | mRNA processing- 5' UTR of psbD mRNA |
| 56 | A8JBB2 | PESC | Pescadillo homolog | 0.537944435 | 0.080 | Maturation of 5.8S rRNA |
| 57 | A8IR43 | WDR12 | Ribosome biogenesis protein | 0.537944435 | 0.003 | LSU-rRNA from tricistronic rRNA |
| 58 | Q8GSP8 | ZYS3 | Zygote-specific protein 3 | 0.537944435 | 0.005 | Cell development |
| 59 | Q94FT3 | CHLI | Mg-chelatase subunit ChlI, chloroplastic | 0.537944435 | 0.002 | Insertion of Mg into protoporphyrin ring |
| 60 | P27766 | DYI3 | Dynein, 70 kDa intermediate chain | 0.543350861 | 0.073 | Cell motility, Outer dynein arm assembly |
| 61 | P49728 | UCRIA | Cyt b6-f complex Fe-S subunit, chloroplastic | 0.543350861 | 0.052 | Mediates LET and CET around PSI |
| 62 | P09204 | TBA1 | Tubulin alpha-1 chain | 0.548811623 | 0.108 | Microtubule-based process |
| 63 | A8HUA1 | CFA58 | Cilia- and flagella-associated protein 58 | 0.548811623 | 0.087 | Cilium-dependent cell motility |
| 64 | Q01656 | RSP4 | Flagellar radial spoke protein 4 | 0.548811623 | 0.025 | Cell motility, cilium assembly |
| 65 | A8I6P9 | SC61B | Protein transport protein Sec61 subunit beta | 0.548811623 | 0.041 | Intracellular protein transport |
| 66 | Q42496 | PETM | Cyt b6f complex subunit 7, chloroplastic | 0.548811623 | 0.015 | CEF around PSI and state transitions. |
| 67 | A8ITV9 | CFA70 | Cilia- and flagella-associated protein 70 | 0.548811623 | 0.019 | Axoneme- regulates ciliary motility |
| 68 | P07891 | ATPE | ATP synthase epsilon chain, chloroplastic | 0.548811623 | 0.099 | ATP synthesis coupled proton transport |
| 69 | A8ID74 | BOP1 | Ribosome biogenesis protein BOP1 homolog | 0.554327299 | 0.014 | Maturation of 5.8S rRNA |
| 70 | P93664 | LHSR1 | LHC stress-related protein 1, chloroplastic | 0.554327299 | 0.056 | Non-photochemical quenching |
| 71 | A8INQ0 | ARL13 | ADP-ribosylation factor-like protein 13B | 0.554327299 | 0.038 | Control ciliary axoneme structure |
| 72 | P53991 | FENR | Ferredoxin--NADP reductase, chloroplastic | 0.554327299 | 0.012 | Regulating the cyclic and non-CEF |
| 73 | Q09JZ4 | DAAF1 | Leucine-rich containing protein ODA7 | 0.565525443 | 0.062 | Cell motility and localization to organelle |
| 74 | P53498 | ACT | Actin | 0.565525443 | 0.051 | Cytoplasmic streaming |
| 75 | P24258 | CAH2 | Carbonic anhydrase 2 | 0.565525443 | 0.004 | CCM, Carbonate dehydratase activity |
| 76 | A8JGB0 | ASNA1 | ATPase ARSA1 | 0.565525443 | 0.009 | ATPase activity |
| 77 | P0CH11 | RL401 | Ubiquitin-60S ribosomal protein L40 | 0.571209062 | 0.005 | Translation |
| 78 | A8IUG5 | CFA99 | Cilia- and flagella-associated protein 99 | 0.571209062 | 0.005 | Ciliary/flagellar motility |
| 79 | A8J3A0 | DRC8 | Dynein regulatory complex protein 8 | 0.571209062 | 0.266 | Regulates microtubule sliding |
| 80 | Q42686 | MDHM | Malate dehydrogenase, mitochondrial | 0.571209062 | 0.047 | Carbohydrate metabolic process |
| 81 | B5BUZ8 | KTU | Protein kintoun | 0.571209062 | 0.017 | Axonemal dynein complex assembly |
| 82 | P12811 | HS22C | Heat shock 22 kDa protein, chloroplastic | 0.571209062 | 0.016 | Protein maturation and degradation |
| 83 | P93107 | PF20 | Flagellar WD repeat-containing protein Pf20 | 0.571209062 | 0.028 | Inter-microtubule bridges in flagella |
| 84 | A8HYJ1 | TOC34l | Translocase of chloroplast 34, chloroplastic | 0.571209062 | 0.373 | Transport- Cytoplasm to Chloroplast |
| 85 | P15451 | CYC | Cytochrome c | 0.576949804 | 0.004 | Final mitochondrial ETC protein carrier |
| 86 | Q39570 | YPTC4 | GTP-binding protein YPTC4 | 0.576949804 | 0.038 | Protein transport and vesicular traffic |
| 87 | A8IRK7 | CF251 | Cilia- and flagella-associated protein 251 | 0.576949804 | 0.108 | Cilium movement |
| 88 | Q39608 | NRT21 | Nitrate transporter 2.1 | 0.576949804 | 0.000 | Nitrate assimilation |
| 89 | P41758 | PGKH | Phosphoglycerate kinase, chloroplastic | 0.58274824 | 0.014 | Glycolytic process, Reductive PPP |
| 90 | P13352 | PSAH | Photosystem I RC subunit VI, chloroplastic | 0.588604987 | 0.063 | Docking for LHC I antenna |
| 91 | P12356 | PSAF | PSI RC subunit III, chloroplastic | 0.594520559 | 0.007 | Electron transfer from PC to P700 |
| 92 | A8JC00 | RTCB | RNA-splicing ligase RtcB homolog | 0.594520559 | 0.400 | tRNA splicing |
| 93 | P05434 | CATR | Caltractin | 0.594520559 | 0.026 | Cell cycle, Cell division |
| 94 | P48270 | RR4 | 30S ribosomal protein S4, chloroplastic | 0.600495585 | 0.011 | Translation |
| 95 | A6Q0K5 | CP12 | Calvin cycle protein CP12, chloroplastic | 0.600495585 | 0.000 | Assembly of complex of PRK/GAPDH. |
| 96 | Q9SW75 | RL10A | 60S ribosomal protein L10a | 0.600495585 | 0.006 | Translation |
| 97 | Q8HUH2 | RPOA | DNA-directed RNA polymerase subunit alpha | 0.600495585 | 0.001 | Transcription, DNA-templated |
| 98 | A4GRC6 | HAP2 | Hapless 2 | 0.600495585 | 0.001 | Protein insertion into the membrane |
| 99 | Q9AR22 | CTH1 | MG-protoporphyrin IX monomethyl ester | 0.60653066 | 0.009 | Photosynthesis, chlorophyll biosynthesis |
| 100 | Q39579 | DYL2 | Dynein 11 kDa light chain | 0.612626388 | 0.037 | Microtubule-based process |
| 101 | P12113 | ATPG | ATP synthase gamma chain, chloroplastic | 0.618783398 | 0.060 | ATP synthesis coupled proton transport |
| 102 | Q39618 | SFAS | SF-assemblin | 0.618783398 | 0.004 | Striated microtubule-fibres assembly |
| 103 | A8IF44 | CFA61 | Cilia- and flagella-associated protein 61 | 0.618783398 | 0.029 | Cilium movement, Cilium organization |
| 104 | Q37050 | CEMA | Chloroplast envelope membrane protein | 0.618783398 | 0.029 | Transmembrane transporter activity |
| 105 | F5A894 | DAAF3 | Dynein assembly factor 3, axonema | 0.625002269 | 0.023 | Axonemal dynein complex assembly |
| 106 | P0DL09 | DRC1 | Dynein regulatory complex protein 1 | 0.625002269 | 0.029 | Dynein complex assembly |
| 107 | A8ISN6 | ARL3 | ADP-ribosylation factor-like protein 3 | 0.625002269 | 0.002 | Cytokinesis and cilia signalling |
| 108 | A8J6J0 | SULT2 | Proton/sulfate cotransporter 2 | 0.625002269 | 0.001 | Sulfate transmembrane transporter |
| 109 | A8IW99 | PLD6 | Mitochondrial cardiolipin hydrolase | 0.637628159 | 0.020 | Lipid catabolic process |
| 110 | P29763 | RLA1 | 60S acidic ribosomal protein P1 | 0.637628159 | 0.005 | Translational elongation |
| 111 | Q8HTL5 | ATPF | ATP synthase subunit b, chloroplastic | 0.637628159 | 0.031 | ATP synthesis coupled proton transport |
| 112 | Q42690 | ALFC | Fructose-bisphosphate aldolase 1 | 0.644036423 | 0.022 | Glycolytic process |
| 113 | Q39586 | HSTC | Homocysteine methyltransferase | 0.644036423 | 0.011 | Methionine biosynthetic process |
| 114 | Q00469 | CHLL | Light-independent protochlorophyllide | 0.644036423 | 0.000 | Photosynthesis, Dark reaction |
| 115 | M1V4Y8 | CFA73 | Cilia- and flagella-associated protein 73 | 0.65050909 | 0.031 | Regulation of microtubule motor activity |
| 116 | Q8HTL6 | RPOB1 | DNA-directed RNA polymerase beta N | 0.65050909 | 0.014 | Transcription, DNA-templated |
| 117 | P31683 | ENO | Enolase | 0.663650253 | 0.002 | Glycolytic process |
| 118 | A8IU92 | CFA20 | Cilia- and flagella-associated protein 20 | 0.663650253 | 0.132 | Axoneme, Cilium assembly |
| 119 | A8J1V4 | CFA44 | Cilia- and flagella-associated protein 44 | 0.670320042 | 0.051 | Cilium-dependent cell motility |
| 120 | P38482 | ATPBM | ATP synthase subunit beta, mitochondrial | 0.670320042 | 0.001 | ATP synthesis coupled proton transport |
| 121 | P0DO19 | LHR32 | LHC stress-related protein 3.1, chloroplastic | 0.677056884 | 0.015 | Nonphotochemical quenching |
| 122 | Q6UBQ3 | RSP2 | Flagellar radial spoke protein 2 | 0.677056884 | 0.048 | Flagellar bending |
| 123 | P08475 | RBS2 | RuBisCO small chain 2, chloroplastic | 0.683861412 | 0.003 | Photorespiration, Carbon fixation |
| 124 | P0DO18 | LHR32 | LHC stress-related protein 3.2, chloroplastic | 0.683861412 | 0.016 | Nonphotochemical quenching |
| 125 | Q8HTL7 | RPOB2 | DNA-directed RNA polymerase beta C | 0.683861412 | 0.033 | Transcription, DNA-templated |
| 126 | Q9STD3 | CALR | Calreticulin | 0.683861412 | 0.860 | Protein folding |
| 127 | P00873 | RBS1 | RuBisCO small chain 1, chloroplastic | 0.690734327 | 0.004 | Photosynthesis, Photorespiration |
| 128 | Q84V18 | ALFC | Serine/threonine-kinase, chloroplastic | 0.697676316 | 0.038 | State transition |
| 129 | Q6R2V6 | CAPP2 | Phosphoenolpyruvate carboxylase 2 | 0.697676316 | 0.005 | Carbon fixation, Tricarboxylic acid cycle |
| 130 | A8IW34 | PURA | Adenylosuccinate synthetase, chloroplastic | 0.704688094 | 0.024 | "de novo" AMP biosynthetic process |
| 131 | Q7YKX3 | RR11 | 30S ribosomal protein S11, chloroplastic | 0.704688094 | 0.004 | Translation |
| 132 | Q68RJ5 | IFT81 | Intraflagellar transport protein 81 | 0.71177032 | 0.012 | Transport of tubulin within the cilium |
| 133 | P31178 | GLE | Autolysin | 0.718923724 | 0.037 | Cell wall organization |
| 134 | A8HYU5 | METK | S-adenosylmethionine synthase | 0.726149042 | 0.002 | S-adenosylmethionine biosynthesis |
| 135 | P83564 | GPX1 | Glutathione peroxidase 1, mitochondrial | 0.726149042 | 0.596 | Arachidonic acid metabolic process |
| 136 | Q27YU7 | RSP5 | Flagellar radial spoke protein 5 | 0.733446954 | 0.002 | Regulation of flagellar bending |
| 137 | A8I9E8 | CFA45 | Cilia- and flagella-associated protein 45 | 0.733446954 | 0.038 | Cell motality |
| 138 | P46869 | FLA10 | Kinesin-like protein FLA10 | 0.733446954 | 0.072 | Intraciliary anterograde transport |
| 139 | A1JHN0 | HSTC | Homogentisate solanesyltransferase | 0.733446954 | 0.027 | Carotenoid and PQ biosynthetic process |
| 140 | A8JAM0 | DRC7 | Dynein regulatory complex subunit 7 | 0.740818212 | 0.012 | Regulation of flagellar motility |
| 141 | P11660 | RTL | Reverse transcriptase-like protein | 0.740818212 | 0.243 | cDNA synthethesis |
| 142 | A8IH47 | CFA91 | Cilia- and flagella-associated protein 91 | 0.748263574 | 0.002 | Cilium movement |
| 143 | A8J6X7 | PCDP1 | Cilia- and flagella-associated protein 221 | 0.748263574 | 0.035 | Cilium assembly and movement |
| 144 | A8JID5 | CF157 | Cilia- and flagella-associated protein 157 | 0.755783741 | 0.013 | Cilium movement, Cell motility |
| 145 | Q8HTL3 | RK23 | 50S ribosomal protein L23, chloroplastic | 0.755783741 | 0.330 | Translation, Large subunit assembly |
| 146 | A0A2K3D5Z7 | CMD1 | 5-methylcytosine-modifying enzyme 1 | 0.763379486 | 0.003 | 5-methylcytosine catabolic process |
| 147 | Q39591 | DYL4 | Dynein 14 kDa light chain | 0.763379486 | 0.688 | Outer dynein arm assembly |
| 148 | Q39582 | TBG | Tubulin gamma chain | 0.763379486 | 0.063 | Cytoplasmic microtubule organization |
| 149 | Q8S339 | ALB31 | ALBINO3-like protein 1, chloroplastic | 0.771051593 | 0.021 | Assembly and activity of LHC I and II |
| 150 | A8I4E9 | CP100 | Cilia- and flagella-associated protein 100 | 0.771051593 | 0.951 | Cell motality, Cillium movement |
| 151 | Q9LD46 | CRD1 | Mg-protoporphyrin IX monomethyl ester | 0.771051593 | 0.020 | Photosynthesis, Chl biosynthetic process |
| 152 | Q9FEC4 | RAA3 | Trans-splicing factor Raa3, chloroplastic | 0.771051593 | 0.197 | mRNA processing, RNA splicing |
| 153 | A0A2K3DDJ2 | POB15 | Proteome of basal body protein 15 | 0.778800783 | 0.039 | Motility and sensing stimuli |
| 154 | A8ILK1 | CFA52 | Cilia- and flagella-associated protein 52 | 0.778800783 | 0.029 | Cell motility |
| 155 | P46870 | KLP1 | Kinesin-like protein KLP1 | 0.778800783 | 0.121 | Microtubule-based movement |
| 156 | A8HYP5 | IFT43 | Intraflagellar transport protein 43 | 0.778800783 | 0.004 | Cell projection organization |
| 157 | P25840 | HSP70 | Heat shock 70 kDa protein | 0.778800783 | 0.887 | Protein maturation and degradation |
| 158 | P23577 | CYF | Cytochrome f | 0.778800783 | 0.062 | CEF around PSI |
| 159 | P46284 | S17P | Sedoheptulose bisphosphatase, chloroplastic | 0.778800783 | 0.039 | Starch and Sucrose biosynthetic process |
| 160 | Q39578 | DYI2 | Dynein, 78 kDa intermediate chain | 0.786627865 | 0.053 | Motor activity, Arm assembly |
| 161 | P12852 | PSBQ | Oxygen-evolving enhancer 3, chloroplastic | 0.794533599 | 0.027 | Photosynthesis |
| 162 | O47027 | RR2A | 30S ribosomal protein S2, chloroplastic | 0.794533599 | 0.695 | Translation |
| 163 | Q8VXP3 | TBC2 | Tbc2 translation factor, chloroplastic | 0.802518799 | 0.015 | PsbC mRNA for translation initiation |
| 164 | Q39615 | PSAD | PS I reaction center subunit II, chloroplastic | 0.810584251 | 0.065 | Photosynthesis, Ferredoxin-docking site |
| 165 | O20031 | YCF3 | Photosystem I assembly protein Ycf3 | 0.810584251 | 0.333 | Chaperone-like assembly of the PSI |
| 166 | P22675 | ARLY | Argininosuccinate lyase | 0.818730751 | 0.003 | Arginine biosynthetic via ornithine |
| 167 | Q39604 | IDLC | 28 kDa inner dynein arm light chain | 0.818730751 | 0.226 | Cilium involved in cell motility |
| 168 | A8IYS6 | CF300 | Cilia- and flagella-associated protein 300 | 0.826959136 | 0.022 | Structure organization and motility |
| 169 | A8IRJ7 | CFA53 | Cilia- and flagella-associated protein 53 | 0.826959136 | 0.001 | Cilium assembly and movement |
| 170 | A8JAN3 | POC16 | Centriole proteome protein 16 | 0.835270205 | 0.082 | Flagellum assembly and maintenance |
| 171 | P10898 | PSBC | Photosystem II CP43 reaction center protein | 0.835270205 | 0.009 | PET in photosystem II |
| 172 | A8IHV3 | SLT3 | Probable sodium/sulfate cotransporter 3 | 0.835270205 | 0.025 | Sulfate transport |
| 173 | A8HNV0 | RSP14 | Radial spoke protein 14 | 0.843664815 | 0.003 | Cell motility, Endocytosis |
| 174 | P17746 | EFTU | Elongation factor Tu, chloroplastic | 0.843664815 | 0.056 | Translational elongation |
| 175 | P81831 | CAPP1 | Phosphoenolpyruvate carboxylase 1 | 0.843664815 | 0.021 | Carboxylation of PEP into oxaloacetate |
| 176 | P52908 | 1433 | 14-3-3-like protein | 0.843664815 | 0.451 | Flagellar arrangement and assembly |
| 177 | Q42695 | RUBC | RuBisCO large subunit- beta-2 | 0.860707971 | 0.067 | RuBisCO assembly, Protein refolding, |
| 178 | Q7XJ96 | DRC4 | Dynein regulatory complex subunit 4 | 0.869358235 | 0.012 | Axonemal dynein complex assembly |
| 179 | A8IQT2 | CCD40 | Coiled-coil domain-containing protein 40 | 0.878095435 | 0.023 | Assembly of dynein regulatory complex |
| 180 | Q9ZWM5 | CAO | Chlorophyllide a oxygenase, chloroplastic | 0.878095435 | 0.811 | Chlorophyll b biosynthetic process |
| 181 | A8J0N6 | DRC10 | Dynein regulatory complex protein 10 | 0.886920439 | 0.200 | Maintains and regulates microtubule |
| 182 | P07839 | FER | Ferredoxin, chloroplastic | 0.904837417 | 0.003 | Fe-S protein for metabolic reactions |
| 183 | B8LIX8 | IFT25 | Intraflagellar transport protein 25 | 0.904837417 | 0.090 | Intraciliary transport |
| 184 | Q8LKI3 | ALB3.2 | ALBINO3-like protein 2, chloroplastic | 0.913931182 | 0.008 | Assembly and activity of LHC I and II. |
| 185 | Q6RCE1 | IFT74 | Intraflagellar transport protein 74 | 0.913931182 | 0.001 | Cilium assembly |
| 186 | P37824 | CHLB | Light-independent protochlorophyllide | 0.913931182 | 0.913 | Light-independent chl biosynthetic |
| 187 | P05724 | YCX3 | 14.4 kDa protein in 16S rRNA region | 0.923116348 | 0.015 | Determine phylogeny |
| 188 | A8JFU2 | CFA65 | Cilia- and flagella-associated protein 65 | 0.93239382 | 0.019 | Cell projection organization |
| 189 | Q39571 | YPTC1 | GTP-binding protein YPTC1 | 0.93239382 | 0.003 | Protein transport, vesicular traffic |
| 190 | A8JAF2 | CFA43_ | Cilia- and flagella-associated protein 43 | 0.941764535 | 0.050 | Flagellum axoneme organization |
| 191 | Q949J1 | IPYR2 | Soluble inorganic pyrophosphatase 2 | 0.941764535 | 0.010 | Phosphate-containing metabolic process |
| 192 | A8J8F6 | TEKT | Tektin | 0.951229424 | 0.080 | Assembly of flagella and cilia |
| 193 | A0A2K3DZC4 | BSD2 | BUNDLE SHEATH DEFECTIVE 2, chloroplastic | 0.951229424 | 0.503 | Chaperone-mediated protein folding |
| 194 | Q5DM57 | IF172 | Intraflagellar transport protein 172 | 0.96078944 | 0.070 | Multicellular organism development |
| 195 | A8I2V9 | LISC | Lipoyl synthase, chloroplastic | 0.990049834 | 0.072 | Protein lipoylation |
| 196 | P51821 | ARF1 | ADP-ribosylation factor 1 | 0.990049834 | 0.005 | Vesicle-mediated transport |
| 197 | A8IHT2 | DRC11 | Dynein regulatory complex protein 11 | 0.990049834 | 0.711 | Regulator of ciliary/flagellar motility |
| 198 | P37823 | CHLB | Light-independent protochlorophyllide | 0.990049834 | 0.022 | Light-independent chl biosynthesis |
| 199 | A8IJF8 | SLT1 | Sodium/sulfate cotransporter 1 | 1 | 0.709 | Potassium ion transport |
| 200 | P42380 | CLPP | ATP-dependent Clp protease proteolytic | 1.02020134 | 0.271 | chymotrypsincontrol misfolded proteins |
| 201 | A8HS48 | RS3A | 40S ribosomal protein S3a | 1.02020134 | 0.110 | Translation |
| 202 | Q39584 | DYL3 | Dynein 18 kDa light chain | 1.030454533 | 0.013 | Motor activity, Calcium ion binding |
| 203 | A8IVX2 | DRC3 | Dynein regulatory complex subunit 3 | 1.040810773 | 0.009 | Cilium-dependent cell motility |
| 204 | A8IXB8 | ASNA2 | ATPase ARSA2 | 1.061836545 | 0.081 | Tail-anchored protein insertion into ER |
| 205 | Q8LPD9 | PHOT | Phototropin | 1.061836545 | 0.007 | Blue light signalling, NPQ induction |
| 206 | P12759 | RSP3 | Flagellar radial spoke protein 3 | 1.083287066 | 0.011 | Cilium-dependent cell motility |
| 207 | Q8HTL2 | RK2 | 50S ribosomal protein L2, chloroplastic | 1.083287066 | 0.001 | Mitochondrial translation |
| 208 | Q8HTL1 | RK5 | 50S ribosomal protein L5, chloroplastic | 1.094174288 | 0.005 | Translation |
| 209 | P09144 | PSAB | PSI P700 chlorophyll a apoprotein A2 | 1.10517092 | 0.050 | Primery electron donor of PSI |
| 210 | P29683 | FLA10 | Light-independent protochlorophyllide | 1.127496849 | 0.001 | Light-independent chl biosynthetic |
| 211 | Q8GTZ9 | CCS1 | Cytochrome c biogenesis, chloroplastic | 1.138828378 | 0.011 | Cytochrome complex assembly |
| 212 | P50884 | RL12 | 60S ribosomal protein L12 | 1.138828378 | 0.003 | Translation |
| 213 | A8HQ54 | DRC9 | Dynein regulatory complex protein 9 | 1.138828378 | 0.970 | Regulator of ciliary/flagellar motility |
| 214 | Q39566 | GSA | Glutamate semialdehyde aminomutase | 1.138828378 | 0.171 | Chlorophyll biosynthetic process |
| 215 | Q7XA07 | DC2L | Cytoplasmic dynein 2 intermediate chain 1 | 1.173510867 | 0.374 | Regulates flagellar dynein activity |
| 216 | P27080 | ADT | ADP,ATP carrier protein | 1.173510867 | 0.053 | Catalyzes the exchange of ADP and ATP |
| 217 | Q08356 | EZY3 | Ezy-1 protein | 1.197217372 | 0.064 | Uncharacterized protein |
| 218 | Q42689 | GLNA2 | Glutamine synthetase, chloroplastic | 1.221402762 | 0.009 | Glutamine biosynthetic process |
| 219 | Q93Y52 | IPYR1 | Soluble inorganic pyrophosphatase 1, | 1.221402762 | 0.790 | Phosphate-containing metabolism |
| 220 | A8JB22 | DRC2 | Dynein regulatory complex subunit 2 | 1.221402762 | 0.010 | Axonemal dynein complex assembly |
| 221 | Q27YU0 | RSP1 | Flagellar radial spoke protein 1 | 1.246076729 | 0.002 | Flagellar bending |
| 222 | P19824 | KPPR | Phosphoribulokinase, chloroplastic | 1.246076729 | 0.031 | Reductive pentose-phosphate cycle |
| 223 | Q00471 | CYB6 | Cytochrome b6 | 1.284025417 | 0.003 | CEF around PSI and state transitions. |
| 224 | O20030 | YCF4 | Photosystem I assembly protein Ycf4 | 1.284025417 | 0.479 | Assembly of the photosystem I complex |
| 225 | P06007 | PSBD | Photosystem II D2 protein | 1.296930074 | 0.957 | Assembly of PSII complex |
| 226 | Q94EY1 | PR46B | Coiled-coil domain-containing protein 103 | 1.309964465 | 0.334 | Cilia motility |
| 227 | P14217 | ARS | Arylsulfatase | 1.323129814 | 0.039 | Phenol-containing metabolic process |
| 228 | P14273 | CB2 | Chl a-b binding, LHCII type I, chloroplastic | 1.323129814 | 0.037 | Light-harvesting as a light receptor |
| 230 | Q00914 | PSAC | Photosystem I iron-sulfur center | 1.377127754 | 0.126 | Binding of PsaD and PsaE to PSI |
| 231 | A8JF71 | CTU1 | Cytoplasmic tRNA 2-thiolation protein 1 | 1.377127754 | 0.003 | Protein urmylation |
| 232 | A8JA42 | IFT56 | Intraflagellar transport protein 56 | 1.390968147 | 0.006 | Intraciliary transport |
| 233 | Q42688 | GLNA1 | Glutamine synthetase cytosolic isozyme | 1.390968147 | 0.002 | Glutamine biosynthetic process |
| 234 | P54347 | H2B4 | Histone H2B.4 | 1.404947596 | 0.781 | Protein heterodimerization activity |
| 235 | I2CYZ4 | D4FAD | Acyl-lipid (7-3)-desaturase, chloroplastic | 1.41906754 | 0.013 | Unsaturated and fatty acid biosynthetic |
| 236 | P12154 | PSAA | PSI P700 chlorophyll a apoprotein A1 | 1.41906754 | 0.464 | Photosynthesis |
| 237 | P12352 | PSAE | PSI reaction center subunit IV, chloroplastic | 1.433329435 | 0.047 | Photosynthesis |
| 238 | P07753 | PSBA | Photosystem II protein D1 | 1.447734622 | 0.060 | Photosynthetic electron transport in PSII |
| 239 | Q9SMH4 | RAA2 | Trans-splicing factor Raa2, chloroplastic | 1.462284582 | 0.133 | RNA splicing, mRNA processing |
| 240 | Q94EY2 | UFM1 | Ubiquitin-fold modifier 1 | 1.476980773 | 0.527 | Protein ufmylation |
| 241 | O48513 | RL13 | 60S ribosomal protein L13 | 1.476980773 | 0.258 | Translation |
| 242 | D2K6F1 | SLT2 | Sodium/sulfate cotransporter 2 | 1.537257535 | 0.034 | Potassium ion transport |
| 243 | P54346 | H2B3 | Histone H2B.3 | 1.537257535 | 0.704 | DNA binding |
| 244 | P08739 | NU5M | NADH-ubiquinone oxidoreductase chain 5 | 1.537257535 | 0.689 | ATP synthesis |
| 245 | P14224 | PSAG | Photosystem I RC subunit V, chloroplastic | 1.599994191 | 0.116 | Photosynthesis |
| 246 | A8HMZ4 | DRC5 | Dynein regulatory complex subunit 5 | 1.632316236 | 0.249 | Ciliary/flagellar motility |
| 247 | Q08365 | RR3 | 30S ribosomal protein S3, chloroplastic | 1.648721271 | 0.073 | Translational |
| 248 | Q8HUH0 | RPC2B | DNA-directed RNA polymerase subunit beta | 1.665291179 | 0.006 | Transcription, DNA-templated |
| 249 | Q01657 | RSP6 | Flagellar radial spoke protein 6 | 1.69893226 | 0.023 | Cilium assembly |
| 250 | P06541 | ATPB | ATP synthase subunit beta, chloroplastic | 1.750672504 | 0.195 | ATP synthesis coupled proton transport |
| 251 | Q39573 | YPTC5 | GTP-binding protein YPTC5 | 1.768267039 | 0.005 | GTPase activity, protein transport |
| 252 | P26526 | ATPA | ATP synthase subunit alpha, chloroplastic | 1.786038401 | 0.347 | ATP synthesis coupled proton transport |
| 253 | A8ITB0 | MOC1 | Holliday junction resolvase, chloroplastic | 1.803988368 | 0.115 | DNA recombination |
| 254 | P08197 | CYC6 | Cytochrome c6, chloroplastic | 1.803988368 | 0.256 | Electron carrier between cyt b_6_f and PSI |
| 255 | A8HPM2 | PSBS1 | Photosystem II protein PSBS1 | 1.822118844 | 0.006 | Nonphotochemical quenching |
| 256 | A0A2K3DMP5 | PSBR | Photosystem II protein PSBR, chloroplastic | 1.896480852 | 0.003 | Energy-dependent quenching-NPQ |
| 257 | A8HPM5 | PSBS2 | Photosystem II protein PSBS2 | 1.896480852 | 0.080 | Nonphotochemical quenching |
| 258 | P36495 | YCF78 | membrane protein ycf78 | 1.915540783 | 0.036 | Cell growth |
| 259 | Q42682 | HEM2 | Aminolevulinic acid dehydratase | 1.934792385 | 0.096 | Chlorophyll biosynthetic process |
| 260 | Q9FPQ6 | GP1 | Vegetative cell wall protein gp1 | 1.934792385 | 0.127 | component of the outer cell wall W6 |
| 261 | A8IGK2 | EFTS | Elongation factor Ts, mitochondrial | 1.934792385 | 0.081 | Translation elongation factor activity |
|  |  |  |  |  |  |  |
|  |  |  | **Up-regulated proteins ( FC ≥ 2.0)** |  |  |  |
|  |  |  |  |  |  |  |
| 262 | P11471 | PSBP | Oxygen-evolving enhancer 2, chloroplastic | 2.075080647 | 0.255 | Photosynthesis, Oxygen evolution |
| 263 | Q2HWK7 | DES | Acyl-lipid omega-13 desaturase | 2.1382762 | 0.501 | Linoleic acid metabolic process |
| 264 | H3JU05 | SRGT1 | Peptidyl serine alpha-galactosyltransferase | 2.247907992 | 0.052 | Transferring glycosyl groups |
| 265 | Q8HUH1 | RR2B | Putative 30S ribosomal S2-like protein | 2.247907992 | 0.001 | Transferase activity |
| 266 | Q3Y8L7 | DAW1 | Dynein assembly WDR repeat domains 1 | 2.611696417 | 0.120 | Intraciliary transport and assembly |
| 267 | P50881 | RL11 | 60S ribosomal protein L11 | 2.664456293 | 0.073 | Translation |
| 268 | Q39617 | POR | Protochlorophyllide reductase, chloroplastic | 2.773194711 | 0.051 | Chlorophyll biosynthetic process |
| 269 | P36437 | CHLB | Light-independent protochlorophyllide | 2.773194711 | 0.011 | Light-independent chl biosynthesis |
| 270 | P11658 | NU1M | NADH-ubiquinone oxidoreductase chain 1 | 3.034358438 | 0.002 | NADH dehydrogenase activity |
| 271 | P45841 | RL31 | 60S ribosomal protein L31 | 3.632786417 | 0.450 | Translation |
| 272 | Q93WD2 | CB29 | Chlorophyll a-b binding protein CP29 | 3.78104355 | 0.001 | LHC Facilitates State 1 to 2 transition |
| 273 | Q6QJE2 | SULP2 | Sulfate permease 2, chloroplastic | 3.935350714 | 0.028 | Sulfate transport |
| 274 | P11094 | RK14 | 50S ribosomal protein L14, chloroplastic | 4.05519987 | 0.045 | Translation |
| 275 | P12853 | PSBO | Oxygen-evolving enhancer1, chloroplastic | 4.137120263 | 0.003 | Primary site of water splitting |
| 276 | Q8RVC7 | SULP1 | Sulfate permease 1, chloroplastic | 4.85495602 | 0.605 | Uptake and assimilation of sulfate |
| 277 | A8J0J0 | LPAT | Acyl-sn-glycerol-3-phosphate acyltransferase | 7.53832479 | 0.007 | CDP-diacylglycerol biosynthesis |
| 278 | Q84U21 | RK22 | 50S ribosomal protein L22 | 38.09184072 | 0.027 | Translation |
| 279 | P49213 | RL44 | 60S ribosomal protein L44 | 41.26439529 | 0.607 | Translation |
| 280 | A8JHD7 | DRC6 | Dynein regulatory complex subunit 6 | 278.6621495 | 0.172 | Regulator of ciliary/flagellar motility |
|  |  |  |  |  |  |  |
|  |  |  | **Treated specific protein** |  |  |  |
|  |  |  |  |  |  |  |
| 281 | P05727 | RK16 | 50S ribosomal protein L16, chloroplastic | Treated_P500 |  | Mitochondrial translation |
| 282 | P05726 | RK16 | 50S ribosomal protein L16, chloroplastic | Treated _P500 |  | Mitochondrial translation |
| 283 | Q37304 | atpH | ATP synthase subunit c, chloroplastic | Treated_P500 |  | ATP synthesis coupled proton transport |
| 284 | P59777 | PSAJ | Photosystem I reaction center subunit IX | Treated_P500 |  | Organization of the PsaE and PsaF subunit |
| 285 | P59776 | RR19 | 30S ribosomal protein S19, chloroplastic | Treated_P500 |  | Translation |
| 286 | P59775 | RR8 | 30S ribosomal protein S8, chloroplastic | Treated_P500 |  | Translation |
| 287 | Q42684 | SODM | Superoxide dismutase [Mn], mitochondrial | Treated_P500 |  | Destroys superoxide anion radicals |
| 288 | Q42681 | H31 | Histone H3 type 1 | Treated_P500 |  | DNA binding, Chromosome stability |
| 289 | P10329 | ND6 | NADH-ubiquinone oxidoreductase chain 6 | Treated_P500 |  | NADH dehydrogenase activity |
| 290 | A8JJB2 | MOC2A | Molybdopterin synthase sulfur carrier | Treated_P500 |  | Mo-molybdopterin cofactor biosynthesis |
| 291 | O19930 | PSBJ | Photosystem II reaction center protein J | Treated_P500 |  | Photosynthesis, Charge separation |
| 292 | P50567 | H2A | Histone H2A | Treated_P500 |  | Chromosomal stability |
| 293 | P50566 | H4 | Histone H4 | Treated_P500 |  | Nucleosome remodelling |
| 294 | P50564 | H32 | Histone H3 type 3 | Treated_P500 |  | Post-translational modifications |
| 295 | P37256 | PSBT | Photosystem II reaction center protein T | Treated_P500 |  | Photosynthesis, Dimerization of PSII |
| 296 | Q9GGE2 | RR14 | 30S ribosomal protein S14, chloroplastic | Treated_P500 |  | Translation |
| 297 | P48269 | CCSA | Cytochrome c biogenesis protein CcsA | Treated_P500 |  | cytochrome complex assembly |
| 298 | P48268 | PSBE | Cytochrome b559 subunit alpha | Treated_P500 |  | PETC, Charge separation |
| 299 | A8IC48 | URM1 | Ubiquitin-related modifier 1 homolog | Treated_P500 |  | Endonuclease activity, Intron homing |
| 300 | A8HN58 | IFT27 | Intraflagellar transport protein 27 | Treated_P500 |  | GTPase activity, Form IFT complex B |
| 301 | P26565 | RK20 | 50S ribosomal protein L20, chloroplastic | Treated_P500 |  | Translation |
| 302 | P08740 | NU2M | NADH-ubiquinone oxidoreductase chain 2 | Treated_P500 |  | NADH dehydrogenase activity |
| 303 | P08681 | COX1 | Cytochrome c oxidase subunit 1 | Treated_P500 |  | Oxidative phosphorylation |
| 304 | P18263 | PSBK | Photosystem II reaction center protein K | Treated_P500 |  | Photosynthesis |
| 305 | Q06480 | PSBN | Protein PsbN | Treated_P500 |  | Role in photosystem I and II biogenesis |
| 306 | Q39580 | DYL1 | Dynein 8 kDa light chain | Treated_P500 |  | Microtubule-based process |
| 307 | Q6LCW8 | H32 | Histone H3 type 2 | Treated_P500 |  | Chromosomal stability |
| 308 | P32974 | PSBL | Photosystem II reaction center protein L | Treated_P500 |  | PPSII assembly and dimerization. |
| 309 | P47903 | RS27 | 40S ribosomal protein S27 | Treated_P500 |  | Microtubule-based process |
| 310 | P36443 | YCX6 | 12.3 kDa petA-petD intergenic region | Treated_P500 |  | chloroplasts based function |
| 311 | Q9SPI9 | PSBW | PSII reaction center W protein, chloroplastic | Treated_P500 |  | Stabilizes dimeric photosystem II |
| 312 | A8JGF7 | LIAS | Lipoyl synthase, mitochondrial | Treated_P500 |  | Lipoylation peptidyl-N6-lipoyl-L-lysine. |
| 313 | P23230 | PETD | Cytochrome b6-f complex subunit 4 | Treated_P500 |  | Transferring electrons within the CET |
| 314 | P37836 | PFL | Formate acetyltransferase | Treated_P500 |  | Form pyruvate and coA |
| 315 | Q9XF62 | DIP13 | 13 kDa deflagellation-inducible protein | Treated_P500 |  | Protein stability |
| 316 | P37825 | YCX5 | Uncharacterized trnR-chlB intergenic region | Treated_P500 |  | Tag-protein stability |
| 317 | P46295 | RS14 | 40S ribosomal protein S14 | Treated_P500 |  | Translation |
| 318 | O20032 | RR18 | 30S ribosomal protein S18, chloroplastic | Treated_P500 |  | Translation |
|  |  |  |  |  |  |  |
|  |  |  | **Control specific protein** |  |  |  |
|  |  |  |  |  |  |  |
| 319 | O63075 | ATPI | ATP synthase subunit a, chloroplastic | UOH_W_50 |  | ATP synthesis coupled proton transport |

**Table S3** List of proteins expressed in *pgr5*_500 vs WT_50 (treated vs control) of *C. reinhardtii*

| **S.No.** | **Accession** | **Abbreviation** | **Description** | **Fold Change** | **P-value** | **GO Description** |
| --- | --- | --- | --- | --- | --- | --- |
|  |  |  |  |  |  |  |
|  |  |  | **Down-regulated protein (FC ≤ 0.5)** |  |  |  |
|  |  |  |  |  |  |  |
| 1 | Q00469 | CHLL | Light-independent protochlorophyllide | 0.013568556 | 0.010 | Photosynthesis, Dark reaction |
| 2 | P11094 | RK14 | 50S ribosomal protein L14, chloroplastic | 0.298197268 | 0.012 | Translation |
| 3 | Q949J1 | IPYR2 | Soluble inorganic pyrophosphatase 2 | 0.343008499 | 0.010 | Phosphate-containing metabolic process |
| 4 | P12853 | PSBO | Oxygen-evolving enhancer 1, chloroplastic | 0.382892894 | 0.007 | Primary site of water splitting |
| 5 | P12154 | PSAA | PSI P700 chlorophyll a apoprotein A1 | 0.382892894 | 0.399 | Photosynthesis |
| 6 | Q94EY1 | PR46B | Coiled-coil domain-containing protein | 0.390627836 | 0.005 | Cilia motility |
| 7 | P08197 | CYC6 | Cytochrome c6, chloroplastic | 0.390627836 | 0.184 | Electron carrier between cyt b6-f and PSI |
| 8 | B8LIX8 | IFT25 | Intraflagellar transport protein 25 | 0.410655759 | 0.034 | Intraciliary transport |
| 9 | Q9FEC4 | RAA3 | Trans-splicing factor Raa3, chloroplastic | 0.418951547 | 0.324 | mRNA processing, RNA splicing |
| 10 | Q42693 | RUBB | RuBisCO large subunit-protein beta-1 | 0.427414922 | 0.162 | Assembly of the enzyme oligomer |
| 11 | P49213 | RL44 | 60S ribosomal protein L44 | 0.440431658 | 0.273 | Translation, Response to cycloheximide |
| 12 | P14273 | CB2 | Chl a-b binding protein, LHCII type I | 0.440431658 | 0.415 | Photosynthesis, LHC as light receptor |
| 13 | Q8HUH1 | RR2B | Putative 30S ribosomal S2-like protein | 0.458406024 | 0.234 | Translation |
| 14 | P50881 | RL11 | 60S ribosomal protein L11 | 0.463013077 | 0.053 | Translation |
| 15 | Q37050 | CEMA | Chloroplast envelope membrane protein | 0.463013077 | 0.056 | Proton transporter activity |
| 16 | O63075 | ATPI | ATP synthase subunit a, chloroplastic | 0.467666431 | 0.567 | ATP synthesis coupled proton transport |
| 17 | Q8GTZ9 | CCS1 | Cytochrome c biogenesis protein | 0.472366553 | 0.392 | Cytochrome complex assembly |
| 18 | P49202 | RS18 | 40S ribosomal protein S18 | 0.481908981 | 0.006 | Translation |
| 19 | H3JU05 | SRGT1 | Peptidyl serine-galactosyltransferase | 0.486752242 | 0.426 | Transferase activity |
| 20 | P50564 | H33 | Histone H3 type 3 | 0.491644208 | 0.586 | Nucleosome remodelling |
| 21 | Q42695 | RUBC | RuBisCO large subunit- beta-2 | 0.49658531 | 0.431 | RuBisCO assembly, Protein refolding, |
| 22 | Q6LCW8 | H32 | Histone H3 type 2 | 0.49658531 | 0.015 | Chromosomal stability. |
|  |  |  |  |  |  |  |
|  |  |  | **Differential regulated protein (≤ 2.0 FC ≥0.5)** |  |  |  |
|  |  |  |  |  |  |  |
| 23 | Q9LD46 | CRD1 | Mg-protoporphyrin IX monomethyl ester | 0.511708569 | 0.062 | Photosynthesis, Chl biosynthetic process |
| 24 | O47027 | RR2A | 30S ribosomal protein S2, chloroplastic | 0.532591804 | 0.296 | Translation |
| 25 | P14224 | PSAG | Photosystem I RC subunit V, chloroplastic | 0.543350861 | 0.647 | Photosynthesis |
| 26 | Q39604 | IDLC | 28 kDa inner dynein arm light chain | 0.543350861 | 0.474 | Cilium movement involved in cell motility |
| 27 | P46284 | S17P | Sedoheptulose-1,7-bisphosphatase | 0.548811623 | 0.003 | Starch and Sucrose biosynthetic process |
| 28 | P0CH11 | RL401 | Ubiquitin-60S ribosomal protein L40 | 0.554327299 | 0.267 | Translation |
| 29 | P14149 | RR12 | 30S ribosomal protein S12, chloroplastic | 0.554327299 | 0.086 | Translation |
| 30 | A8HYU5 | METK | S-adenosylmethionine synthase | 0.554327299 | 0.413 | S-adenosylmethionine biosynthesis |
| 31 | A8IGK2 | EFTS | Elongation factor Ts, mitochondrial | 0.559898376 | 0.019 | Translation elongation factor activity |
| 32 | Q93Y52 | IPYR1 | Soluble inorganic pyrophosphatase 1 | 0.571209062 | 0.176 | Phosphate-containing metabolic process |
| 33 | A8IYS6 | CF300 | Cilia- and flagella-associated protein 300 | 0.58274824 | 0.000 | Axonemal organization and motility |
| 34 | A8IHT2 | DRC11 | Dynein regulatory complex protein 11 | 0.58274824 | 0.384 | Regulator of ciliary/flagellar motility |
| 35 | P05725 | DNE1 | DNA endonuclease I-CreI | 0.594520559 | 0.915 | Endonuclease activity, Intron homing |
| 36 | A8HN58 | IFT27 | Intraflagellar transport protein 27 | 0.600495585 | 0.008 | GTPase activity, Form IFT complex B |
| 37 | Q8GSP8 | ZYS3 | Zygote-specific protein 3 | 0.600495585 | 0.181 | Cell development |
| 38 | A8J6J0 | SULT2 | Proton/sulfate cotransporter 2 | 0.600495585 | 0.092 | Sulfate transporter activity |
| 39 | P0CH10 | RL403 | Ubiquitin-60S ribosomal protein L40 | 0.618783398 | 0.304 | Translation |
| 40 | Q93WD2 | CB29 | Chlorophyll a-b binding protein CP29 | 0.625002269 | 0.046 | LHC Facilitates the State 1 to 2 transition |
| 41 | Q00914 | PSAC | Photosystem I iron-sulfur center | 0.637628159 | 0.032 | Required for binding of PsaD, PsaE to PSI |
| 42 | A8J9T5 | THI4 | Thiamine thiazole synthase, chloroplastic | 0.637628159 | 0.800 | Thiamine biosynthetic process |
| 43 | P59763 | PSBI | Photosystem II reaction center protein I | 0.644036423 | 0.007 | Stability and assembly of PSII |
| 44 | Q39608 | NRT21 | Nitrate transporter 2.1 | 0.644036423 | 0.177 | Nitrate assimilation |
| 45 | Q9SW75 | RL10A | 60S ribosomal protein L10a | 0.657046828 | 0.129 | Translation |
| 46 | Q2HWK7 | DES | Acyl-lipid omega-13 desaturase | 0.670320042 | 0.588 | Linoleic acid metabolic process |
| 47 | Q9GGE2 | RR14 | 30S ribosomal protein S14, chloroplastic | 0.677056884 | 0.094 | Translation |
| 48 | A8HYP5 | IFT43 | Intraflagellar transport protein 43 | 0.677056884 | 0.412 | Cell projection organization |
| 49 | A0A2K3D5Z7 | CMD1 | 5-methylcytosine-modifying enzyme 1 | 0.683861412 | 0.020 | 5-methylcytosine catabolic process |
| 50 | Q9XHH2 | DNAL1 | Dynein light chain 1, axonemal | 0.683861412 | 0.001 | Motor activity for flagellar motility |
| 51 | P27080 | ADT | ADP,ATP carrier protein | 0.690734327 | 0.635 | Catalyzes the exchange of ADP and ATP |
| 52 | P41758 | PGKH | Phosphoglycerate kinase, chloroplastic | 0.697676316 | 0.587 | Glycolytic process, Reductive PPP |
| 53 | P48269 | CCSA | Cytochrome c biogenesis protein CcsA | 0.697676316 | 0.001 | Cytochrome complex assembly |
| 54 | P53498 | ACT | Actin | 0.697676316 | 0.188 | Cytoplasmic streaming |
| 55 | O20031 | YCF3 | Photosystem I assembly protein Ycf3 | 0.704688094 | 0.218 | Chaperone-like assembly of PSI subunits |
| 56 | F5A894 | DAAF3 | Dynein assembly factor 3, axonemal | 0.71177032 | 0.098 | Axonemal dynein complex assembly |
| 57 | P46870 | KLP1 | Kinesin-like protein KLP1 | 0.718923724 | 0.434 | Microtubule-based movement |
| 58 | Q42690 | ALFC | Fructose-bisphosphate aldolase 1 | 0.726149042 | 0.181 | Glycolytic process |
| 59 | P0DO19 | LHR31 | LHC stress-related protein 3.1 | 0.726149042 | 0.240 | Nonphotochemical quenching |
| 60 | Q39578 | DYI2 | Dynein, 78 kDa intermediate chain | 0.726149042 | 0.021 | Motor activity, Arm assembly |
| 61 | P06541 | ATPB | ATP synthase subunit beta, chloroplastic | 0.726149042 | 0.181 | ATP synthesis coupled proton transport |
| 62 | P05724 | YCX3 | 14.4 kDa protein in 16S rRNA | 0.733446954 | 0.504 | Determine phylogeny |
| 63 | P12759 | RSP3 | Flagellar radial spoke protein 3 | 0.733446954 | 0.030 | Protein localization to organelle |
| 64 | A8IF44 | CFA61 | Cilia- and flagella-associated protein 61 | 0.733446954 | 0.123 | Cilium movement, Cell organization |
| 65 | A8JB22 | DRC2 | Dynein regulatory complex subunit 2 | 0.733446954 | 0.461 | Axonemal dynein complex assembly |
| 66 | P17746 | EFTU | Elongation factor Tu, chloroplastic | 0.740818212 | 0.236 | Translational elongation |
| 67 | Q6RCE1 | IFT74 | Intraflagellar transport protein 74 | 0.740818212 | 0.603 | Cilium assembly |
| 68 | P25840 | HSP70 | Heat shock 70 kDa protein | 0.740818212 | 0.039 | Protein maturation and degradation |
| 69 | P09205 | TBA2 | Tubulin alpha-2 chain | 0.748263574 | 0.348 | Alters the microtubule cytoskeleton |
| 70 | P09204 | TBA1 | Tubulin alpha-1 chain | 0.748263574 | 0.290 | Microtubule-based process |
| 71 | Q09JZ4 | DAAF1 | Leucine-rich repeat-containing protein | 0.748263574 | 0.727 | Cell motility and localization to organelle |
| 72 | I2CYZ4 | D4FAD | Acyl-lipid (7-3)-desaturase, chloroplastic | 0.748263574 | 0.141 | Unsaturated and fatty acid biosynthetic |
| 73 | Q7XJ96 | DRC4 | Dynein regulatory complex subunit 4 | 0.748263574 | 0.508 | Axonemal dynein complex assembly |
| 74 | A8J2Z9 | FEN1 | Flap endonuclease 1 | 0.755783741 | 0.314 | DNA replication, Base-excision repair |
| 75 | Q39566 | GSA | Glutamate semialdehyde aminomutase | 0.755783741 | 0.869 | Chlorophyll biosynthetic process |
| 76 | P50362 | G3PA | G3P-dehydrogenase A, chloroplastic | 0.755783741 | 0.713 | Glucose metabolic process |
| 77 | O48513 | RL13 | 60S ribosomal protein L13 | 0.755783741 | 0.495 | Translation |
| 78 | Q6UBQ3 | RSP2 | Flagellar radial spoke protein 2 | 0.763379486 | 0.028 | Flagellar bending |
| 79 | A8HQ54 | DRC9 | Dynein regulatory complex protein 9 | 0.763379486 | 0.373 | Regulator of ciliary/flagellar motility |
| 80 | Q8LPD9 | PHOT | Phototropin | 0.763379486 | 0.696 | Blue light signalling, NPQ induction |
| 81 | A8IQE0 | CCD39 | Coiled-coil domain-containing protein 39 | 0.771051593 | 0.592 | Cilium movement |
| 82 | A8J6X7 | PCDP1 | Cilia- and flagella-associated protein 221 | 0.771051593 | 0.170 | Cilium assembly and movement |
| 83 | A2T2X4 | IFT46 | Intraflagellar transport protein 46 | 0.771051593 | 0.013 | Cilium-dependent cell motility |
| 84 | A8IU92 | CFA20 | Cilia- and flagella-associated protein 20 | 0.771051593 | 0.094 | Axoneme, Cilium assembly |
| 85 | P23489 | RCA | RuBisCO/oxygenase activase, chloroplastic | 0.778800783 | 0.420 | Activation of RuBisCO |
| 86 | P12356 | PSAF | PSI RCsubunit III, chloroplastic | 0.786627865 | 0.069 | Electron transfer PC to P700 |
| 87 | P52908 | 1433 | 14-3-3-like protein | 0.794533599 | 0.155 | Flagellar arrangement and assembly |
| 88 | A8JAM0 | DRC7 | Dynein regulatory complex subunit 7 | 0.802518799 | 0.325 | Regulation of flagellar motility |
| 89 | P36495 | YCF78 | membrane protein ycf78 | 0.802518799 | 0.538 | Cell growth |
| 90 | A8ITB0 | MOC1 | Holliday junction resolvase, chloroplastic | 0.802518799 | 0.340 | DNA recombination |
| 91 | O20029 | RR9 | 30S ribosomal protein S9, chloroplastic | 0.810584251 | 0.308 | Translation |
| 92 | Q8HTL6 | RPOB1 | DNA-directed RNA polymerase beta | 0.810584251 | 0.385 | Transcription, DNA-templated |
| 93 | A8JF71 | CTU1 | Cytoplasmic tRNA 2-thiolation protein 1 | 0.818730751 | 0.005 | Protein urmylation |
| 94 | P20113 | NU4M | NADH-ubiquinone oxidoreductase chain 4 | 0.818730751 | 0.017 | Electrons NADH to the respiratory chain |
| 95 | P18068 | PLAS | Plastocyanin, chloroplastic | 0.835270205 | 0.827 | Electron transfer P700 and cyt b_6_f |
| 96 | P26565 | RK20 | 50S ribosomal protein L20, chloroplastic | 0.835270205 | 0.461 | Translation |
| 97 | A8JAN3 | POC16 | Centriole proteome protein 16 | 0.843664815 | 0.350 | Flagellum assembly and maintenance |
| 98 | A8HME3 | IFT22 | Intraflagellar transport protein 22 | 0.843664815 | 0.776 | Cellular availability of IFT particles |
| 99 | A8IW99 | PLD6 | Mitochondrial cardiolipin hydrolase | 0.843664815 | 0.224 | Lipid catabolic process |
| 100 | Q08365 | RR3 | 30S ribosomal protein S3, chloroplastic | 0.843664815 | 0.281 | Translation |
| 101 | P07891 | ATPE | ATP synthase epsilon chain, chloroplastic | 0.843664815 | 0.243 | ATP synthesis coupled proton transport |
| 102 | Q42694 | RUBA | RuBisCO large subunit-alpha, chloroplastic | 0.860707971 | 0.199 | Assembly of the enzyme oligomer |
| 103 | A8JID5 | CF157 | Cilia- and flagella-associated protein 157 | 0.860707971 | 0.016 | Cilium movement, Cell motility |
| 104 | P49728 | UCRIA | Cyt b6-f complex Fe-S subunit | 0.860707971 | 0.320 | Mediates LET and CET around PSI |
| 105 | P22675 | ARLY | Argininosuccinate lyase | 0.860707971 | 0.014 | Arginine biosynthetic via ornithine |
| 106 | Q9SMH4 | RAA2 | Trans-splicing factor Raa2, chloroplastic | 0.860707971 | 0.469 | RNA splicing, mRNA processing |
| 107 | P23577 | CYF | Cytochrome f | 0.860707971 | 0.211 | CEF around PSI |
| 108 | Q27YU0 | RSP1 | Flagellar radial spoke protein 1 | 0.869358235 | 0.831 | Flagellar bending |
| 109 | A8IVX2 | DRC3 | Dynein regulatory complex subunit 3 | 0.869358235 | 0.357 | Cilium-dependent cell motility |
| 110 | A8JAF2 | CFA43 | Cilia- and flagella-associated protein 43 | 0.869358235 | 0.588 | Flagellum organization and function |
| 111 | P0DO18 | LHR32 | LHC stress-related protein 3.2 | 0.869358235 | 0.192 | Nonphotochemical quenching |
| 112 | A8HPM5 | PSBS2 | Photosystem II protein PSBS2 | 0.869358235 | 0.649 | Nonphotochemical quenching |
| 113 | Q9LLC6 | PETO | Cyt b6f complex subunit, chloroplastic | 0.869358235 | 0.256 | LET between PSII and I, CETaround PSI |
| 114 | Q94EY2 | UFM1 | Ubiquitin-fold modifier 1 | 0.878095435 | 0.012 | Protein ufmylation |
| 115 | Q39617 | POR | Protochlorophyllide reductase | 0.878095435 | 0.602 | Chlorophyll biosynthetic process |
| 116 | A8HYJ1 | TOC34l | Translocase of chloroplast 34, chloroplastic | 0.878095435 | 0.058 | Protein from the cytoplasm to chloroplast |
| 117 | P06007 | PSBD | Photosystem II D2 protein | 0.886920439 | 0.053 | Photosynthetic, Assembly of PSII complex |
| 118 | Q5DM57 | IF172 | Intraflagellar transport protein 172 | 0.886920439 | 0.369 | Multicellular organism development |
| 119 | A8J0N6 | DRC10 | Dynein regulatory complex protein 10 | 0.886920439 | 0.102 | Regulates sliding in axonemes |
| 120 | Q39615 | PSAD | PSI RC subunit II, chloroplastic | 0.886920439 | 0.183 | Ferredoxin-docking protein |
| 121 | Q5QD03 | SUVH3 | Histone-lysine N-methyltransferase | 0.886920439 | 0.698 | Repressed chromatin functions |
| 122 | Q8HUH2 | RPOA | DNA-directed RNA polymerase alpha | 0.886920439 | 0.255 | Transcription, DNA-templated |
| 123 | A4GRC6 | HAP2 | Hapless 2 | 0.886920439 | 0.194 | Protein insertion into the membrane |
| 124 | A8ID74 | BOP1 | Ribosome biogenesis protein homolog | 0.895834136 | 0.116 | Maturation of 5.8S rRNA |
| 125 | P42380 | CLPP | ATP-dependent Clp protease proteolytic | 0.895834136 | 0.106 | chymotrypsin-control misfolded proteins |
| 126 | P0DL09 | DRC1 | Dynein regulatory complex protein 1 | 0.895834136 | 0.033 | Dynein complex assembly |
| 127 | P19824 | KPPR | Phosphoribulokinase, chloroplastic | 0.904837417 | 0.020 | Reductive pentose-phosphate cycle |
| 128 | Q8HUG9 | RPC1A | DNA-directed RNA polymerase beta | 0.904837417 | 0.958 | Transcription by RNA polymerase II |
| 129 | A8JGB0 | ASNA1 | ATPase ARSA1 | 0.904837417 | 0.461 | ATPase activity |
| 130 | P05722 | YCX2 | Protein in 16S rRNA region | 0.913931182 | 0.174 | Phylogenetic relationship |
| 131 | Q39584 | DYL3 | Dynein 18 kDa light chain | 0.913931182 | 0.013 | Motor activity, Calcium ion binding |
| 132 | Q6R2V6 | CAPP2 | Phosphoenolpyruvate carboxylase 2 | 0.913931182 | 0.116 | Carbon fixation, Tricarboxylic acid cycle |
| 133 | A0A2K3DDJ2 | POB15 | Proteome of basal body protein 15 | 0.923116348 | 0.089 | Motility and sensing stimuli |
| 134 | A8HUA1 | CFA58 | Cilia- and flagella-associated protein 58 | 0.923116348 | 0.248 | Cilium-dependent cell motility |
| 135 | A8HMZ4 | DRC5 | Dynein regulatory complex subunit 5 | 0.923116348 | 0.196 | Ciliary/flagellar motility |
| 136 | A8INQ0 | ARL13 | ADP-ribosylation factor-like protein 13B | 0.923116348 | 0.582 | Control ciliary axoneme structure |
| 137 | Q9ZWM5 | CAO | Chlorophyllide a oxygenase, chloroplastic | 0.923116348 | 0.097 | Chlorophyll b biosynthetic process |
| 138 | A8ILK1 | CFA52 | Cilia- and flagella-associated protein 52 | 0.93239382 | 0.112 | Cell motility |
| 139 | A8JFU2 | CFA65 | Cilia- and flagella-associated protein 65 | 0.93239382 | 0.423 | Cell projection organization |
| 140 | P93107 | PF20 | Flagellar WD repeat-containing Pf20 | 0.93239382 | 0.425 | Inter-microtubule bridges in flagella |
| 141 | A8IW34 | PURA | Adenylosuccinate synthetase, chloroplastic | 0.951229424 | 0.030 | "de novo" AMP biosynthetic process |
| 142 | A8J0J0 | LPAT | Acyl-sn-glycerol-phosphate acyltransferase | 0.951229424 | 0.228 | CDP-diacylglycerol biosynthetic process |
| 143 | P46869 | FLA10 | Kinesin-like protein FLA10 | 0.951229424 | 0.422 | Intraciliary anterograde transport |
| 144 | Q39570 | YPTC4 | GTP-binding protein YPTC4 | 0.96078944 | 0.254 | Protein transport and vesicular traffic |
| 145 | Q8VXP3 | TBC2 | Tbc2 translation factor, chloroplastic | 0.970445534 | 0.062 | PsbC mRNA for translation initiation |
| 146 | Q39586 | METE | Homocysteine methyltransferase | 0.970445534 | 0.518 | Methionine biosynthetic process |
| 147 | P14217 | ARS | Arylsulfatase | 0.980198674 | 0.100 | Phenol-containing metabolic process |
| 148 | Q84V18 | STT7 | Serine/threonine- kinase, chloroplastic | 0.980198674 | 0.864 | State transition |
| 149 | P13352 | PSAH | Photosystem I RC subunit VI, chloroplastic | 0.990049834 | 0.287 | Photosynthesis, Docking for LHC I |
| 150 | P51821 | ARF1 | ADP-ribosylation factor 1 | 0.990049834 | 0.105 | Vesicle-mediated transport |
| 151 | A8IUG5 | CFA99 | Cilia- and flagella-associated protein 99 | 1 | 0.777 | Ciliary/flagellar motility |
| 152 | P93664 | LHSR1 | LHC stress-related protein 1, chloroplastic | 1 | 0.163 | Non-photochemical quenching |
| 153 | P29763 | RLA1 | 60S acidic ribosomal protein P1 | 1 | 0.141 | Translational elongation |
| 154 | P81831 | CAPP1 | Phosphoenolpyruvate carboxylase 1 | 1 | 0.122 | Carboxylation of PEP into oxaloacetate |
| 155 | M1V4Y8 | CFA73 | Cilia- and flagella-associated protein 73 | 1 | 0.389 | Regulation of microtubule motor activity |
| 156 | P12113 | ATPG | ATP synthase gamma chain, chloroplastic | 1.010050167 | 0.003 | ATP synthesis coupled proton transport |
| 157 | Q7YKX3 | RR11 | 30S ribosomal protein S11, chloroplastic | 1.010050167 | 0.085 | Translation |
| 158 | Q42682 | HEM2 | Aminolevulinic dehydratase, chloroplastic | 1.02020134 | 0.561 | Chlorophyll biosynthetic process |
| 159 | P29683 | CHLN | Light-independent protochlorophyllide | 1.030454533 | 0.360 | Light-independent chl biosynthetic |
| 160 | Q8HUH0 | RPC2B | DNA-directed RNA polymerase beta | 1.030454533 | 0.304 | Transcription, DNA-templated |
| 161 | P11471 | PSBP | Oxygen-evolving enhancer 2, chloroplastic | 1.030454533 | 0.004 | Photosynthesis, Oxygen evolution |
| 162 | P48267 | RR7 | 30S ribosomal protein S7, chloroplastic | 1.040810773 | 0.319 | Translation |
| 163 | Q39593 | SAC1 | Putative sulfur deprivation regulator | 1.040810773 | 0.414 | Potassium ion transport |
| 164 | A8J1V4 | CFA44 | Cilia- and flagella-associated protein 44 | 1.040810773 | 0.138 | Cilium-dependent cell motility |
| 165 | P49644 | G3PC | G3P dehydrogenase, cytosolic | 1.051271097 | 0.234 | Carbohydrate metabolism |
| 166 | P31683 | ENO | Enolase (Fragment) | 1.061836545 | 0.334 | Glycolytic process |
| 167 | P36437 | CHLB | Light-independent protochlorophyllide | 1.061836545 | 0.372 | Light-independent chl biosynthetic |
| 168 | A1JHN0 | HSTC | Homogentisate solanesyltransferase | 1.061836545 | 0.007 | Carotenoid, Plastoquinone biosynthetic |
| 169 | A8IXB8 | ASNA2 | ATPase ARSA2 | 1.072508182 | 0.596 | Tail-anchored insertion ER membrane |
| 170 | A8JHD7 | DRC6 | Dynein regulatory complex subunit 6 | 1.072508182 | 0.339 | Regulator of ciliary/flagellar motility |
| 171 | P08475 | RBS2 | RuBP carboxylase small chain 2 | 1.083287066 | 0.894 | Photosynthesis, Carbon fixation |
| 172 | P80028 | TRXH | Thioredoxin H-type | 1.083287066 | 0.188 | Cell redox homeostasis |
| 173 | P23400 | TRXM | Thioredoxin M-type, chloroplastic | 1.094174288 | 0.026 | Cell redox homeostasis |
| 174 | Q39582 | TBG | Tubulin gamma chain | 1.094174288 | 0.119 | Cytoplasmic microtubule organization |
| 175 | A0A2K3DZC4 | BSD2 | BUNDLE SHEATH DEFECTIVE 2 | 1.094174288 | 0.711 | Chaperone-mediated protein folding |
| 176 | P00877 | RBL | RuBisCO carboxylase large chain | 1.094174288 | 0.029 | Carboxylation of RuBP in PPP |
| 177 | A8IHV3 | SLT3 | Probable sodium/sulfate cotransporter 3 | 1.10517092 | 0.106 | Sulfate transport |
| 178 | Q9XF62 | DIP13 | 13 kDa deflagellation-inducible protein | 1.10517092 | 0.059 | Protein stability |
| 179 | Q8HTL2 | RK2 | 50S ribosomal protein L2, chloroplastic | 1.11627807 | 0.727 | Mitochondrial translation |
| 180 | A8JBB2 | PESC | Pescadillo homolog | 1.127496849 | 0.809 | Maturation of 5.8S rRNA |
| 181 | Q9LEM8 | NAC2 | PsbD mRNA maturation factor | 1.127496849 | 0.648 | mRNA processing- 5' UTR of psbD mRNA |
| 182 | A8IQT2 | CCD40 | Coiled-coil domain-containing protein 40 | 1.127496849 | 0.389 | Assembly of dynein regulatory complex |
| 183 | P37836 | PFL | Formate acetyltransferase | 1.127496849 | 0.014 | Form pyruvate and coA |
| 184 | Q39618 | SFAS | SF-assemblin | 1.1502738 | 0.100 | Striated microtubule-associated assembly |
| 185 | P20507 | CAH1 | Carbonic anhydrase 1 | 1.1502738 | 0.330 | Carbonate dehydratase activity |
| 186 | A8IZG4 | CIAO1 | Probable cytosolic Fe -S protein assembly | 1.1502738 | 0.392 | Required iron-sulfur cluster assembly |
| 187 | Q6QJE2 | SULP2 | Sulfate permease 2, chloroplastic | 1.16183425 | 0.944 | Sulfate transport |
| 188 | A8ITV9 | CFA70 | Cilia- and flagella-associated protein 70 | 1.16183425 | 0.887 | Axoneme- regulates ciliary motility |
| 189 | P23230 | PETD | Cytochrome b6-f complex subunit 4 | 1.16183425 | 0.004 | Transferring electrons within the CET |
| 190 | Q42689 | GLNA2 | Glutamine synthetase, chloroplastic | 1.173510867 | 0.171 | Glutamine biosynthetic process |
| 191 | D4P3R7 | CFA74 | Cilia- and flagella-associated protein 74 | 1.173510867 | 0.327 | Cilium movement, Cell motility |
| 192 | Q39591 | CFA74 | Dynein 14 kDa light chain | 1.173510867 | 0.174 | Outer dynein arm assembly |
| 193 | A8IB22 | CFA77 | Cilia- and flagella-associated protein 77 | 1.173510867 | 0.722 | Cell motility |
| 194 | P24258 | CAH2 | Carbonic anhydrase 2 | 1.173510867 | 0.253 | CCM, Carbonate dehydratase activity |
| 195 | P37824 | CHLB | Light-independent protochlorophyllide | 1.173510867 | 0.379 | Light-independent chl biosynthetic |
| 196 | P12852 | PSBQ | Oxygen-evolving enhancer 3, chloroplastic | 1.185304853 | 0.369 | Photosynthesis |
| 197 | P12352 | PSAE | PSI RC subunit IV, chloroplastic | 1.185304853 | 0.241 | Photosynthesis |
| 198 | P37823 |  | Light-independent protochlorophyllide | 1.185304853 | 0.004 | Light-independent chlorophyll synthesis |
| 199 | Q42686 | MDHM | Malate dehydrogenase, mitochondrial | 1.197217372 | 0.099 | Carbohydrate metabolic process |
| 200 | P83564 | GPX1 | Glutathione peroxidase 1, mitochondrial | 1.197217372 | 0.104 | Arachidonic acid metabolic process |
| 201 | A8IRJ7 | CFA53 | Cilia- and flagella-associated protein 53 | 1.197217372 | 0.727 | Cilium assembly and movement |
| 202 | Q9STD3 | CALR | Calreticulin | 1.197217372 | 0.011 | Protein folding |
| 203 | A8IEF3 | ANM1 | Protein arginine N-methyltransferase 1 | 1.209249595 | 0.740 | Asymmetric dimethylation of a flagellum |
| 204 | A8IJF8 | SLT1 | Sodium/sulfate cotransporter 1 | 1.209249595 | 0.989 | Potassium ion transport |
| 205 | Q42688 | GLNA1 | Glutamine synthetase cytosolic isozyme | 1.221402762 | 0.370 | Glutamine biosynthetic process |
| 206 | Q39571 | YPTC1 | GTP-binding protein YPTC1 | 1.221402762 | 0.698 | Protein transport, vesicular traffic |
| 207 | P38482 | ATPBM | ATP synthase subunit beta, mitochondrial | 1.221402762 | 0.022 | ATP synthesis coupled proton transport |
| 208 | P50884 | RL12 | 60S ribosomal protein L12 | 1.233678052 | 0.237 | Translation |
| 209 | A8JGF7 | LIAS | Lipoyl synthase, mitochondrial | 1.246076729 | 0.230 | Lipoylation peptidyl-N6-lipoyl-L-lysine. |
| 210 | Q27YU7 | RSP5 | Flagellar radial spoke protein 5 | 1.258600015 | 0.010 | Regulation of flagellar bending |
| 211 | Q08354 | EZY1 | Ezy-1 protein | 1.258600015 | 0.384 | Uncharacterized protein |
| 212 | P31178 | GLE | Autolysin | 1.258600015 | 0.225 | Cell wall organization |
| 213 | A8HNV0 | RSP14 | Radial spoke protein 14 | 1.271249144 | 0.046 | Cell motility, Endocytosis |
| 214 | Q01656 | RSP4 | Flagellar radial spoke protein 4 | 1.271249144 | 0.272 | Cell motility, cilium assembly |
| 215 | D2K6F1 | SLT2 | Sodium/sulfate cotransporter 2 | 1.284025417 | 0.288 | Potassium ion transport |
| 216 | Q94FT3 | CHLI | Magnesium-chelatase subunit | 1.296930074 | 0.280 | Insertion of Mg into protoporphyrin ring |
| 217 | Q68RJ5 | IFT81 | Intraflagellar transport protein 81 | 1.309964465 | 0.268 | Transport of tubulin within the cilium |
| 218 | P50566 | H4 | Histone H4 | 1.309964465 | 0.218 | Nucleosome remodelling |
| 219 | P53991 | FENR | Ferredoxin--NADP reductase, chloroplastic | 1.309964465 | 0.102 | Regulating the cyclic and non-CEF |
| 220 | A8JA42 | IFT56 | Intraflagellar transport protein 56 | 1.323129814 | 0.479 | Intraciliary transport |
| 221 | Q7XA07 | DC2L | Cytoplasmic dynein 2 intermediate chain 1 | 1.323129814 | 0.152 | Regulates flagellar dynein activity |
| 222 | Q3Y8L7 | DAW1 | Dynein assembly WDR repeat domains 1 | 1.336427477 | 0.053 | Intraciliary transport and assembly |
| 223 | A8IH47 | CFA91 | Cilia- and flagella-associated protein 91 | 1.336427477 | 0.681 | Cilium movement |
| 224 | P00873 | RBS1 | RuBisCO small chain 1, chloroplastic | 1.336427477 | 0.100 | Photosynthesis, Photorespiration |
| 225 | Q9FNS4 | MBB1 | PsbB mRNA maturation factor | 1.349858824 | 0.802 | mRNA processing via 5'-UTR of psbB |
| 226 | A8I2V9 | LISC | Lipoyl synthase, chloroplastic | 1.377127754 | 0.040 | Protein lipoylation |
| 227 | Q9AR22 | CTH1 | Mg-protoporphyrin IX monomethyl ester | 1.377127754 | 0.008 | Photosynthesis, chlorophyll biosynthesis |
| 228 | A8IB25 | RSSA | 40S ribosomal protein SA | 1.377127754 | 0.062 | Translation |
| 229 | A8I9E8 | CFA45 | Cilia- and flagella-associated protein 45 | 1.404947596 | 0.052 | Cell motality |
| 230 | Q39573 | YPTC5 | GTP-binding protein YPTC5 | 1.404947596 | 0.237 | GTPase activity, protein transport |
| 231 | A8IRK7 | CF251 | Cilia- and flagella-associated protein 251 | 1.404947596 | 0.406 | Cilium movement |
| 232 | P46295 | RS14 | 40S ribosomal protein S14 | 1.404947596 | 0.012 | Translation |
| 233 | Q42684 | SODM | Superoxide dismutase [Mn], mitochondrial | 1.41906754 | 0.624 | Destroys superoxide anion radicals |
| 234 | A8IC48 | URM1 | Ubiquitin-related modifier 1 homolog | 1.41906754 | 0.009 | Endonuclease activity, Intron homing |
| 235 | A8HS48 | RS3A | 40S ribosomal protein S3a | 1.433329435 | 0.871 | Translation |
| 236 | Q8W1K8 | MUT11 | Protein Mut11 | 1.433329435 | 0.019 | Involved in 'Lys-4' histone H3 methylation |
| 237 | A8J8F6 | TEKT | Tektin | 1.433329435 | 0.478 | Assembly of flagella and cilia |
| 238 | Q8HTL7 | RPOB2 | DNA-directed RNA polymerase beta | 1.433329435 | 0.327 | Transcription, DNA-templated |
| 239 | P27766 | DYI3 | Dynein, 70 kDa intermediate chain | 1.476980773 | 0.502 | Cilium movement |
| 240 | Q2XQY7 | IFT57 | Intraflagellar transport protein 57 | 1.476980773 | 0.561 | Maintenance and formation of cilia |
| 241 | A6Q0K5 | CP12 | Calvin cycle protein CP12, chloroplastic | 1.476980773 | 0.024 | Assembly of a core complex PRK/GAPDH |
| 242 | B5BUZ8 | KTU | Protein kintoun | 1.50681778 | 0.510 | Axonemal dynein complex assembly |
| 243 | A8JF70 | ODA1 | Outer dynein arm protein 1 | 1.50681778 | 0.309 | Cilium movement involved in cell motility |
| 244 | A8JC00 | RTCB | RNA-splicing ligase RtcB homolog | 1.50681778 | 0.442 | tRNA splicing |
| 245 | P25387 | GBLP | Guanine nucleotide- protein subunit beta | 1.521961536 | 0.590 | Transcription initiation and termination |
| 246 | A8HPM2 | PSBS1 | Photosystem II protein PSBS1 | 1.537257535 | 0.330 | Nonphotochemical quenching |
| 247 | P08739 | NU5M | NADH-ubiquinone oxidoreductase chain 5 | 1.568312167 | 0.039 | ATP synthesis |
| 248 | P09144 | PSAB | PSI P700 chlorophyll a apoprotein A2 | 1.599994191 | 0.019 | Primery electron donor of PSI |
| 249 | P37255 | PSBB | Photosystem II CP47 RC protein | 1.648721271 | 0.110 | PET in photosystem II |
| 250 | Q8RVC7 | SULP1 | Sulfate permease 1, chloroplastic | 1.648721271 | 0.081 | Uptake and assimilation of sulfate |
| 251 | Q01657 | RSP6 | Flagellar radial spoke protein 6 | 1.665291179 | 0.262 | Cilium assembly |
| 252 | A8I4E9 | CP100 | Cilia- and flagella-associated protein 100 | 1.750672504 | 0.464 | Cell motality, Cillium movement |
| 253 | A8J785 | ATPX | ATP synthase subunit b', chloroplastic | 1.768267039 | 0.008 | ATP synthesis coupled proton transport |
| 254 | P04352 | CALM | Calmodulin | 1.768267039 | 0.351 | Calcium-mediated signalling |
| 255 | P07753 | PSBA | Photosystem II protein D1 | 1.840431425 | 0.003 | Photosynthetic electron transport in PSII |
| 256 | A8IR43 | WDR12 | Ribosome biogenesis protein | 1.840431425 | 0.471 | LSU-rRNA from tricistronic rRNA |
| 257 | P05434 | CATR | Caltractin | 1.840431425 | 0.730 | Cell cycle, Cell division |
| 258 | P10898 | PSBC | PS II CP43 reaction center protein | 1.858928051 | 0.006 | PET in photosystem II |
| 259 | Q39572 | YPTC6 | Ras-related protein YPTC6 | 1.993715528 | 0.265 | GTPase activity |
|  |  |  |  |  |  |  |
|  |  |  | **Up-regulated proteins ( FC ≥ 2.0)** |  |  |  |
|  |  |  |  |  |  |  |
| 260 | Q8LKI3 | ALB32 | ALBINO3-like protein 2, chloroplastic | 2.095935534 | 0.818 | Assembly and activity of LHC I and II. |
| 261 | Q42681 | H31 | Histone H3 type 1 | 2.117000017 | 0.732 | DNA binding, Chromosome stability |
| 262 | A8J3A0 | DRC8 | Dynein regulatory complex protein 8 | 2.159766213 | 0.572 | Regulates microtubule sliding |
| 263 | Q84U21 | RK22 | 50S ribosomal protein L22, chloroplastic | 2.203396474 | 0.034 | Translation, Response to antibiotic |
| 264 | A0A2K3DMP5 | PSBR | Photosystem II protein PSBR, chloroplastic | 2.944679677 | 0.862 | Energy-dependent quenching-NPQ |
| 265 | P12811 | HS22C | Heat shock 22 kDa protein, chloroplastic | 3.455613498 | 0.001 | Protein maturation and degradation |
| 266 | A8I6P9 | SC61B | Protein transport protein Sec61 beta | 4.903749092 | 0.009 | Intracellular protein transport |
| 267 | Q00471 | CYB6 | Cytochrome b6 | 7.53832479 | 0.365 | CEF around PSI and state transitions. |
| 268 | Q42687 | ATPD | ATP synthase delta chain, chloroplastic | 69.40783595 | 0.048 | ATP synthesis coupled proton transport |
|  |  |  |  |  |  |  |
|  |  |  | **Control specific protein** |  |  |  |
|  |  |  |  |  |  |  |
| 269 | P05727 | RK16 | 50S ribosomal protein L16, chloroplastic | Control_W50 |  | Translation |
| 270 | P05726 | RK16 | 50S ribosomal protein L16, chloroplastic | Control_W50 |  | Mitochondrial translation |
| 271 | P07839 | FER | Ferredoxin, chloroplastic | Control_W50 |  | electron transfer activity |
| 272 | P59776 | RR19 | 30S ribosomal protein S19, chloroplastic | Control_W50 |  | Translation |
| 273 | P48268 | PSBE | Cytochrome b559 subunit alpha | Control_W50 |  | PETC, Charge separation |
| 274 | P11660 | RTL | Reverse transcriptase-like protein | Control_W50 |  | cDNA synthesis |
| 275 | P45841 | RL31 | 60S ribosomal protein L31 | Control_W50 |  | Translation, Ribosomal assembly |
| 276 | P15451 | CYC | Cytochrome c | Control_W50 |  | Final protein carrier in mitochondrial ETC |
| 277 | Q42496 | PETM | Cyt b6f complex subunit 7, chloroplastic | Control_W50 |  | CEF around PSI and state transitions. |
| 278 | Q39579 | DYL2 | Dynein 11 kDa light chain | Control_W50 |  | Microtubule-based process |
| 279 | A8IVJ1 | FLTOP | Protein Flattop homolog | Control_W50 |  | Regulator of cilium basal body docking |
| 280 | P37825 | YCX5 | Uncharacterized trnR-chlB intergenic | Control_W50 |  | Tag-protein stability |
| 281 | Q8HTL5 | ATPF | ATP synthase subunit b, chloroplastic | Control_W50 |  | ATP synthesis coupled proton transport |
| 282 | Q8HTL1 | RK5 | 50S ribosomal protein L5, chloroplastic | Control_W50 |  | Translation |
|  |  |  |  |  |  |  |
|  |  |  | **Treated specific protein** |  |  |  |
|  |  |  |  |  |  |  |
| 283 | Q5PU89 | UFM1 | Ubiquitin-fold modifier 1 | Treated_P5_500 |  | Protein ufmylation |
| 284 | P59775 | RR8 | 30S ribosomal protein S8, chloroplastic | Treated_P5_500 |  | Translation |
| 285 | P59774 | rpl36 | 50S ribosomal protein L36, chloroplastic | Treated_P5_500 |  | Translation |
| 286 | A8JJB2 | MOC2A | Molybdopterin synthase sulfur subunit | Treated_P5_500 |  | Mo-molybdopterin cofactor biosynthetic |
| 287 | P50567 | H2A-II | Histone H2A | Treated_P5_500 |  | Chromosomal stability |
| 288 | Q08753 | LI637 | Group 1 truncated hemoglobin LI637 | Treated_P5_500 |  | Oxygen carrier activity |
| 289 | A8ISN6 | ARL3 | ADP-ribosylation factor-like protein 3 | Treated_P5_500 |  | Cytokinesis and cilia signalling |
| 290 | Q9FPQ6 | GP1 | Vegetative cell wall protein gp1 | Treated_P5_500 |  | Major component of outer cell wall W6 |
| 291 | P08740 | ND2 | NADH-ubiquinone oxidoreductase chain 2 | Treated_P5_500 |  | NADH dehydrogenase activity |
| 292 | P32974 | PSBL | Photosystem II reaction center protein L | Treated_P5_500 |  | Photosynthesis, Correct PSII assembly |
| 293 | P04690 | TBB | Tubulin beta-1/beta-2 chain | Treated_P5_500 |  | Microtubule-based process |
| 294 | O20030 | YCF4 | Photosystem I assembly protein Ycf4 | Treated_P5_500 |  | Assembly of the photosystem I complex |
